# Neonatal outcomes in singleton pregnancies conceived by fresh or frozen embryo transfer compared to spontaneous conceptions: a systematic review and meta-analysis

**DOI:** 10.1007/s00404-020-05593-4

**Published:** 2020-05-22

**Authors:** Flavia T. S. Elias, Danielle Weber-Adrian, Jessica Pudwell, Jillian Carter, Mark Walker, Laura Gaudet, Graeme Smith, Maria P. Velez

**Affiliations:** 1 Department of Obstetrics and Gynecology, Kingston General Hospital, Queen’s University, Kingston, ON K7L 2V7 Canada; 2 Health Technology Assessment Program, Oswaldo Cruz Foundation, Brasilia, Brazil; 3 Department of Obstetrics, Gynecology and Newborn Care, University of Ottawa, Ottawa, ON K1H 8M5 Canada; 4 Department of Public Health Sciences, Queen’s University, Kingston, ON K7L 3N6 Canada

**Keywords:** Assisted reproductive technology, Fresh embryo transfer, Frozen embryo transfer, Adverse neonatal outcomes, Meta-analysis, Real-world data

## Abstract

**Purpose:**

The use of assisted reproductive technology (ART) has increased in the last 2 decades and continuous surveillance is needed. This systematic review aims to assess the risk of adverse neonatal outcomes (preterm birth [PTB], low birth weight [LBW], small-for-gestationalage [SGA] and large for gestational-age [LGA]), in singleton pregnancies conceived by fresh or frozen embryo transfer (FET) compared to spontaneous conceptions.

**Methods:**

Cohort studies were identified from MEDLINE, Embase, Cochrane Library (January 2019), and manual search. Meta-analyses were performed to estimate odds ratios (OR) using random effects models in RevMan 5.3 and *I*-squared (*I*^2^) test > 50% was considered as high heterogeneity.

**Results:**

After 3142 titles and abstracts were screened, 1180 full-text articles were assessed, and 14 were eligible. For fresh embryo transfer, the pooled ORs were PTB 1.64 (95% CI 1.46, 1.84); *I*^2^ = 97%; LBW 1.67 (95% CI 1.52, 1.85); *I*^2^ = 94%; SGA 1.46 [95% CI 1.11, 1.92]; *I*^2^ = 99%, LGA 0.88 (95% CI 0.80, 0.87); *I*^2^ = 80%). For frozen, the pooled ORs were PTB 1.39 (95% CI 1.34, 1.44); *I*^2^ = 0%; LBW 1.38 (95% CI 0.91, 2.09); *I*^2^ = 98%; SGA 0.83 (95% CI 0.57, 1.19); *I*^2^ = 0%, LGA 1.57 (95% CI 1.48, 1.68); *I*^2^ = 22%).

**Conclusions:**

When compared with spontaneous pregnancies, fresh, but not frozen was associated with LBW and SGA. Both fresh and frozen were associated with PTB. Frozen was uniquely associated with LGA. Despite improvements in ART protocols in relation to pregnancy rates, attention is needed towards monitoring adverse neonatal outcomes in these pregnancies.

**Electronic supplementary material:**

The online version of this article (10.1007/s00404-020-05593-4) contains supplementary material, which is available to authorized users.

## Introduction

Increased access to assisted reproductive technology (ART) in recent years has benefited those suffering with infertility [1]. Between 1 and 7% of children in industrialized countries are born following ART [2, 3]. These numbers are expected to increase as more countries recognize infertility as an emergent public health priority [3] and are providing ART access through public funding or private health insurance programs [1, 4, 5].

As defined by the international glossary on infertility and fertility care [6], assisted reproductive technology (ART) refers to all interventions that include the in vitro handling of both human oocytes and sperm or of embryos for the purpose of reproduction. This includes, but is not limited to, in vitro fertilization (IVF) and embryo transfer (ET). IVF is defined as a sequence of procedures that involves extracorporeal fertilization of gametes and includes conventional in vitro insemination and intracytoplasmic sperm injection (ICSI, where a single spermatozoon is injected into the oocyte cytoplasm). ET is the placement into the uterus of an embryo at cleavage or blastocyst stage after IVF or ICSI. Embryos can be transferred into the uterus fresh during the same IVF cycle or frozen/thawed embryo transfer (FET) in a subsequent cycle.

It is recognized that ART pregnancies are at a higher risk of adverse neonatal outcomes, such as preterm birth (PTB) and small for gestational age (SGA) [7], congenital malformations [8, 9], stillbirth [10], birth defects [11], and neonatal mortality [12]. While multiple pregnancies as a consequence of ART pose the highest risk of adverse neonatal outcomes [13, 14], singleton pregnancies are also at risk [15, 16]. Whether adverse neonatal outcomes are a consequence of specific ART procedures, due to the baseline infertility diagnosis, or both is still to be determined [17, 18].

Different ART protocols have been rapidly adopted into clinical practice and require constant evaluation of safety [19]. As an example, FET is currently favored over fresh embryo transfer [13] after the publication of a meta-analysis comparing the two techniques head to head. [20]. In this meta-analysis, they did not compare with spontaneous conceptions, and found that compared to fresh embryo transfer, FET resulted in a decreased risk of SGA, low birth weight (LBW) and PTB, and increased risk of large for gestational age (LGA) and high birth weight [20]. While it is important to quantify differences in perinatal outcomes between ART techniques, it is also important to understand how specific ART methods differ from spontaneous conceptions in terms of pregnancy outcomes. This can help to optimize antenatal care for patients pregnant following ART with the ultimate goal of improving pregnancy outcomes.

The objective of this systematic review is to assess the risk of adverse neonatal outcomes in singleton pregnancies conceived by autologous fresh or FET as compared to spontaneous conceptions (SC).

## Materials and methods

### Search strategy

We identified cohort studies assessing the risk of adverse neonatal outcomes in singleton pregnancies after ART compared to spontaneous singleton pregnancies from MEDLINE, Embase, and the Cochrane Library using the OVID interface. Under contracted services, the Knowledge Synthesis Group from the Ottawa Methods Centre at the Ottawa Hospital Research Institute conducted the original search up to June 2017. Subsequently, a senior information specialist from Queen’s University updated the literature search up to January 2019. The MeSH terms used in this search strategy are presented in Supplementary Appendix 01. Additionally, studies referenced in previously published systematic reviews were manually searched and reviewed for inclusion. We did not exclude studies based on language or publication year. This systematic review was registered on the PROSPERO database (CRD# 42017073228). The Preferred Reporting Items for Systematic Reviews and Meta-Analyses (PRISMA) checklist was completed in the preparation of this manuscript (Supplementary Appendix 01).

### Screening and criteria of eligibility

Two authors independently (FTSE, DWA) conducted the abstract and full-text screening, as well as the review of selected full texts (FTSE, DWA). Initial screening was performed based on title and abstract; those screened for inclusion were then reviewed in full. Conflicts were resolved by consensus or by a third team member (MPV).

The inclusion criteria comprised women of all ages who became pregnant after IVF with or without ICSI, using autologous FET or autologous fresh embryo transfer [6]. Only population-based or hospital-based cohort studies that evaluated singleton births as the main population or as a subgroup were included. In evaluating these studies, the inclusion of a control group was defined as a comparison with singleton spontaneous conceptions for which no fertility treatment was used.

The included neonatal outcomes were preterm birth (PTB defined as neonates who were born after at least 20, but before 37 completed weeks of gestation), low birth weight (LBW defined as < 2500 g at birth), large for gestational age (LGA defined as neonates with a weight at or above the 90th percentile for gestational age), small for gestational age (SGA defined as neonates with a weight at or below the 5th or 10th percentile for gestational age, or with a birth weight that is greater than two standard deviations from the average weight for gestational age).

Studies were excluded from evaluation if they had less than 100 patients in any of the groups (because small sample sizes decrease the robustness of the impact measures), used non-invasive ART such as intrauterine insemination (IUI), used treatment that consisted exclusively of pharmacological ovulation induction, had IVF/ICSI using oocyte, embryo or sperm donation or included gestational surrogacy. Studies evaluating singleton births resulting from a vanishing twin pregnancy were also excluded. Additionally, in the case of overlapping studies (as repeat studies of the same population), we only included one study—either the one with the largest sample size or the most recent if the sample size was similar.

### Data collection and data analysis

Two authors (FTSE, JC) manually extracted data from the full text of the included studies using excel spreadsheets. Consensus and accuracy were evaluated by a senior author (MPV). The variables for characterization of the studies were author/year, country, study design (population-based cohort or hospital-based cohort), type of data (cohort prospective, cohort retrospective/linkage/national register), cohort years, and original matching or adjusting factors. The type of ART was defined as IVF if only conventional in vitro insemination was used or ICSI if only intracytoplasmic sperm injection was used. The terms IVF and ICSI were grouped (IVF/ICSI) if the insemination techniques were grouped by the authors or not specified. Type of embryo transfer (Fresh, FET) and outcomes of interest (PTB, LBW, SGA, LGA) were recorded. Exposure and outcome crude data were analyzed using 2 × 2 tables and used to calculate odds ratios (OR, 95% CI). Only dichotomous outcomes were considered. We extracted crude data when the adjusted data were not available. If needed, count data were calculated from provided percentages and these were then rounded off to the nearest integer. The corresponding authors of ten studies were contacted to access crude data or for result clarification and two of them answered.

### Risk of bias and quality assessment

The Newcastle–Ottawa Scale [21, 22] was used by two reviewers (FTS, JP) to complete the quality assessment of the cohorts included (Supplementary Appendix 01). The final scores were summarized to provide an overview of the risk of bias in each study. These scores were classified from 0 to 9, for which a higher score indicates better quality (8 or 9 high, 6 or 7 moderate and less than 5 low quality). The following sources of heterogeneity among the studies were analyzed: characteristics and size of the population, time period of the studies (ranging from 2004 to 2018), and type of registry or cohort (retrospective, prospective, population based, hospital based).

### Statistical analysis and data synthesis

Meta-analyses of measures of association were performed using Review Manager (RevMan) [Computer program] Version 5.3. The measures of association by outcome are reported as odds ratios (OR) with corresponding 95% confidence intervals calculated using random effects models. Random effects models assume heterogeneity in the data and present more conservative estimates. The significance of the pooled OR was estimated using the Mantel–Hanzel statistical method. Measures of heterogeneity were analyzed using the *I*-squared (*I*^2^) statistic test and, when it was > 50% was considered high variation across the studies [23]. Most cohort studies considered potentially confounding variables such as race, maternal age, parity, type of delivery, chronic medical conditions, and previous pregnancy complications. When considering confounding, these variables were controlled for by the use of restriction or matching in the design stage of each individual study. Sensitivity analyses were conducted to explore potential sources of heterogeneity when we could not extract adjusted data, or when the studies were not matched.

## Results

### Search results

**Fig. 1:**
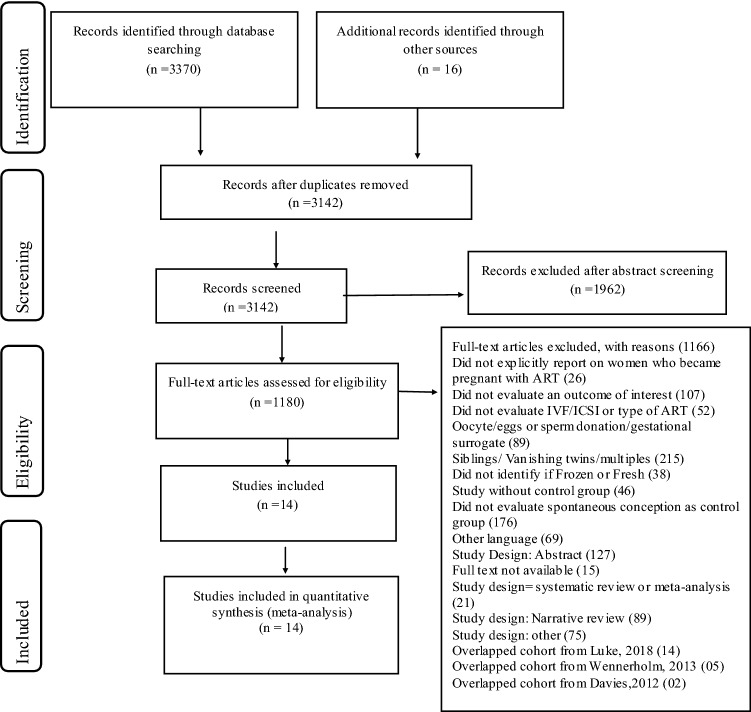
Flowchart identification and selection of included studies

The search strategy identified 3370 records through Medline, Embase, and Cochrane Library databases. Sixteen additional citations were identified by examining the references of the key articles, resulting in 3142 unique records for screening at the title and abstract level. Of these, 1135 full texts were assessed for eligibility, and 14 met the inclusion criteria (Fig. 1: PRISMA flow). Repeat studies of the same population were excluded (*n* = 21) (Fig. 1: PRISMA flow). A complete list of excluded references, organized by reason for exclusion, is provided in Supplementary Appendix 02.

### Characteristics of included studies

Seven studies were population-based cohort studies and conducted in the United States [24, 25], Denmark [26, 27], South Australia [28] and Sweden [29, 30]. Seven studies were hospital-based cohort studies and conducted in Lubeck (Germany) [31], Montreal (Canada) [32], Belgrade (Serbia) [33] St. Louis [34] [35], Amsterdam/Leiden/Nijmegen/Utrecht (Netherland) [36], Oulu/Helsinki (Finland) [37] and Amsterdam (Netherland) [38] (Table 1).

**Table 1:** Overview of the included studies

First author	Type of cohort	Location	Years of the cohort	N per exposure group IVF/ICSI	N per control group Spontaneous conceptions	Search data	Outcomes of interest	(a) Matching factors (b) adjustments in the original analysis made by authors	NOS score	
Buckett, 2007 [32]	Hospital-based retrospective	Montreal (Canada)	1998–2003	Fresh ET (IVF) = 133 Fresh ET [31] = 104	SC = 338	McGill obstetric and neonatal Database [16]	Preterm LBW	(a) Maternal age and parity		9
Cooper, 2011 [35]	Hospital-based retrospective	St. Louis (US)	1999–2009	Fresh ET (IVF/ICSI) = 251 (excluded fetal reduction, frozen embryo and donor oocyte)	SC = 251	Washington University prenatal ultrasound database	Preterm LBW	(a) Maternal age at delivery, maternal race, fetal gender, gestational diabetes, preterm labor, premature rupture of membranes, and pre-eclampsia/eclampsia		8
Crawford, 2017 [24]	Population-based retrospective	Florida, Massachusetts, and Michigan	2000–2010	Fresh ET (IVF/ICSI) = 25,338	SC = 4,301.941	SMART Collaborative linkage and NASS	Preterm LBW	(a) Race and ethnicity (b) Regression analysis: Race/ethnicity, state, age, education, marital status, nativity, smoking, pregestational diabetes, pregestational hypertension, gravidity, parity, initiation of prenatal care in the first trimester, conception with ART		8
Davies, 2012 [28]	Population-based retrospective	South Australia	1986–2002	Fresh ET (IVF) = 1005 Frozen ET(IVF) = 479 Fresh ET [31] = 713 Frozen ET[31] = 226	SC = 293,314	Linkage of two South Australian databases	Preterm			7
Ernstad, 2016 [30]	Population-based retrospective	Sweden	2002–2013	Fresh ET(IVF) = 22,771 Frozen ET(IVF) = 7795 (excluded oocyte donation)	SC = 1,196,394	Linkage of three Swedish databases	Preterm LBW, SGA, LGA			6
Katalinic, 2004 [31]	Hospital-based prospective	Lubeck (Germany)	1998–2000	Fresh ET ICSI = 2055	SC = 7861	Mainz Model Birth Registry and Malformation Monitoring-Centre, Medical records (Exposure)	Preterm			6
Koudstaal, 2000 [36]	Hospital-based retrospective multicentre	Amsterdam, Leiden, Nijmegen, Utrecht (The Netherlands)	1992–2000	Fresh ET(IVF) = 307 (excluded frozen and embryo reduction) Pregnancy > 16 weeks	SC = 307	University Hospital database (Leidein, Nijmegen, Utrecht)	Preterm, LBW (< 2500)	(a) Age, parity, ethnicity, height, weight, smoking, obstetric history, medical history and date of delivery		9
Luke, 2018 [25]	Population-based retrospective	US (14 States)	2004–2013	Fresh ET (IVF/ICSI) = 97,852 Frozen ET (IVF/ICSI) = 27,930	Fertile(SC) = 2,223,647	Linkage SART CORS data and State Vital Records databases	Preterm, LBW, SGA, LGA,	(b) Logistic Regression adjusted for maternal fertility status, age, race and ethnicity, parity, pre-existing conditions (diabetes mellitus and chronic hypertension), pregnancy complications (gestational diabetes and pregnancy hypertension), placental complications (abruption placenta, placenta previa, and other excessive bleeding), plurality at birth (singleton or twin), mode of delivery, State of residence, year of birth, and infant sex		9
Pelkonen, 2010 [37]	Hospital-based retrospective	Oulu, Helsinki (Finland)	1995–2006	Fresh ET (IVF/ICSI) = 2942 Frozen ET (IVF/ICSI) = 1830 (excluded donated eggs, or sperm, or needed preimplantation genetic examinations)	SC = 31,243	Linkage of medical registers with Finnish Medical Birth Register	Preterm, LBW, SGA, LGA	(a) Area of residence and year of birth of the child (b) Maternal age, parity, socioeconomic status, plurality		8
Pinborg, 2010 [26]	Population-based retrospective	Denmark	1995–2007	Fresh ET (IVF/ICSI) = 10,329 Frozen ET (IVF/ICSI) = 957	SC = 4800	Danish Medical Birth Register	Preterm, LBW,	(a) Date and year of birth (b) Logistic regression adjusted by maternal age, parity, child gender, infant year of birth		7
Sazonova, 2012 [29]	Population-based retrospective	Sweden	2002–2006	Frozen single embryo transfer (IVF) = 1533 Fresh single embryo transfer (IVF) = 6047 (excluded oocytes donation)	SC = 571,914	Swedish Medical Birth Registry	Preterm, LBW, SGA, LGA	(b) Year of birth, maternal age, parity, smoking, BMI and years of involuntary childlessness		8
Spijkers, 2017 [38]	Hospital-based retrospective	Amsterdam (The Netherlands)	2006–2015	Frozen ET (IVF/ICSI) = 157 Fresh ET (IVF/ICSI) = 423 (excluded vanishing twins, gestational surrogate and oocytes donations)	SC = 157 SC = 423	University Medical Center Amsterdam	Preterm SGA, LGA	(a) Birth weight, maternal age, gender of the child, parity, gestational age and maternal diabetes mellitus (both pre-existent and gestational)		9
Stojnic, 2013 [33]	Hospital-based retrospective	Belgrade (Serbia)	2006–2010	Fresh ET (IVF/ICSI) = 634 (excluded oocyte donation, frozen and vanishing twins)	SC = 634	Clinical Center of Serbia	Preterm, LBW, SGA, LGA, pregnancy-induced hypertension, gestational diabetes	(a) Age, education, BMI, parity, time (within 1 month), and place of delivery		8
Wennerholm, 2013 [27]	Population-based retrospective	Denmark, Norway, and Sweden	1982–2007	Frozen ET (IVF/ICSI) = 6647 Fresh ET (IVF/ICSI) = 42,242	SC = 288,542	National registries—CoNARTaS group	Preterm, LBW, SGA [< 2 standard deviations (SD)], LGA(> 2D)	(a) Parity (0 versus ≥ 1) and year of birth		9

*SC* spontaneous conceptions, *Fresh ET* fresh embryo transfer, *Frozen ET* frozen embryo transfer, *IVF* in vitro fertilization, *ICS* intracytoplasmic sperm injection, *NOS* Newcastle–Ottawa Score (Supplementary Appendix 01)

The studies varied in terms of adjustment for confounders (Table 1). In general, most studies formed control groups by matching for variables such as maternal age at delivery, parity or birth data [25–27, 32, 33, 35–38]. Other studies adjusted analyses by maternal age at delivery [29, 33], maternal conditions, maternal race/ethnicity, and socioeconomic status [24, 25, 37]. Only three studies [28, 30, 31] did not use adjusted analysis for the outcomes of interest or did not use matched analyses.

### Pregnancy and delivery characteristics

Nine studies did not describe the method used to determine the gestational age at delivery for the included pregnancies. Those that reported a method used first-trimester transvaginal ultrasound for ART pregnancies [35, 37] or second-trimester ultrasound for ART [27, 29, 30] and spontaneous pregnancies [27, 29]. Date of oocyte retrieval for ART and the first day of last menstrual period for spontaneous pregnancies [26] and confirmation of fetal heart beat during ultrasound at 6 weeks of pregnancy [24] were also used.

In most studies, singleton births were included if delivery was after 20 weeks of gestation with the exception of Katalinc et al. [31] and Koudstaal et al. [36], which included deliveries ≥ 16 weeks of gestation, and Stojnic et al.’s study [33], including pregnancies ≥ 26 weeks of gestation.

### Assisted reproductive technology characteristics

All ART pregnancies resulted from conventional in vitro insemination (hereafter IVF) or ICSI followed by fresh or FET. Only three studies of ICSI indicated the type of embryo transfer [10, 31, 32]. Four studies included pregnancies resulting from fresh embryo transfer at the cleavage stage (day 2 or 3 after oocyte retrieval) [32, 33, 37, 38], while two studies included embryos transferred at the blastocyst stage (day 5–6) or at cleavage stage [24, 30]. In FET cycles, embryo transfer was carried out 2–5 days after a positive ovulation test [37, 38] or 6 days after hCG administration [38]. The protocol used for endometrial preparation was reported in only one study [32].

### Fresh embryo transfer using IVF/ICSI

**Fig. 2:**
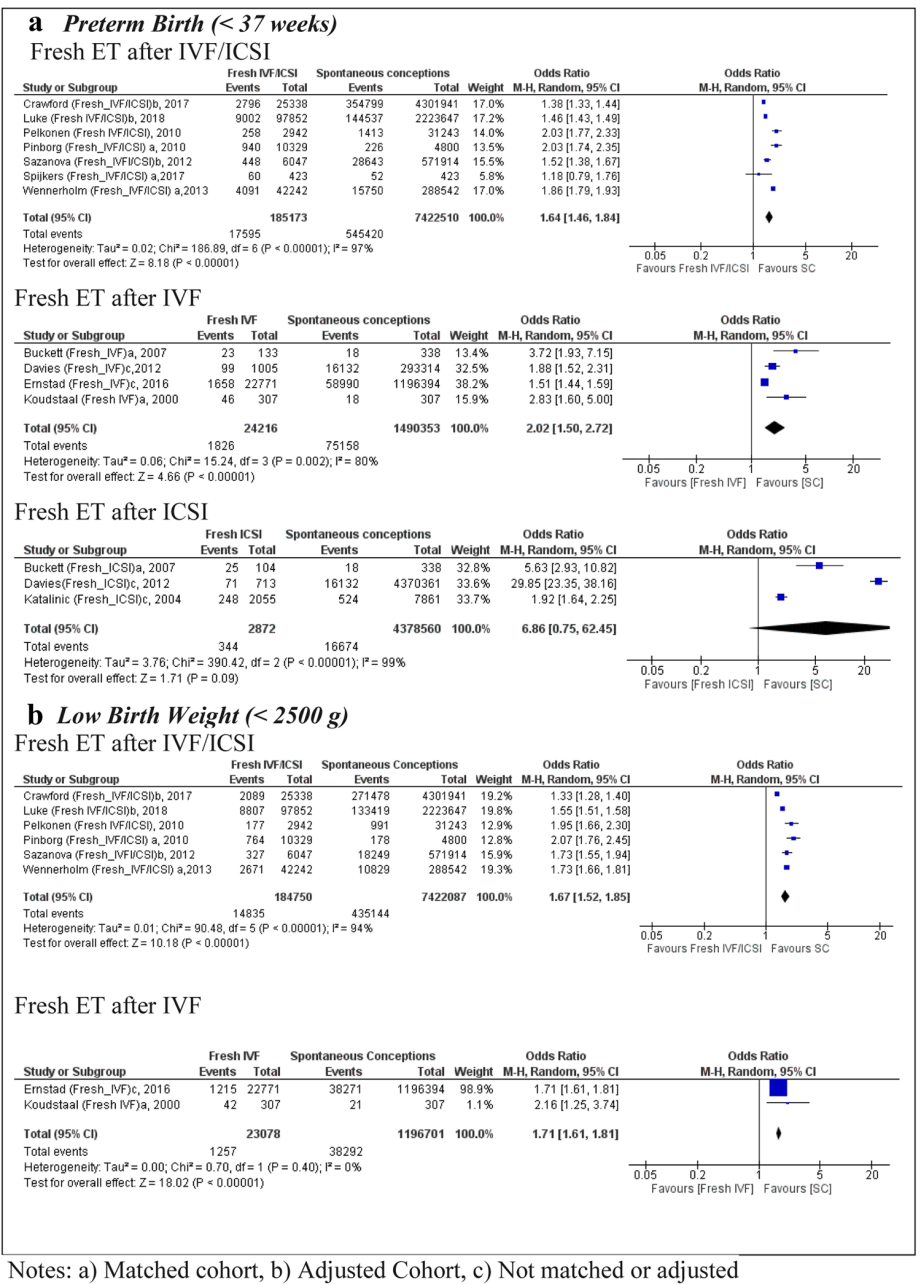
Forest plot of singleton pregnancies resulting from fresh embryo transfer compared to spontaneous conceptions, in relation to **a** preterm birth and **b** low birth weight

In the case of PTB, seven studies using pooled IVF/ICSI with fresh embryo transfer resulted in a sample size of 185,173 births in the exposed group and 7.4 million in the SC group [24–27, 29, 37, 38]. The OR was 1.64 (95% CI 1.46, 1.84) with high heterogeneity (*I*^2^ = 97%) (Fig. 2). The variability among these studies may be explained by the fact that only five of them were matched for maternal characteristics.[17, 24, 27, 37, 38] and the adjusted data were not available for the remaining two population-based studies [25] [29]. Only one study [26] had a moderate quality score in accordance with NOS quality analyses, the rest were high quality (Table 1). A total of four studies [28, 30, 32, 36] were analyzed with respect to PTB after fresh embryo transfer IVF only, resulting in an OR of 2.02 (95% CI 1.50, 2.72) (Fig. 2), which indicates a higher risk in comparison to the analysis of fresh embryo transfer using pooled IVF/ICSI groups. The heterogeneity of the studies for fresh embryo transfer after IVF was *I*^2^ = 80%, suggesting a high variability among the studies. Two studies did not control for confounding factors related to PTB and thus obtained a moderate NOS quality score [28, 30]. When we removed these two studies [28, 30], the chance of PTB was higher compared to SC (OR 3.18; 95% CI 2.07, 4.89) with low heterogeneity (*I*^2^ = 0%) (Supplementary Appendix 03). Only three studies assessed the risk of PTB following fresh embryo transfer using ICSI only [28, 31, 32] and no statistically significant OR was observed (Fig. 2).

The LBW meta-analysis includes six studies [24–27, 29, 37] following fresh embryo transfer after IVF/ICSI compared to SC (*n* = 184,750 vs. 7.4 million of pregnancies), resulting in a pooled OR of 1.67 (95% CI 1.52, 1.85) with high heterogeneity between of the studies *I*^2^ = 94% (Fig. 2). All of the included studies were of high quality according to the NOS score (Table1). When only IVF was used, the pooled OR for LBW was 1.71 (95% CI 1.61, 1.81) [30, 36], with only one of the two studies receiving a NOS score of high quality [36].

**Fig. 3:**
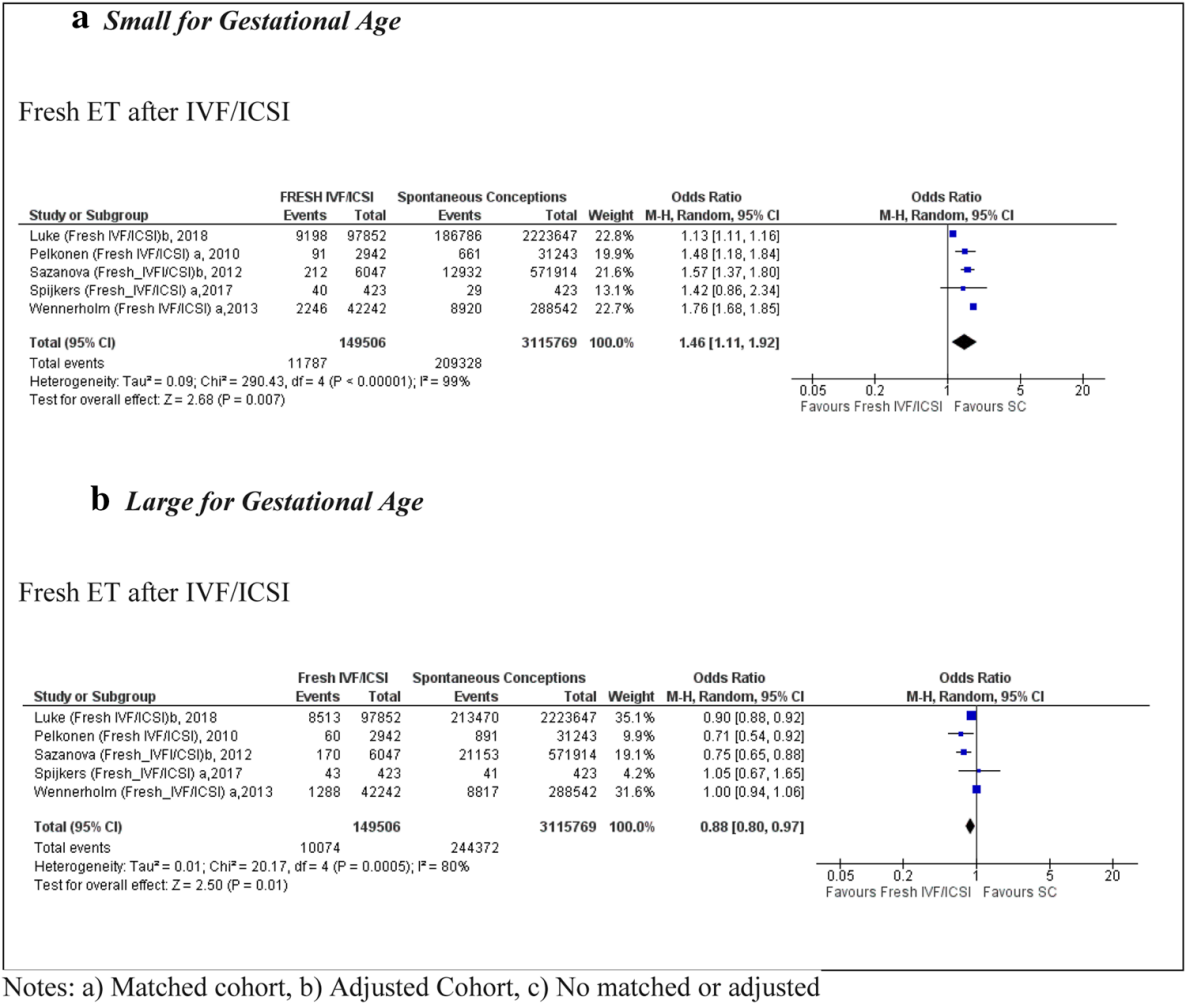
Forest plot of singleton pregnancies resulting from fresh embryo transfer compared to spontaneous conceptions, in relation to **a** small for gestational age and **b** large for gestational age

Five studies [25, 27, 29, 37, 38] reported data on the number of babies that were SGA, including 149,506 pregnancies using fresh embryo transfer after IVF/ICSI compared to 3,115,769 SC (Fig. 3). The pooled OR was 1.46 (95% CI 1.11, 1.92); with high heterogeneity (*I*^2^ = 99%) between the studies and high quality in all of them according to the NOS score. In the analysis of fresh IVF, only one study presented data on SGA outcomes [30] with an OR of 1.51 (95% CI 1.40, 1.63) in comparison to SC.

Five studies [25, 27, 29, 37, 38] reported data on babies that were LGA, including 3,115,769 pregnancies using fresh embryo transfer after IVF/ICSI, resulting in a pooled OR of 0.88 (95% CI 0.80, 0.97) with high heterogeneity (*I*^2^ = 80%) (Fig. 3). Fresh embryo transfer after IVF was included in only one population cohort study [30], indicating an OR of 0.90 (95% CI 0.84, 0.97) for LGA babies.

### Frozen embryo transfer using IVF/ICSI

**Fig. 4:**
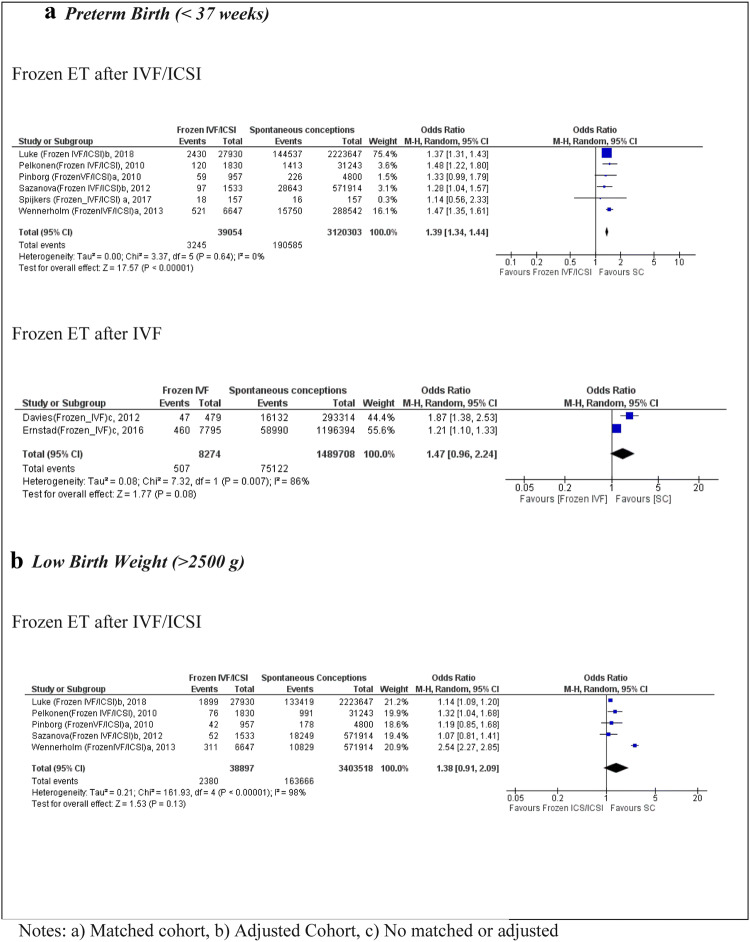
Forest plot of singleton pregnancies resulting from IVF/ICSI in frozen embryo transfer cycles compared to spontaneous conceptions, in relation to **a** preterm birth and **b** low birth weight

Six studies [25–27, 29, 37, 38] reported on PTB after IVF/ICSI in FET cycles leading to a total sample size of 39,054 in the exposure group and 3,120,303 in the SC group (Fig. 4). The pooled analysis showed an OR of 1.39 (95% CI 1.34, 1.44, *I*^2^ 0%), with low heterogeneity. Five of the included studies were high quality according to their NOS score [25, 27, 29, 37, 38], and all of them were matched or adjusted for maternal characteristics (Table 1). The data for PTB after IVF only in FET were presented in two population cohort studies [28, 30], indicating an OR of 1.47 (95% CI 0.96, 2.24) for PTB.

Five studies [25–27, 29, 37] were eligible for the LBW analysis after IVF/ICSI in FET cycles, indicating a non-significant association (OR 1.38; 95% CI 0.91, 2.09) compared to SC; the heterogeneity was high (*I*^2^ = 98%). Only one population cohort study [30] reported data for LBW after IVF in FET cycles, resulting in a non-significant association (OR 1.13; 95% CI 1.00, 1.27).

**Fig. 5:**
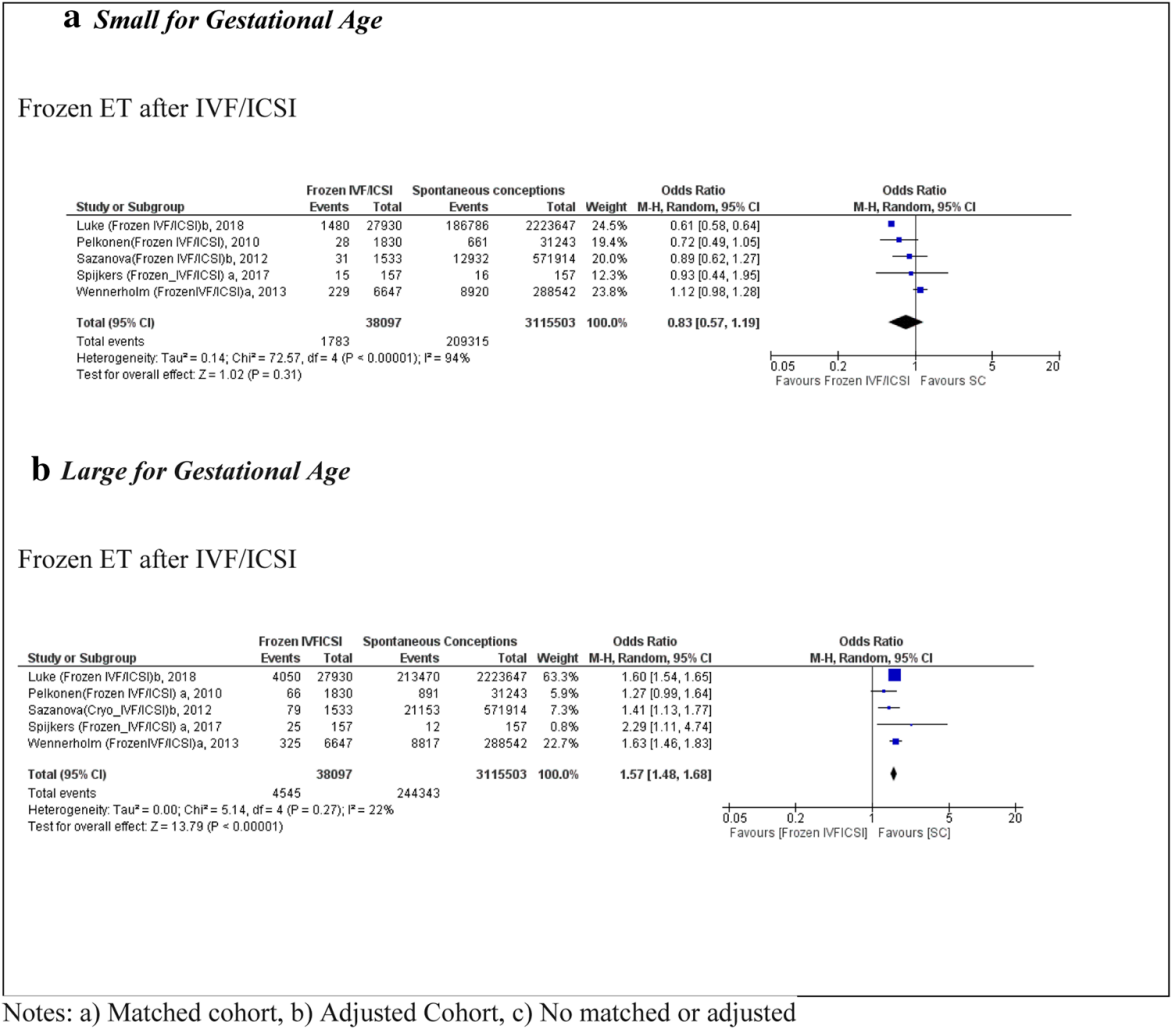
Forest plot of singleton pregnancies resulting from IVF/ICSI in frozen embryo transfer cycles compared to spontaneous conceptions, in relation to **a** small for gestational age and **b** large for gestational age

Five studies [25, 27, 29, 37, 38] were eligible for the SGA analysis following IVF/ICSI in FET cycles, with a total sample size of 38,097 births in the exposed group and 3,115,503 in the SC group. The OR was 0.83 (95% CI 0.57, 1.19) and non-significant (Fig. 5). Only one population cohort study [30] presented data for IVF in FET compared with SC, resulting in an OR of 0.84 (95% CI 0.71, 0.99) for SGA (Table 1).

In relation to LGA after IVF/ICSI in FET cycles, five studies met the inclusion criteria [25, 27, 29, 37, 38]. The OR was 1.57 (95% CI 1.48, 1.68) with low heterogeneity (*I*^2^ = 22%). These studies were of high quality according to their NOS scores, and all were matched or adjusted for maternal characteristics (Table 1). Only one study [30] included data for LGA outcomes for IVF only and FET compared with SC, and reported an OR of 1.41 (95%CI 1.28, 1.55) (Fig. 5).

### Summary of findings

Table 2 summarizes the pooled results by ART. Although there was some variability in outcomes between fresh and FET with IVF/ICSI, IVF only, or ICSI only compared to SC, both modalities of embryo transfer were associated with an increased risk of adverse neonatal outcomes. Most of the studies had high heterogeneity except for the studies included in the PTB and LGA analyses after IVF/ICSI in FET cycles, and LBW after IVF in fresh embryo transfer cycles (Table 2).

**Table 2:** Summary of pooled results by type of ART and OR (95% CI)

Outcomes	*N* births (studies)	*I*^2^ (%)	OR; 95% CI random effect	*N* births (studies)	*I*^2^ (%)	OR; 95% CI random effect
	Fresh ET after IVF/ICSI			Frozen ET after IVF/ICSI		
PTB^a^	7,607.683 (7)	97	1.64 [1.46, 1.84]	3,159.357 (6)	0	1.39 [1.34, 1.44]
LBW^a^	7,606.837 (6)	94	1.67 [1.52, 1.85]	3,442.415 (5)	98	1.38 [0.91, 2.09]
SGA^a^	3,265.275 (5)	99	1.46 [1.11, 1.92]	3,153.600 (5)	94	0.83 [0.57, 1.19]
LGA^a^	3,265.275 (5)	80	0.88 [0.80, 0.97]	3,153.600 (5)	22	1.57 [1.48, 1.68]

	Fresh ET after IVF			Frozen ET after IVF		
PTB^b^	1,514.569 (4)	80	2.02 [1.50, 2.72]	1,497.982 (2)	86	1.47 [0.96, 2.24]
LBW^b^	1,219.779 (2)	0	1.71 [1.61, 1.81]	–	–	–

	Fresh ET after ICSI			Frozen ET after ICSI		
PTB^b^	4,381,432 (3)	99	6.86 [0.75, 62.45]	–	–	–

*IVF* in vitro fertilization, *ICSI* intracytoplasmic sperm injection, *SC* spontaneous conceptions, *fresh ET* fresh embryo transfer, *frozen ET* frozen embryo transfer

^a^Matched or adjusted studies

^b^Some studies no matched or adjusted

### Sensitivity analysis

Sensitivity analyses were conducted to explore potential sources of heterogeneity. We excluded two studies (Luke and Sazanova) for which we could not extract adjusted data for the association between fresh or frozen ET versus SC. When considering only the remaining studies, all of which were matched studies, the results were not significantly changed. For fresh embryo transfer after IVF/ICSI cycles, the pooled OR changes after restriction in the sensitivity analysis were as follows: PTB from 1.64 (95% CI 1.46, 1.84) to 1.71 (95% CI 1.40, 2.07), LBW from 1.67 (95% CI 1.52, 1.85) to 1.73 (95% CI 1.42, 2.10), SGA 1.46 (95% CI 1.11, 1.92) to 1.67 (95% CI 1.47, 1.90), LGA 0.88 (95% CI 0.80, 0.97) to 0.90 (95% CI 0.71, 1.15). (Supplementary Appendix 03). For frozen embryo transfer after IVF/ICSI cycles, the pooled OR changes after restriction in the sensitivity analysis were as follows: PTB from 1.39 (95% CI 1.34, 1.44) to 1.46 (95% CI 1.35,1.58), LBW from 1.38 (95% CI 0.91, 2.09) to 1.61 (95% CI 0.94, 2.27), SGA from 0.83 (95% CI 0.57, 1.19) to 0.94 (95% CI 0.68, 1.30) both non-significant, LGA from 1.57 (95% CI 1.48, 1.68) to 1.54 (95% CI 1.24, 1.91). In these cases, all pooled findings after restriction were similar (Supplementary Appendix 03). When we excluded non-matched studies (Davies et al., and Ernstad et al.), the sensitivity analysis for Preterm in Fresh ET after IVF only, the pooled OR was 3.18 (95% CI 2.07, 4.89) confirming the findings (Supplementary Appendix 03).

## Discussion

### Principal findings

When compared with spontaneous pregnancies, fresh, but not FET in IVF/ICSI cycles was associated with higher rates of LBW and SGA; while both fresh and FET were associated with higher rates of PTB. FET in IVF/ICSI cycles was uniquely associated with higher rates of LGA. When fresh embryo transfers in IVF cycles were analyzed alone, the pooled estimate had high heterogeneity for PTB. The number of eligible studies was insufficient to perform pooled analyses of FET in IVF cycles or ICSI cycles alone for LBW, SGA, and LGA outcomes.

In relation to the type of fertilization technique (conventional in vitro insemination—IVF—versus ICSI), the differences between fresh and FET in ICSI cycles compared to SC remain to be elucidated. The pooled results from this meta-analysis show very large confidence intervals, and the number of studies directly comparing ICSI procedures with spontaneous conceptions is still limited.

### Comparison with other studies

Previous systematic reviews also support an increased risk of adverse neonatal outcomes among singleton IVF/ICSI pregnancies when compared with SC; however, the distinction between fresh and FET is rarely made [39–42]. We did not find previous systematic reviews assessing the risk of SGA or LGA after fresh ET or FET compared to SC. In the case of PTB, our meta-analysis supports a higher risk of PTB after fresh or frozen embryo transfer compared to SC. For fresh embryo transfer, our pooled OR was 1.64 (95% CI 1.46, 1.84), which is lower than the subgroup analysis reported by Carvoretto et al. [16] (OR 1.92, 95% CI 1.67, 2.21). For FET, our OR of 1.39 (95% CI 1.34, 1.44) is higher than in Pinborg et al. [43], which reported an OR of 1.20 (95% CI 0.98, 1.48), and similar to Pandey et al. [41], which reported a RR of 1.39 (95% CI 1.20–1.61). In the case of LBW after FET, our pooled OR of 1.38 (95% CI 0.91, 2.09) differs from Pandey et al.’s RR of 1.27 (95% CI 1.05–1.52) [41], which can be explained by the inclusion of three large studies published after their publication in 2012 [27, 29, 44].

Our meta-analyses add to the literature on the assessment of adverse pregnancy outcomes according to fresh or FET with IVF/ICSI compared to SC; however, the number of studies targeting differences in neonatal outcome between fresh or FET in IVF versus ICSI cycles was insufficient to allow sub-group analysis. This distinction would be clinically relevant given that the use of ICSI is increasing. According to a CDC report in the US [45], the percentage of cycles using ICSI over time has increased from 72% in 2007 to 81% in 2016, even in patients with no male factor infertility [45]. This creates an area of uncertainty in the current practices of ART and their effect on neonatal outcomes, which merits further investigation.

For antenatal care providers, our study highlights the importance of discussing if ART conceived the pregnancy, and if so, the type of embryo transfer to provide estimates of the neonatal risks compared to spontaneous conceptions and monitor the pregnancy accordingly. In terms of ART procedures, our results highlight the importance of discussing embryo transfer options with women seeking infertility treatments. For instance, in addition to decreasing the rates of LBW and SGA in comparison with fresh embryo transfer, FET results in a lower risk of ovarian hyperstimulation syndrome, perinatal morbidity, and maternal morbidity [46]. However, further studies are needed to identify factors contributing to LGA after FET, and if interventions during pregnancy could mitigate this outcome.

### Strengths and limitations

It was not possible to use statistical approaches that re-expressed adjusted odds ratios in some studies [25, 29] because of the absence of the adjusted data for the association between fresh or frozen ET versus SC. As a result, we used crude data without the author's adjustment variables. Despite this limitation, the sensitivity analysis using only the matched studies did not indicate significant changes in the analysis outcomes. An additional consideration is that some of the outcomes could be influenced by maternal characteristics such as ethnicity/race/socioeconomic status, and infertility diagnosis. Ethnicity, race, and socioeconomic status were reported in four of the included studies [30, 31, 35, 36], while one study [36] analyzed infertility diagnosis as a confounding variable. Furthermore, the pooling of reported IVF and ICSI data (IVF/ICSI) creates a limitation in evaluating adverse neonatal outcomes based on the fertilization technique.

As well, we did not assess the influence of an extended blastocyst culture versus cleavage stage transfer, the impact of different culture media the method of freezing, the regimen for transfer in a frozen/thawed cycle (spontaneous vs. hormonal replacement therapy), which may also have an effect on the neonatal outcomes [47–50].

### Implications for clinical practices and research

The results of our meta-analyses suggest that FET embryo transfer use in IVF/ICSI is associated with a lower risk of LBW and SGA neonatal outcomes. Although fresh and FET were both associated with increased rates of PTB in comparison with SC, the OR for the fresh embryo IVF/ICSI was higher than that of the frozen embryo IVF/ICSI group (OR 1.64, 95% CI 1.46–1.84 versus OR 1.39, 95% CI 1.34–1.44, respectively). Conversely, for reasons that remain to be elucidated, the FET with IVF/ICSI was exclusively associated with a higher OR of LGA babies.

The role of IVF versus ICSI on neonatal outcomes in comparison with fresh versus FET is also unclear, given that there are insufficient studies that have analyzed these risk factors independently. Additionally, maternal characteristics such as weight, smoking, infertility diagnosis, subfertility factors, race, socioeconomic status, and ethnicity could play a role in determining adverse neonatal risk factors after ART treatment and provides an area of research which merits further investigation.

## Conclusion

IVF/ICSI treatments using fresh or FET are associated with higher rates of PTB in comparison to spontaneously conceived pregnancies. In addition, Fresh embryo transfer is associated with higher rates of LBW and SGA, while FET is also associated with an increased risk of LGA.

## Electronic supplementary material

Below is the link to the electronic supplementary material.

Supplementary file1 (DOCX 56 kb)

Supplementary file2 (DOCX 142 kb)

Supplementary file3 (DOCX 109 kb)
