# Supplementary material for: Neonatal outcomes in singleton pregnancies conceived by fresh or frozen embryo transfer compared to spontaneous conceptions: a systematic review and meta-analysis
Source: Arch Gynecol Obstet. 2020 May 22;302(1):31–45. doi: 10.1007/s00404-020-05593-4 (PMC7266861; doi:10.1007/s00404-020-05593-4)
Supplement: Supplementary file 1 — Supplementary file1 (DOCX 56 kb) [file 404_2020_5593_MOESM1_ESM.docx]

**Supplementary Appendix 01 - Neonatal outcomes in singleton pregnancies conceived by fresh or frozen embryo transfer compared to spontaneous conceptions: a systematic review and meta-analysis of cohort studies**

1. Database(s): **Embase Classic+Embase**1947 to 2019 October 02**, Ovid MEDLINE(R) and Epub Ahead of Print, In-Process & Other Non-Indexed Citations, Daily and Versions(R)**1946 to October 02, 2019
   Search Strategy:

**# Searches**

1 Pregnancy/

2 exp Pregnancy Complications/

3 Pregnant Women/

4 exp Pregnancy Trimesters/

5 pregnan*.tw,kw.

6 Prenatal Care/

7 (prenatal* or antenatal* or ante natal* or antepartum or ante partum).tw,kw.

8 or/1-7

9 exp Reproductive Techniques, Assisted/

10 (assist* adj3 (conceiv* or concepti* or reproducti*)).tw,kw.

11 (reproducti* adj3 (technic or technics or technique*)).tw,kw.

12 ART.tw,kw.

13 ((artificial* or intrauterine or intra-uterine) adj3 inseminat*).tw,kw.

14 IUI.tw,kw.

15 ("In Vitro" adj3 (fertili* or reproducti*)).tw,kw.

16 IVF.tw,kw.

17 (test-tube* adj3 (baby or babies or fertili* or reproducti*)).tw,kw.

18 (mitochondrial replacement adj3 (technic or technics or technique* or therap* or treat*)).tw,kw.

19 ((pronuclear or pro-nuclear or spindle) adj3 (transfer technic* or transfer technique*)).tw,kw.

20 (donor adj3 (conceiv* or conception*)).tw,kw.

21 exp Embryo Transfer/

22 ((embryo or embryos or blastocyst* or cleavage*) adj5 (transfer* or transplant*)).tw,kw.

23 ((embryo or embryos or blastocyst* or cleavage*) adj5 (cryotransfer* or cryo-transfer* or cryotransplant* or cryo-transplant*)).tw,kw.

24 ((embryo or embryos or blastocyst* or cleavage*) and (eSet? or dSet? or eDet? or eFet? or eMet?)).tw,kw.

25 ("IVF-SET" or "IVF-DET" or "IVF-FET" or "IVF-MET" or "IVF-eSET" or "IVF-dSET" or "IVF-eDET" or "IVF-eFET" or "IVF-eMET").tw,kw.

26 ("ICSI-SET" or "ICSI-DET" or "ICSI-FET" or "ICSI-MET" or "ICSI-eSET" or "ICSI-dSET" or "ICSI-eDET" or "ICSI-eFET" or "ICSI-eMET").tw,kw.

27 ((embryo or embryos or blastocyst* or cleavage* or elective or non-elective) adj3 (SET? or SBT? or DET? or DBT? or FET? or FBT? or MET? or MBT? or 2BT?)).tw,kw.

28 (gamete adj (intrafallopian or intra-fallopian) adj transfer*).tw,kw.

29 ("in vitro" adj3 matur*).tw,kw. and (exp Oocytes/ or oocyte*.tw,kw.)

30 ((oocyte* or ova or ovum) adj3 (donat* or donor*)).tw,kw.

31 (zygote* adj (intrafallopian or intra-fallopian) adj transfer*).tw,kw.

32 ((pronuclear or pro-nuclear) adj stage adj2 transfer*).tw,kw.

33 PROST.tw,kw.

34 ZIFT.tw,kw.

35 (ovar* adj3 stimulat*).tw,kw.

36 (ovulat* adj3 (induc* or stimulat*)).tw,kw.

37 (sperm adj2 (inject* or microinject* or micro-inject*) adj3 (intracytoplasmic or intra-cyctoplasmic)).tw,kw.

38 ICSI.tw,kw.

39 Surrogate Mothers/

40 ((surrogate or gestation* or host) adj2 (carrier* or mother*)).tw,kw.

41 (surrogate adj2 pregnan*).tw,kw.

42 exp Infertility/th

43 ((infertil* or subfecund* or sub-fecund* or subfertil* or sub-fertil*) adj5 (manag* or therap* or treat*)).tw,kw.

44 "IVF-ET".tw,kw.

45 or/9-44

46 8 and 45

47 exp Risk/

48 (risk or risked or risks or risky or risking or risk-related).tw,kw.

49 predict*.tw,kw.

50 logistic*.tw,kw.

51 (logit* adj1 model*).tw,kw.

52 Prevalence/

53 prevalen*.tw,kw.

54 Pregnancy Complications/ep

55 Pregnancy Outcome/ep

56 or/47-55

57 46 and 56

58 exp Obstetric Labor, Premature/

59 ((labor* or labour* or birth* or deliver*) adj3 (preterm* or pre-term* or prematur* or pre-matur*)).tw,kw.

60 (PTL or sPTL or PTB or sPTB or PTD or sPTD).tw,kw.

61 Uterine Contraction/

62 ((uterus* or uterine) adj3 contraction*).tw,kw.

63 ((cervical or cervix*) adj3 (dilat* or ripen*)).tw,kw.

64 (preterm* or pre-term* or prematur* or pre-matur*).tw,kw.

65 or/61-63

66 64 and 65

67 or/58-60,66

68 exp Infant, Low Birth Weight/

69 ((low birth weight or low birthweight) adj2 (infant? or baby or babies or neonat* or newborn?)).tw,kw.

70 (LBW or VLBW).tw,kw.

71 ("small for date" or "small for dates").tw,kw.

72 (small* adj3 gestational age?).tw,kw.

73 (SGA or VSGA).tw,kw.

74 (large* adj3 gestational age?).tw,kw.

75 (LGA or VLGA).tw,kw.

76 or/68-75

77 67 or 76

78 57 and 77

79 exp Animals/ not (exp Animals/ and Humans/)

80 78 not 79

81 (comment or editorial or interview or news or newspaper article).pt.

82 (letter not (letter and randomized controlled trial)).pt.

83 80 not (81 or 82)

84 83 use ppezv

85 pregnancy/

86 exp pregnancy complication/

87 pregnant woman/

88 first trimester pregnancy/ or second trimester pregnancy/ or third trimester pregnancy/

89 pregnan*.tw,kw.

90 prenatal care/

91 (prenatal* or antenatal* or ante natal* or antepartum or ante partum).tw,kw.

92 or/85-91

93 exp infertility therapy/

94 (assist* adj3 (conceiv* or concepti* or reproducti*)).tw,kw.

95 (reproducti* adj3 (technic or technics or technique*)).tw,kw.

96 ART.tw,kw.

97 ((artificial* or intrauterine or intra-uterine) adj3 inseminat*).tw,kw.

98 IUI.tw,kw.

99 ("In Vitro" adj3 (fertili* or reproducti*)).tw,kw.

100 IVF.tw,kw.

101 "IVF-ET".tw,kw.

102 (test-tube* adj3 (baby or babies or fertili* or reproducti*)).tw,kw.

103 (mitochondrial replacement adj3 (technic or technics or technique* or therap* or treat*)).tw,kw.

104 ((pronuclear or pro-nuclear or spindle) adj3 (transfer technic* or transfer technique*)).tw,kw.

105 (donor adj3 (conceiv* or conception*)).tw,kw.

106 ((embryo or embryos or blastocyst* or cleavage*) adj5 (transfer* or transplant*)).tw,kw.

107 ((embryo or embryos or blastocyst* or cleavage*) adj5 (cryotransfer* or cryo-transfer* or cryotransplant* or cryo-transplant*)).tw,kw.

108 ((embryo or embryos or blastocyst* or cleavage*) and (eSet? or dSet? or eDet? or eFet? or eMet?)).tw,kw.

109 ("IVF-SET" or "IVF-DET" or "IVF-FET" or "IVF-MET" or "IVF-eSET" or "IVF-dSET" or "IVF-eDET" or "IVF-eFET" or "IVF-eMET").tw,kw.

110 ("ICSI-SET" or "ICSI-DET" or "ICSI-FET" or "ICSI-MET" or "ICSI-eSET" or "ICSI-dSET" or "ICSI-eDET" or "ICSI-eFET" or "ICSI-eMET").tw,kw.

111 ((embryo or embryos or blastocyst* or cleavage* or elective or non-elective) adj3 (SET? or SBT? or DET? or DBT? or FET? or FBT? or MET? or MBT? or 2BT?)).tw,kw.

112 (gamete adj (intrafallopian or intra-fallopian) adj transfer*).tw,kw.

113 ("in vitro" adj3 matur*).tw,kw. and (exp oocyte/ or oocyte*.tw,kw.)

114 ((oocyte* or ova or ovum) adj3 (donat* or donor*)).tw,kw.

115 (zygote* adj (intrafallopian or intra-fallopian) adj transfer*).tw,kw.

116 ((pronuclear or pro-nuclear) adj stage adj2 transfer*).tw,kw.

117 PROST.tw,kw.

118 ZIFT.tw,kw.

119 (ovar* adj3 stimulat*).tw,kw.

120 (ovulat* adj3 (induc* or stimulat*)).tw,kw.

121 (sperm adj2 (inject* or microinject* or micro-inject*) adj3 (intracytoplasmic or intra-cyctoplasmic)).tw,kw.

122 ICSI.tw,kw.

123 ((surrogate or gestation* or host) adj2 (carrier* or mother*)).tw,kw.

124 (surrogate adj2 pregnan*).tw,kw.

125 ((infertil* or subfecund* or sub-fecund* or subfertil* or sub-fertil*) adj5 (manag* or therap* or treat*)).tw,kw.

126 or/93-125

127 92 and 126

128 risk/

129 risk assessment/

130 risk factor/

131 (risk or risked or risks or risky or risking or risk-related).tw,kw.

132 predict*.tw,kw.

133 logistic*.tw,kw.

134 (logit* adj1 model*).tw,kw.

135 prevalence/

136 prevalen*.tw,kw.

137 exp pregnancy complication/ep

138 or/128-137

139 127 and 138

140 premature labor/

141 ((labor* or labour* or birth* or deliver*) adj3 (preterm* or pre-term* or prematur* or pre-matur*)).tw,kw.

142 (PTL or sPTL or PTB or sPTB or PTD or sPTD).tw,kw.

143 uterus contraction/

144 ((uterus* or uterine) adj3 contraction*).tw,kw.

145 ((cervical or cervix*) adj3 (dilat* or ripen*)).tw,kw.

146 (preterm* or pre-term* or prematur* or pre-matur*).tw,kw.

147 or/143-145

148 146 and 147

149 or/140-142,148

150 or/140-142,148

151 exp low birth weight/

152 ((low birth weight or low birthweight) adj2 (infant? or baby or babies or neonat* or newborn?)).tw,kw.

153 (LBW or VLBW).tw,kw.

154 ("small for date" or "small for dates").tw,kw.

155 (small* adj3 gestational age?).tw,kw.

156 large for gestational age/

157 (large* adj3 gestational age?).tw,kw.

158 (LGA or VLGA).tw,kw.

159 or/151-158

160 150 or 159

161 139 and 160

162 exp animal experimentation/ or exp animal model/ or exp animal experiment/ or nonhuman/ or exp vertebrate/

163 exp human/ or exp human experimentation/ or exp human experiment/

164 162 not 163

165 161 not 164

166 editorial.pt.

167 letter.pt. not (randomized controlled trial/ and letter.pt.)

168 165 not (166 or 167)

169 168 use emczd

170 84 or 169

171 remove duplicates from 170

1. limit 171 to yr="2017 -Current"

| **Section/topic** | **#** | **Checklist item** | **Reported on page #** |
| --- | --- | --- | --- |
| **TITLE** | | |  |
| Title | 1 | Identify the report as a systematic review, meta-analysis, or both. | Title page |
| **ABSTRACT** | | |  |
| Structured summary | 2 | Provide a structured summary including, as applicable: background; objectives; data sources; study eligibility criteria, participants, and interventions; study appraisal and synthesis methods; results; limitations; conclusions and implications of key findings; systematic review registration number. | Abstract  page |
| **INTRODUCTION** | | |  |
| Rationale | 3 | Describe the rationale for the review in the context of what is already known. | Page 1 |
| Objectives | 4 | Provide an explicit statement of questions being addressed with reference to participants, interventions, comparisons, outcomes, and study design (PICOS). | Page 2 |
| **METHODS** | | |  |
| Protocol and registration | 5 | Indicate if a review protocol exists, if and where it can be accessed (e.g., Web address), and, if available, provide registration information including registration number. | Page 2 |
| Eligibility criteria | 6 | Specify study characteristics (e.g., PICOS, length of follow-up) and report characteristics (e.g., years considered, language, publication status) used as criteria for eligibility, giving rationale. | Page 2 |
| Information sources | 7 | Describe all information sources (e.g., databases with dates of coverage, contact with study authors to identify additional studies) in the search and date last searched. | Page 2 |
| Search | 8 | Present full electronic search strategy for at least one database, including any limits used, such that it could be repeated. | Supplementary Appendix1 |
| Study selection | 9 | State the process for selecting studies (i.e., screening, eligibility, included in systematic review, and, if applicable, included in the meta-analysis). | Page 2,3 |
| Data collection process | 10 | Describe method of data extraction from reports (e.g., piloted forms, independently, in duplicate) and any processes for obtaining and confirming data from investigators. | Page 3 |
| Data items | 11 | List and define all variables for which data were sought (e.g., PICOS, funding sources) and any assumptions and simplifications made. | Page 3 |
| Risk of bias in individual studies | 12 | Describe methods used for assessing risk of bias of individual studies (including specification of whether this was done at the study or outcome level), and how this information is to be used in any data synthesis. | Page 3,4 |
| Summary measures | 13 | State the principal summary measures (e.g., risk ratio, difference in means). | Page 4 |
| Synthesis of results | 14 | Describe the methods of handling data and combining results of studies, if done, including measures of consistency (e.g., I^2^) for each meta-analysis. | Figure 2,3,4,5 |

Page 1 of 2

| **Section/topic** | **#** | **Checklist item** | **Reported on page #** |
| --- | --- | --- | --- |
| Risk of bias across studies | 15 | Specify any assessment of risk of bias that may affect the cumulative evidence (e.g., publication bias, selective reporting within studies). | Page 4, Supplementary Appendix 01 |
| Additional analyses | 16 | Describe methods of additional analyses (e.g., sensitivity or subgroup analyses, meta-regression), if done, indicating which were pre-specified. | n/a |
| **RESULTS** | | |  |
| Study selection | 17 | Give numbers of studies screened, assessed for eligibility, and included in the review, with reasons for exclusions at each stage, ideally with a flow diagram. | Page 4  Figure 1 |
| Study characteristics | 18 | For each study, present characteristics for which data were extracted (e.g., study size, PICOS, follow-up period) and provide the citations. | Pages 5,6 Table 1 |
| Risk of bias within studies | 19 | Present data on risk of bias of each study and, if available, any outcome level assessment (see item 12). | Supplementary Appendix 01 |
| Results of individual studies | 20 | For all outcomes considered (benefits or harms), present, for each study: (a) simple summary data for each intervention group (b) effect estimates and confidence intervals, ideally with a forest plot. | Figure 2,3,4,5 |
| Synthesis of results | 21 | Present results of each meta-analysis done, including confidence intervals and measures of consistency. | Pages 6,7  Figure 2,3,4,5 |
| Risk of bias across studies | 22 | Present results of any assessment of risk of bias across studies (see Item 15). | Page 6,7,8 |
| Additional analysis | 23 | Give results of additional analyses, if done (e.g., sensitivity or subgroup analyses, meta-regression [see Item 16]). | Page 7 |
| **DISCUSSION** | | |  |
| Summary of evidence | 24 | Summarize the main findings including the strength of evidence for each main outcome; consider their relevance to key groups (e.g., healthcare providers, users, and policy makers). | Page 9 |
| Limitations | 25 | Discuss limitations at study and outcome level (e.g., risk of bias), and at review-level (e.g., incomplete retrieval of identified research, reporting bias). | Page 9 |
| Conclusions | 26 | Provide a general interpretation of the results in the context of other evidence, and implications for future research. | Page 10,11 |
| **FUNDING** | | |  |
| Funding | 27 | Describe sources of funding for the systematic review and other support (e.g., supply of data); role of funders for the systematic review. | Page 11 |

*From:*  Moher D, Liberati A, Tetzlaff J, Altman DG, The PRISMA Group (2009). Preferred Reporting Items for Systematic Reviews and Meta-Analyses: The PRISMA Statement. PLoS Med 6(7): e1000097. doi:10.1371/journal.pmed1000097 For more information, visit: **www.prisma-statement.org**. Page 2 of 2
