# Supplementary material for: Neonatal outcomes in singleton pregnancies conceived by fresh or frozen embryo transfer compared to spontaneous conceptions: a systematic review and meta-analysis
Source: Arch Gynecol Obstet. 2020 May 22;302(1):31–45. doi: 10.1007/s00404-020-05593-4 (PMC7266861; doi:10.1007/s00404-020-05593-4)
Supplement: Supplementary file 2 — Supplementary file2 (DOCX 142 kb) [file 404_2020_5593_MOESM2_ESM.docx]

# Appendix 2

# Neonatal outcomes in singleton pregnancies conceived using fresh or frozen embryo transfer compared to spontaneous conceptions: a systematic review and meta-analysis of cohort studies (Date last searched January, 2019)

# Excluded studies (final list)

## Did not explicitly report of women who became pregnant with ART/ women with specific diseases

1. A. Hashimoto, T. Iriyama, S. Sayama, T. Nakayama, A. Komatsu, A. Miyauchi, O. Nishii, T. Nagamatsu, Y. Osuga and T. Fujii. Adenomyosis and adverse perinatal outcomes: increased risk of second trimester miscarriage, preeclampsia, and placental malposition. 2017. 1-6.
2. Fuchs F, Monet B, Ducruet T, Chaillet N, Audibert F. Effect of Maternal Age on the Risk of Preterm Birth. Obstetrical & Gynecological Survey. 2018. 73(6):340-2.
3. W. A. Agger, C. W. Schauberger, J. K. Burmester and S. K. Shukla. Developing Research Priorities for Prediction and Prevention of Preterm Birth. 2016. 14 (3-4): 123-125.
4. Jefride Y, Barati M, Shojaei K, Aberoumand S, Masihi S, Momengaribvand M. Cerebroplacental Ratio in Non Small for Gestational Age Fetuses at 28 to 38 Weeks of Gestation. International Journal of Women's Health and Reproduction Sciences. 2018. 6: (3)302-306.
5. J. Johnston. Little people, big problems. 2016. 46 (1).
6. P. J. Danielian and M. H. Hall. The epidemiology of prematurity. 1996. 6 (3): 133-136.
7. H. Falconer, O. Stephansson, F. Granath and H. Kieler. Endometriosis and risk of adverse pregnancy outcome. 2009. 24: i98.
8. T. Bernardi, M. Radosa, A. Lauten, A. Kavallaris, J. Herrmann, O. Camara, A. Weisheit and I. Runnebaum. Safety of vaginal birth after laparoscopic myomectomy: A critical view. 2009. 107: S510.
9. M. Naveiro Fuentes, J. Gongora Rodriguez, S. Ruiz Duran, M. T. Maroto Martin, A. Alkourdi Martinez and A. Puertas Prieto. Risk factors and pregnancy outcomes in velamentous cord insertion births. 2014. 27: 31.
10. A. Colver, C. Fairhurst and P. O. D. Pharoah. Cerebral Palsy. 2014. 69 (8): 447-449.
11. A. Rodolakis, N. Thomakos, I. Koutroumpa, M. Sotiropoulou, D. Haidopoulos, G. Vlachos and D. Loutradis. Abdominal radical trachelectomy for early stage cervical cancer: Fertility sparing approach. 2015. 137: 30-31.
12. M. A. Mainigi, S. Schon, F. Wang, T. Ord, R. Feng and C. Coutifaris. Superovulation alters the expression of endometrial genes critical to angiogenesis, tissue remodeling and placentation. 2014. 102 (3 SUPPL. 1): e132.
13. M. Vijayasree. Study of maternal and fetal outcome in twin gestation at tertiary care referral centre. 2014. 27: 430-431.
14. C. V. Ananth and S. P. Chauhan. Epidemiology of twinning in developed countries. 2012. 36 (3): 156-61-.
15. J. Haas, Y. Yinon, K. Meridor and R. Orvieto. Pregnancy outcome in severe OHSS patients following ascitic/plerural fluid drainage. 2014. 7: 56.
16. M. C. Pitter, A. R. Gargiulo, L. M. Bonaventura, J. S. Lehman and S. S. Srouji. Pregnancy outcomes following robot-assisted myomectomy. 2013. 28 (1): 99-108.
17. C. H. Kim, N. R. Abu-Rustum, D. S. Chi, G. J. Gardner, M. M. Leitao, Jr., J. Carter, R. R. Barakat and Y. Sonoda. Reproductive outcomes of patients undergoing radical trachelectomy for early-stage cervical cancer. 2012. 125 (3): 659-66.
18. A. Schneider, E. Erdemoglu, V. Chiantera, N. Reed, P. Morice, A. Rodolakis, D. Denschlag and V. Kesic. Clinical recommendation radical trachelectomy for fertility preservation in patients with early-stage cervical cancer. Int J Gynecol Cancer 2012; 22:659-666.
19. M. Lidar and P. Langevitz. Pregnancy issues in scleroderma. 2012. 11 (6-7): 515-9.
20. J. J. Henderson, O. A. McWilliam, J. P. Newnham and C. E. Pennell. Preterm birth aetiology 2004-2008. Maternal factors associated with three phenotypes: spontaneous preterm labour, preterm pre-labour rupture of membranes and medically indicated preterm birth. 2012. 25 (6): 642-7.
21. I. M. Usta and A. H. Nassar. Advanced maternal age. Part I: obstetric complications. 2008. 25 (8): 521-34.
22. A. M. Jorgensen. Late preterm birth: a rising trend: part one of a two-part series. 2008. 12 (4): 308-315.
23. M. J. Simchen, Y. Yinon, O. Moran, E. Schiff and E. Sivan. Pregnancy outcome after age 50. 2006. 108 (5): 1084-8.
24. I. Blickstein and L. G. Keith. Iatrogenic multiple pregnancy. 2002. 7 (3): 169-76.
25. R. M. Henig. Pandora's baby. 2003. 288 (6): 62-7.
26. J. G. Schenker. Clinical aspects of ovarian hyperstimulation syndrome. 1999. 85 (1): 13-20.

## Did not evaluate an outcome of interest

1. Bazarra-Fernandez. Contraception in the young age and pregnancy in the days of old for the elderly through art: Is it the right way?. 2010. 15: 111.
2. B. M. Norgard,​ P. V. Larsen,​ J. Fedder,​ P. S. De Silva,​ M. D. Larsen and S. Friedman. Live birth and adverse birth outcomes in women with ulcerative colitis and Crohn's disease receiving assisted reproduction: A 20-year nationwide cohort study. 2016. 65 (5): 767-776.
3. Y. Yi, X. Li, Y. Ouyang, F. Gong, G. Lin and G. Lu. The optimal number of embryo transferred for patients with didelphys uterus: Following 115 cases after in vitro fertilization embryo transfer (IVF-ET). 2016. 31: i465-i466.
4. T. Wainstock, A. Walfisch, I. Shoham-Vardi, I. Segal, A. Harlev, R. Sergienko, D. Landau and E. Sheiner. Fertility treatments and pediatric neoplasms of the offspring: results of a population-based cohort with a median follow-up of 10 years. 2017. 216 (3): 314.e1-314.e14.
5. Sekhon L, Lee JA, Duke M, Briton-Jones C, Flisser E, Copperman AB. The duration of cryostorage of biopsied embryos neither impacts implantation potential nor surrogate markers of placentation. Fertility and Sterility. 2017. 108(3):e178.
6. M. Carvalho, S. Mota, F. Leal, I. Cordeiro, A. Aguiar, J. Nunes, C. Rodrigues, A. P. Soares, S. Sousa and C. Calhaz-Jorge. Are double embryo transfers' outcome influenced by the quality of the second embryo?. 2016. 31: i206.
7. G. Oron, S. Rona, O. Sapir, L. Hirsch, Y. Shufaro, B. Fisch and A. Ben-Haroush. Is endometrial thickness associated with intrauterine growth restriction or placental related pregnancy complications in fresh IVF cycles?. 2016. 31: i49.
8. Holman MA, Ainsworth A, Hathcock M, Borowski K, Coddington C. 444: Effect of fresh versus frozen embryo transfer on fetal growth parameters. American Journal of Obstetrics & Gynecology. 2018. 218(1):S268-9Harlev, T. Wainstock, A. Walfisch, I. Segal, D. Landau and E. Sheiner. Long term hematological morbidity in children ollowing assisted reproductive technology (ART) pregnancies compared to spontaneous pregnancies. 2017. 216 (1 Supplement 1): S350.
9. Zhang X.J.; Lv Q.; Min L.H.; Cao X.H.; Li X.J. Lv Q. Effect of Developmental Stage of Embryos at Freezing on Live Birth Outcomes After Frozen EmbryoTransfer. Cryoletters. 2017. 38(5):399-406.
10. N. Kultursay,​ O. A. Koroglu,​ O. Uygur,​ D. Terek,​ S. Tanriverdi,​ M. Akisu and M. Yalaz. Improved neonatal prognosis following restriction in the number of transferred embryos in assisted reproduction - single center yearly comparison from Turkey. 2015. 42 (4): 442-7.
11. J. D. Kapfhamer, S. Palaniappan, K. M. Summers, A. Mancuso, G. Ryan and D. Shah. Mean gestational sac diameter to crown-rump length difference as a marker of first trimester pregnancy loss after in vitro fertilization. 2016. 106: e342-e343.
12. S. Mukantabana. [Infertility in Rwanda]. 1985. (2): 21-7.
13. Stern JE, Liu CL, Cabal HJ, Gopal D, Harvey L, Coddington CC, Missmer SA, Diop H. Hospitalization before pregnancy in infertile women treated with ART, non-ART, or no fertility therapy compared with fertile women. Human Reproduction 2018. 33: 489-490.
14. R. Mendoza, S. Perez, M. J. De Los Santos, Z. Larreategui, A. Exposito, M. V. Aparicio, L. Martinez Indart and R. Matorras. Is embryo quality associated with an increase in congenital malformations in the newborns?. 2011. 26: i274.
15. J. K. Min,​ M. Gysler,​ A. P. Cheung,​ G. J. Goodrow,​ E. Hughes,​ J. Min,​ V. Senikas,​ D. Young,​ A. Cheung,​ S. Sierra,​ B. Carranza-Mamane,​ A. Case,​ C. Dwyer,​ J. Graham,​ J. Havelock,​ R. Hemmings,​ F. Lee,​ K. Liu,​ T. Vause and B. Chee-Man Wong. Elective Single Embryo Transfer Following In Vitro Fertilization. 2010. 32 (4): 363-377.
16. G. Oron, T. Sokal-Arnon, A. Zeadna, W. Y. Son, H. Holzer and T. Tulandi. Effects of embryo quality on pregnancy and perinatal outcome. 2013. 28: i287.
17. Johnson K.M.; Hacker M.R.; Resetkova N.; O'Brien B.; Modest A.M. The risk of ischemic placental disease in fresh and frozen embryo transfer cycles. American Journal of Obstetrics and Gynecology. 2018. 1 Supplement 1. 218. S448.
18. S. M. Nelson and D. A. Lawlor. Impact of number of embryos transferred on live birth and adverse perinatal outcomes: A prospective study of 124,148 IVF cycles. 2011. 26: i338.
19. G. Augustin, P. Matosevic, E. Kinda, T. Kekez, M. Majerovic and H. Silovski. Are additional perioperative considerations needed in emergency laparoscopy in in vitro fertilization pregnancy. 2014. 28: S84.
20. E. G. Hurley and E. De Franco. Influence of paternal age on perinatal outcomes in pregnancies achieved with assisted reproductive technologies. 2016. 106: e166.
21. S. Mourad, C. Farquhar and L. Sadler. Perinatal death after fertility treatment; a 7-year national cohort study in New Zealand on contributory factors and potential avoidability. 2016. 31: i370.
22. V. Tomek, J. Gilik, J. Skovranek and J. Janousek. Prenatal detection of congenital heart defects after in vitro fertilization. 2014. 24: S86-S87.
23. M. Davies, A. Rumbold, J. Marino, K. Willson, V. Moore and L. Giles. Sex variation in perinatal outcomes by infertility treatment modality in a population cohort. 2013. 28: i285.
24. X. Zhong,​ J. Liu,​ Q. Cui,​ S. Liang,​ Y. Lin,​ H. Liu and Q. Zeng. Effect of parental physiological conditions and assisted reproductive technologies on the pregnancy and birth outcomes in infertile patients. 2017. 8 (11): 18409-18416.
25. Wang C, Feng G, Zhang B, Zhou H, Shu J, Lin R, Chen H, Wu Z. Effect of different artificial shrinkage methods, when applied before blastocyst vitrification, on perinatal outcomes. Reproductive Biology and Endocrinology. 2017. 15(1):32.
26. W. Wurfel,​ H. Haas-Andela,​ G. Krusmann,​ M. Rothenaicher,​ P. Hirsch,​ H. K. Kwapisz,​ J. Haas,​ I. Hogemann and K. Fiedler. Prenatal diagnosis by amniocentesis in 82 pregnancies after in vitro fertilization. 1992. 44 (1): 47-52.
27. N. Yerebasmaz,​ B. Demir,​ I. Kahyaoglu,​ A. Ocal,​ I. Kaplanoglu and L. Mollamahmutoglu. Does male factor infertility affect intracytoplasmic sperm injection pregnancy results?. 2017. 4 (2): 85-89.
28. J. F. Kawwass,​ S. Crawford,​ D. M. Kissin,​ D. R. Session,​ S. Boulet and D. J. Jamieson. Tubal factor infertility and perinatal risk after assisted reproductive technology. 2013. 121 (6): 1263-71.
29. J. E. Stern,​ B. Luke,​ M. D. Hornstein,​ H. Cabral,​ D. Gopal,​ H. Diop and M. Kotelchuck. The effect of father's age in fertile,​ subfertile,​ and assisted reproductive technology pregnancies: a population based cohort study. 2014. 31 (11): 1437-44.
30. H. Kaya,​ M. Karci,​ O. Ozkaya and M. Sezik. Relationship between the timing of hysterosalpingography before gamete intrafallopian transfer and the subsequent fertility outcome. 2004. 30 (6): 448-53.
31. B. O. Asvold,​ L. J. Vatten,​ T. G. Tanbo and A. Eskild. Concentrations of human chorionic gonadotrophin in very early pregnancy and subsequent pre-eclampsia: A cohort study. 2014. 29 (6): 1153-1160.
32. B. V. Rossi,​ G. Chang,​ K. F. Berry,​ M. D. Hornstein and S. A. Missmer. In vitro fertilization outcomes and alcohol consumption in at-risk drinkers: the effects of a randomized intervention. 2013. 22 (5): 481-5.
33. D. L. Zander-Fox,​ R. Henshaw,​ H. Hamilton and M. Lane. Does obesity really matter? The impact of BMI on embryo quality and pregnancy outcomes after IVF in women aged <=38 years. 2012. 52 (3): 270-6.
34. S. Tandulwadkar, P. Lodha and V. Kharb. Congenital malformations and assisted reproductive technique: Where is assisted reproductive technique taking us?. 2012. 5 (3): 244-7.
35. A. S. Martin, Y. Zhang, S. Crawford, S. L. Boulet, P. McKane, D. M. Kissin, D. J. Jamieson and C. States Monitoring Assisted Reproductive Technology. Antenatal Hospitalizations Among Pregnancies Conceived With and Without Assisted Reproductive Technology. 2016. 127 (5): 941-50.
36. I. Netchine, S. Rossignol, S. Azzi and Y. Le Bouc. Epigenetic anomalies in childhood growth disorders. 2013. 71: 65-73.
37. E. Jauniaux, I. Ben-Ami and R. Maymon. Do assisted-reproduction twin pregnancies require additional antenatal care?. 2013. 26 (2): 107-19.
38. N. R. Vulliemoz, E. McVeigh and J. Kurinczuk. In vitro fertilisation: perinatal risks and early childhood outcomes. 2012. 15 (2): 62-8.
39. A. A. Pacey. Fertility issues in survivors from adolescent cancers. 2007. 33 (7): 646-55.
40. E. Lieberman, E. S. Ginsburg and C. Racowsky. Rate of cell division and weight of neonates following IVF. 2006. 12 (3): 315-21.
41. J. Salat-Baroux, J. Aknin, J. M. Antoine, S. Alvarez, D. Cornet, M. Plachot and J. Mandelbaum. Is there an indication for embryo reduction?. 1992. 7 Suppl 1: 67-72.
42. R. S. Legro. Introduction: Evidence-based in vitro fertilization treatment of fresh versus frozen embryo transfer: peeling away the layers of the onion. 2016. 106 (2): 239-240.
43. M. L. Macer, J. Barritt, M. W. Surrey, H. Danzer, S. Ghadir, W. Chang and M. D. Pisarska. Pregnancy outcomes following single versus double euploid embryo transfer. 2017. 107 (3 Supplement 1): e28-e29.
44. K.S. Richter G.L. Mottla, B. Kaplan, B. Hayward, M.C. Mahony. Weighing the impact of ESET versus DET on live birth rates and associated multiple rates for 38,020 day 5/6 transfers in a large real-world database. 107 (3 Supplement 1): e15-e16.
45. S. Santos-Ribeiro, V. Costa Ribeiro, N. De Munck, P. Drakopoulos, P. P. N, V. Schutyser, G. Verheyen, H. Tournaye and C. Blockeel. Why do we still measure endometrial thickness and what is its effect on live birth rates?. 2016. 31: i48-i49.
46. A. Harlev, T. Wainstock, A. Walfisch, I. Segal, D. Landau and E. Sheiner. Long term endocrine disorders in children following assisted reproductive technology (ART) pregnancies compared with spontaneous pregnancies. 2017. 216 (1 Supplement 1): S349.
47. S. Chaabane, O. Sheehy, P. Monnier, F. Bissonnette, J. M. Trasler, W. Fraser and A. Berard. Ovarian Stimulators, Intrauterine Insemination, and Assisted Reproductive Technologies Use and the Risk of Major Congenital Malformations-The AtRISK Study. 2016. 107 (3): 136-147.
48. A. Coates, A. Kung, E. Mounts, J. S. Hesla, B. J. Bankowski, E. Barbieri, B. Ata, J. Cohen and S. Munne. Transfer fresh or vitrify after blastocyst biopsy? Results of an RCT. 2016. 106: e58-e59-=.
49. V. Fesslova, C. Boschetto and J. Brankovic. Congenital heart disease in fetuses conceived after assisted reproductive technology. 2015. 25: S91.
50. J. Rodriguez-Purata, J. Gingold, M. C. Whitehouse, J. A. Lee and A. B. Copperman. Do elevated TSH levels predict early pregnancy loss in art patients?. 2015. 104 (3 SUPPL. 1): e347.
51. S. Ates, T. Shaulov and M. H. Dahan. The effect of small uterine dimensions on pregnancy and live birth rates after embryo transfer. 2015. 30: i30-i31.
52. J. F. Kawwass, M. Monsour, S. Crawford, D. M. Kissin, D. R. Session, A. D. Kulkarni and D. J. Jamieson. Trends and outcomes for donor oocyte cycles in the United States, 2000-2010. 2014. 69 (4): 189-191.
53. C. D. Dirksen, M. M. J. Van Heesch, J. C. M. Dumoulin, M. Van Der Hoeven, C. E. M. Van Beijsterveldt, G. J. Bonsel, R. H. M. Dykgraaf, J. B. Van Goudoever, C. Koopman-Esseboom, W. L. D. M. Nelen, K. Steiner, P. Tamminga, N. Tonch, P. Van Zonneveld and J. L. H. Evers. Hospital costs from birth up to age five of multiples and singletons born by in vitro fertilization: A longitudinal 5-year follow-up study. 2014. 29: i277.
54. H. C. Darby. Reducing multiple births: The UK experience. 2012. 27.
55. D. Hvidtjorn, J. Grove, D. Schendel, L. Schieve, B. Jacobsson, E. Ernst, P. Uldall and P. Thorsen. Assisted conception leads to more children with cerebral palsy. 2009. 51: 16-17.
56. K. Harrild, C. Bergh, M. Davies, D. De Neubourg, J. C. M. Dumoulin, J. Gerris, J. A. M. Kremer, H. Martikainen, B. W. Mol, R. J. Norman, T. Kjelberg, A. P. A. Van Montfoort, A. Van Peperstraten, E. Van Royen and S. Bhattacharya. Clinical effectiveness of elective single versus double embryo transfer: Results from an individual patient data meta-analysis of randomised trials. 2009. 24: i77.
57. S. Senoz, A. Ben-Chetrit and R. F. Casper. An IVF fallacy: Multiple pregnancy risk is lower for older women. 1997. 14 (4): 192-198.
58. A. Thurin, J. Hausken, T. Hillensj”, B. Jablonowska, A. Pinborg, A. Strandell and C. Bergh. Elective single-embryo transfer versus double-embryo transfer in in vitro fertilization. 2004. 351 (23): 2392-2402.
59. Mastrolia SA, Baumfeld Y, Hershkovitz R, Loverro G, Di Naro E, Yohai D, Schwarzman P, Weintraub AY. Bicornuate uterus is an independent risk factor for cervical os insufficiency: a retrospective population based cohort study. The Journal of Maternal-Fetal & Neonatal Medicine. 2017. 30(22):2705-10.
60. Moini A, Mohammadi Yeganeh L, Shiva M, Ahmadieh M, Salman Yazdi R, Hasani F, Bagheri Lankarani N, Sanati A. Bacterial vaginosis and the risk of early miscarriage in women undergoing intracytoplasmic sperm injection cycles: a prospective cohort study. Human fertility. 2018. 21(4):263-8.
61. Chambers AE, Fairbairn C, Gaudoin M, Mills W, Woo I, Pandian R, Stanczyk FZ, Chung K, Banerjee S. Soluble LH-HCG receptor and oestradiol as predictors of pregnancy and live birth in IVF. Reproductive biomedicine online. 2019. 38(2):159-68.
62. Goldsmith S, Mcintyre S, Badawi N, Hansen M. Cerebral palsy after assisted reproductive technology: a cohort study. Developmental medicine & child neurology. 2018. 60(1):73-80.
63. Liberman RF, Getz KD, Heinke D, Luke B, Stern JE, Declercq ER, Chen X, Lin AE, Anderka M. Assisted reproductive technology and birth defects: effects of subfertility and multiple births. Birth defects research. 2017. 109(14):1144-53.
64. Zegers-Hochschild F, Schwarze JE, Crosby JA, Musri C, Urbina MT. Assisted reproductive techniques in Latin America: The Latin American Registry, 2014. JBRA assisted reproduction. 2017. 21(3):164-175
65. Bardin R, Oron G, Levy Y, Sapir O, Meizner I, Fisch B, Wiznitzer A, Hadar E. First-trimester inter-and intrafetal size discrepancies in bichorionic twins conceived by in vitro fertilization: can it predict pregnancy outcome?. Fertility and sterility. 2017. 108(2):296-301.
66. Allvee K, Rahu M, Haldre K, Karro H, Rahu K. Quality of IVF status registration in the Estonian Medical Birth Registry: a national record linkage study. Reproductive health. 2018. 15(1):133.
67. Boyle PC, de Groot T, Andralojc KM, Parnell TA. Healthy Singleton Pregnancies From Restorative Reproductive Medicine (RRM) After Failed IVF. Frontiers in medicine. 2018. 5 (101648047): 210
68. Cheng R, Ma Y, Nie Y, Qiao X, Yang Z, Zeng R, Xu L. Chromosomal polymorphisms are associated with female infertility and adverse reproductive outcomes after infertility treatment: a 7-year retrospective study. Reproductive biomedicine online. 2017. 35(1):72-80.
69. Wainstock T, Walfisch A, Shoham-Vardi I, Segal I, Harlev A, Sergienko R, Sheiner E. Assisted reproductive technology and pediatric neoplasms-results of a population based cohort of up to 18 years. American Journal of Obstetrics and Gynecology. 2017. 216(1).
70. Li H, Zhu HL, Chang XH, Li Y, Wang Y, Guan J, Cui H. Effects of previous laparoscopic surgical diagnosis of endometriosis on pregnancy outcomes. Chinese medical journal. 2017. 130(4):428.
71. Wainstock T, Walfisch A, Shoham-Vardi I, Segal I, Harlev A, Sergienko R, Landau D, Sheiner E. Fertility treatments and pediatric neoplasms of the offspring: results of a population-based cohort with a median follow-up of 10 years. American journal of obstetrics and gynecology. 2017. 216(3):314-e1.
72. Harlev A, Wainstock T, Walfisch A, Segal I, Landau D, Sheiner E. 727: Long-term pediatric gastrointestinal morbidity of singletons conceived following fertility treatments. American Journal of Obstetrics & Gynecology. 2018. 218(1):S436-7.
73. Harlev A, Wainstockb T, Walfischa A, Segalc I, Landaud D, Levina S, Levitasa E, Sheinera E. Long-term neurological morbidity in children following in-vitro fertilization and ovulation induction pregnancies. Human Reproduction 2017. 32: 493-493.
74. Barua S, Hng TM, Smith H, Bradford J, McLean M. Ovulatory disorders are an independent risk factor for pregnancy complications in women receiving assisted reproduction treatments. Australian and New Zealand Journal of Obstetrics and Gynaecology. 2017. (3):286-93
75. R. Moffat, N. Bergsma, G. Sartorius, A. Raggi, U. Guth and C. De Geyter. Does prior hysteroscopy affect pregnancy outcome in primigravid infertile women?. 2014. 211 (2): 130.e1-6.
76. O. Tan and B. R. Carr. The impact of bariatric surgery on obesity-related infertility and in vitro fertilization outcomes. 2012. 30 (6): 517-28.
77. S. L. F. Gustin, G. Mukherjee, V. L. Baker, L. M. Westphal, A. A. Milki and R. B. Lathi. Early pregnancy testosterone after ovarian stimulation and pregnancy outcome. 2012. 97 (1): 23-7.e1.
78. J. L. Zhu, D. Hvidtjorn, O. Basso, C. Obel, P. Thorsen, P. Uldall and J. Olsen. Parental infertility and cerebral palsy in children. 2010. 25 (12): 3142-5.
79. A. J. B. Kallen, O. O. Finnstrom, A. P. Lindam, E. M. E. Nilsson, K.-G. Nygren and P. M. O. Olausson. Cerebral palsy in children born after in vitro fertilization. Is the risk decreasing?. 2010. 14 (6): 526-30.
80. L.-M. Sun, M. C. Walker, H.-L. Cao, Q. Yang, T. Duan and J. C. P. Kingdom. Assisted reproductive technology and placenta-mediated adverse pregnancy outcomes. 2009. 114 (4): 818-24.
81. M. M. Aboulghar, M. A. Aboulghar, L. Mourad, G. I. Serour and R. T. Mansour. Ultrasound cervical measurement and prediction of spontaneous preterm birth in ICSI pregnancies: a prospective controlled study. 2009. 18 (2): 296-300.
82. J. L. Zhu, O. Basso, C. Obel, D. Hvidtjorn and J. Olsen. Infertility, infertility treatment and psychomotor development: the Danish National Birth Cohort. 2009. 23 (2): 98-106.
83. L. Wilkins-Haug, A. Porter, P. Hawley and C. B. Benson. Isolated fetal omphalocele, Beckwith-Wiedemann syndrome, and assisted reproductive technologies. 2009. 85 (1): 58-6
84. I. N. Damgaard, T. K. Jensen, G. Nordic Cryptorchidism Study, J. H. Petersen, N. E. Skakkebaek, J. Toppari and K. M. Main. Risk factors for congenital cryptorchidism in a prospective birth cohort study. 2008. 3 (8): e3051.
85. P. Merlob, O. Sapir, J. Sulkes and B. Fisch. The prevalence of major congenital malformations during two periods of time, 1986-1994 and 1995-2002 in newborns conceived by assisted reproduction technology. 2005. 48 (1): 5-11.
86. B. Rizk,​ P. Doyle,​ S. L. Tan,​ P. Rainsbury,​ J. Betts,​ P. Brinsden and R. Edwards. Perinatal outcome and congenital malformations in in-vitro fertilization babies from the Bourn-Hallam group. 1991. 6 (9): 1259-64.
87. J. Cleary-Goldman, J. E. Pena, M. H. Thornton, 2nd, J. N. Robinson, M. E. D'Alton and M. V. Sauer. Obstetric outcomes of human immunodeficiency virus-1-serodiscordant couples following in vitro fertilization with intracytoplasmic sperm injection. 2003. 20 (6): 305-11.
88. N. Linder, O. Haskin, O. Levit, G. Klinger, T. Prince, N. Naor, P. Turner, B. Karmazyn and L. Sirota. Risk factors for intraventricular hemorrhage in very low birth weight premature infants: a retrospective case-control study. 2003. 111 (5 Pt 1): e590-5.
89. M. Gil-Salom, J. Romero, Y. Minguez, M. D. Molero, J. Remohi and A. Pellicer. Testicular sperm extraction and intracytoplasmic sperm injection: a chance of fertility in nonobstructive azoospermia. 1998. 160 (6 Pt 1): 2063-7.
90. H. Timur, N. Yilmaz, M. Cinar, H. A. Inal, N. Hancerliogullari, M. Alkan, M. Kaba and D. Uygur. The association between first trimester uterine artery Doppler velocimetry indices and adverse perinatal outcomes in IVF cycles. 2016. 31: i172.
91. T. Simoes, A. Queiros, A. Marujo, S. Valdoleiros and A. Coelho. Double embryo transfer (DET): Just for women taller than 1.65 m?. 2016. 31: i73-i74.
92. G. Sisti, T. T. Kanninen, M. Di Tommaso, S. S. Witkin and S. D. Spandorfer. Autophagy induction by sera from women undergoing an in vitro fertilization cycle varies with subsequent outcome. 2016. 117: 1-3.
93. J. DeAnna, O. Abuzeid, I. Fahmi, I. Vettraino, F. G. Rocha and M. Abuzeid. The place for prophylactic cerclage in the infertile patient with established cervical incompetence. 2016. 127: 36S.
94. M. Cinar, H. Timur and N. Yilmaz. The association between first trimester uterine artery Doppler velocimetry indices and adverse perinatal outcomes in IVF cycles. 2016. 17: S263.
95. H. T. N. Yen, B. T. P. Loan, H. N. A. Vu and D. Q. Vinh. The prevalence of asymptomatic bacteriuria in women conceived through assisted reproductive technologies. 2015. 41: 119.
96. N. Perez Martinez, P. Suarez-Gil, S. Garcia, D. Llaneza-Suarez, S. J. L. Sanchez and P. Llaneza. Perinatal results after IVF/ICSI. A prospective study. 2015. 30: i183-i184.
97. K. Kong, A. Macaldowie and M. Chapman. Changes in caesarean section rates in ART pregnancies over the last 10 years. 2015. 122: 190
98. H. E. Shawki. The impact of in situ methotrexate injection after transvaginal ultrasound-guided aspiration of ovarian endometriomas on ovarian response and reproductive outcomes during IVF-cycles. 2012. 17 (2): 82-88.
99. M. S. Kamath, A. M. Mangalaraj, K. Muthukumar, R. Cullinan, T. K. Aleyamma and K. George. Blastocyst cryopreservation using solid surface vitrification: A preliminary study. 2011. 4 (3): 114-120
100. N. Okumura, N. Kuji, A. Kishimi, H. Nishio, Y. Mochimaru, K. Minegishi, K. Miyakoshi, T. Fujii, M. Tanaka, D. Aoki, Y. Yoshimura and K. Hasegawa. Fecundity and perinatological outcome after abdominal radical trachelectomy in 80 women with early-stage cervical cancer. 2010. 25: i268-i269.
101. A. E. Semprini, L. H. Hollander, A. Vucetich and C. Gilling-Smith. Infertility treatment for HIV-positive women. 2008. 4 (4): 369-382.
102. R. Romero, J. Espinoza and M. Mazor. Can endometrial infection/inflammation explain implantation failure, spontaneous abortion, and preterm birth after in vitro fertilization?. 2004. 82 (4): 799-804.
103. E. Geva, A. Amit, L. Lerner-Geva, Y. Yaron, Y. Daniel, T. Schwartz, F. Azem, I. Yovel and J. B. Lessing. Prednisone and aspirin improve pregnancy rate in patients with reproductive failure and autoimmune antibodies: A prospective study. 2000. 43 (1): 36-40.
104. Ma NZ, Chen L, Hu LL, Dai W, Bu ZQ, Sun YP. The influence of male age on treatment outcomes and neonatal birthweight following assisted reproduction technology involving intracytoplasmic sperm injection (ICSI) cycles. Andrologia. 2018. 50(1): e12826.
105. Mayama M.; Nomura E.; Yamada T.; Takeda T.; Uno K.; Tano S.; Ukai M.; Suzuki T.; Kishigami Y.; Oguchi H.The effect of pre-pregnancy body mass index on perinatal outcomes in normal weight women. Journal of Perinatal Medicine. 2017. 45 Supplement 2: 554.
106. Timur H, Çinar M, Hançerlioğullari N, Alkan M, İnal HA, Uygur D, Yilmaz N. Associations between first-trimester uterine artery Doppler velocimetry indices and adverse perinatal outcomes in women conceiving via in vitro fertilization. The Journal of Maternal-Fetal & Neonatal Medicine. 2017. 30(6):684-8.
107. Fuchs F, Monet B, Ducruet T, Chaillet N, Audibert F. Effect of maternal age on the risk of preterm birth: A large cohort study. PloS one. 2018. 13(1): e0191002.

## Did not evaluate IVF/ICSI, or did not showed type of ART separately

1. S. S. Malchau,​ A. Loft,​ A.-K. A. Henningsen,​ A. Nyboe Andersen and A. Pinborg. Perinatal outcomes in 6,​338 singletons born after intrauterine insemination in Denmark,​ 2007 to 2012: the influence of ovarian stimulation. 2014. 102 (4): 1110-1116.e2.
2. Yılmaz NK, Sargın A, Erkılınç S, Özer İ, Engin-Üstün Y. Does ovulation induction and intrauterine insemination affect perinatal outcomes in singletons?. The Journal of Maternal-Fetal & Neonatal Medicine. 2018. 31(1):14-7.
3. C. Messerlian,​ S. L. Tan,​ R. Platt,​ R. Gagnon and O. Basso. Low technology assisted reproduction and preterm birth. 2013. 177: S116.
4. S. Nuojua-Huttunen,​ M. Gissler,​ H. Martikainen and L. Tuomivaara. Obstetric and perinatal outcome of pregnancies after intrauterine insemination. 1999. 14 (8): 2110-5.
5. Hsu JY, James KE, Bormann CL, Donahoe PK, Pépin D, Sabatini ME. Müllerian-Inhibiting Substance/Anti-Müllerian Hormone as a Predictor of Preterm Birth in Polycystic Ovary Syndrome. The Journal of Clinical Endocrinology & Metabolism. 2018. 103(11):4187-96.
6. Chen L, Zhu L, Cai C, Yan G, Sun H. Clinical and neonatal outcomes of intrauterine insemination with frozen donor sperm. Systems biology in reproductive medicine. 2018. 64(4):240-5.
7. Dimitriadis, G. Christou, J. C. Petrozza and I. Souter. The impact of peak serum estradiol (e2) levels on birth weights(BW)and adverse pregnancy outcomes (apo) following gonadotropin-induction/intrauterine insemination (GN/IUI) cycles. 2016. 106: e197.
8. M. Sanam. Cause of infertility and response to ovulation induction drugs - A hospital based study. 2008. 20 (1): 21-24.
9. G. D. Royster, J. M. Csokmay, B. J. Yauger, R. J. Chason, A. H. DeCherney, C. McTear and M. J. Hill. Elevated estradiol levels (> 90th percentile) at the time of HCG trigger during controlled ovarian hyperstimulation increases obstetrical risks later in pregnancy. 2015. 22: 225A.
10. S. Barua,​ T.-M. Hng,​ H. Smith,​ J. Bradford and M. McLean. Ovulatory disorders are an independent risk factor for pregnancy complications in women receiving assisted reproduction treatments. 2017. 57 (3): 286-293Z. Sabeti Rad, B. Friberg, E. Henic, L. Rylander, O. Stahl, B. Kallen and G. Lingman. Deliveries After Malignant Disease Before Pregnancy: Maternal Characteristics, Pregnancy, and Delivery Complications. 2016. 5 (3): 240-7.
11. S. Koshida, T. Ono, S. Tsuji, T. Murakami and K. Takahashi. Perinatal Backgrounds and NICU Bed Occupancy of Multiple-Birth Infants in Japan. 2016. 238 (3): 261-5.
12. C. Messerlian, R. W. Platt, B. Ata, S.-L. Tan and O. Basso. Do the causes of infertility play a direct role in the aetiology of preterm birth? 2015. 29 (2): 101-12.
13. C. Messerlian, R. W. Platt, S.-L. Tan, R. Gagnon and O. Basso. Low-technology assisted reproduction and the risk of preterm birth in a hospital-based cohort. 2015. 103 (1): 81-8.e2.
14. N. Kultursay, M. Yalaz, O. A. Koroglu and M. A. R. N. S. Group. Neonatal outcome following new assisted reproductive technology regulations in Turkey - a nationwide multicenter point prevalence study. 2015. 28 (2): 204-9.
15. F. W. Lung, B. C. Shu, T. L. Chiang and S. J. Lin. Twin-singleton influence on infant development: a national birth cohort study. 2009. 35 (3): 409-18.
16. D. V. D'Angelo, N. Whitehead, K. Helms, W. Barfield and I. B. Ahluwalia. Birth outcomes of intended pregnancies among women who used assisted reproductive technology, ovulation stimulation, or no treatment. 2011. 96 (2): 314-320.e2.
17. R. Bukowski, G. C. S. Smith, F. D. Malone, R. H. Ball, D. A. Nyberg, C. H. Comstock, G. D. V. Hankins, R. L. Berkowitz, S. J. Gross, L. Dugoff, S. D. Craigo, I. E. Timor-Tritsch, S. R. Carr, H. M. Wolfe, M. E. D'Alton and F. R. Consortium. Fetal growth in early pregnancy and risk of delivering low birth weight infant: prospective cohort study. 2007. 334 (7598): 836.
18. V. M. Allen, R. D. Wilson, A. Cheung, O. Genetics Committee of the Society of, C. Gynaecologists of, O. Reproductive Endocrinology Infertility Committee of the Society of and C. Gynaecologists of. Pregnancy outcomes after assisted reproductive technology. 2006. 28 (3): 220-50.
19. B. J. Van Voorhis. Outcomes from assisted reproductive technology. 2006. 107 (1): 183-200.
20. T. Shevell, F. D. Malone, J. Vidaver, T. F. Porter, D. A. Luthy, C. H. Comstock, G. D. Hankins, K. Eddleman, S. Dolan, L. Dugoff, S. Craigo, I. E. Timor, S. R. Carr, H. M. Wolfe, D. W. Bianchi and M. E. D'Alton. Assisted reproductive technology and pregnancy outcome. 2005. 106 (5 Pt 1): 1039-45.
21. M. A. Aboulghar. Perinatal complications of assisted reproduction. 2005. 46 (5): 751-8.
22. M. Filicori, G. E. Cognigni, E. Gamberini, E. Troilo, L. Parmegiani and S. Bernardi. Impact of medically assisted fertility on preterm birth. 2005. 112 Suppl 1: 113-7.
23. N. S. Green. Risks of birth defects and other adverse outcomes associated with assisted reproductive technology. 2004. 114 (1): 256-9.
24. G. A. Hill, S. Bryan, C. M. Herbert, 3rd, D. M. Shah and A. C. Wentz. Complications of pregnancy in infertile couples: routine treatment versus assisted reproduction. 1990. 75 (5): 790-4.
25. K. Ozgur, H. Bulut, M. Berkkanoglu and K. Coetzee. Optimism in live birth rates of unicornuate uterus patients. 2016. 31: i173.
26. K. A. Whitton, A. W. Shand, A. Pasfield, N. Nassar, M. McShane, X. Han and A. Henry. Anti-mullerian hormone: A predictor of adverse pregnancy outcome?. 2013. 49: 82.

**Oocyte/egg donation/gestational surrogate/donor sperm**

1. A. Richardson, J. Garner, M. Parisaei, P. Gopaldas and A. Shah. Maternal and neonatal outcomes of 77 ovum donation pregnancies at Homerton University Hospital, London. 2016. 123: 50.
2. H. S. Hipp, S. L. Boulet, S. Boulet, J. Chang, S. Crawford, D. J. Jamieson, J. F. Kawwass, D. M. Kissin, A. Kulkarni, M. Sunderam and Y. Zhang. Embryo donation: national trends and outcomes, 2000 through 2013. 2016. 215 (6): 747.
3. N. E. Reame and P. J. Parker. Surrogate pregnancy: clinical features of forty-four cases. 1990. 162 (5): 1220-5.
4. E. Elenis, A. Skoog-Svanberg and G. Sydsjo. Adverse obstetrical and neonatal outcomes in pregnancies resulting from oocyte donation. 2015. 30: i302-i303. (Sweden)
5. E. Elenis,​ G. Sydsjo,​ A. Skalkidou,​ C. Lampic and A. S. Svanberg. Neonatal outcomes in pregnancies resulting from oocyte donation: a cohort study in Sweden. 2016. 16 (1): 170. (Sweden)
6. B. Alvaro Mercadal, R. Imbert, I. Demeestere, Y. Englert and A. Delbaere. Pregnancy outcome after oocyte donation in patients with Turner's syndrome and partial X monosomy. 2011. 26 (8).
7. D. Bodri, V. Vernaeve, F. Figueras, R. Vidal, J. J. Guillen and O. Coll. Oocyte donation in patients with Turner's syndrome: a successful technique but with an accompanying high risk of hypertensive disorders during pregnancy. 2006. 21 (3): 829-32.
8. P. Fenichel, H. Letur, N. Chevalier, D. Lelannou, J. Ohl, D. Cornet, C. Chalas-Boissonnas, S. Jonard-Catteau, T. H. Greck-Chassain and I. Cedrin-Durnerin. Materno-fetal cardiovascular complications after oocyte donation in turner syndrome: A very high-risk pregnancy. 2010. 25: i22.
9. E. Clua Obrado, D. Rodriguez Barredo, L. Latre Navarro, A. Vazquez Rodriguez, P. N. Barri Rague, B. Coroleu Lletget and R. Tur Padro. Obstetric and perinatal follow-up in an oocyte donation program: Why should we transfer only one embryo. 2013. 28: i286-i287.
10. V. Soderstrom-Anttila. Pregnancy outcome after oocyte donation in women with Turner syndrome-what are the risks?. 2014. 29: i55.
11. H. Kort, J. Choi, P. Dutta, M. Guarnaccia, J. Cleary-Goldman and M. V. Sauer. Defining perinatal risks in donor-egg IVF (D-IVF) pregnancies: Analysis of 100 consecutive women 50 years and above. 2009. 92 (3 SUPPL. 1): S156.
12. R. Fritz, S. K. Jindal, B. Yu, M. G. Vega and E. Buyuk. Does donor sperm affect birth weight (BW), preterm birth (PB), and miscarriage rates in fresh autologous in vitro fertilization (IVF) cycles? Analysis of 46,061 cycles reported to sart. 2017. 107 (3 Supplement 1): e30.
13. Bartal MF, Sibai BM, Bart Y, Shina A, Mazaki-Tovi S, Eisen IS, Hendler I, Baum M, Schiff E. The impact of sperm and egg donation on the risk of pregnancy complications. American journal of perinatology. 2019 Jan;36(02):205-11Gerkowicz SA, Crawford SB, Hipp HS, Boulet SL, Kissin DM, Kawwass JF. Assisted reproductive technology with donor sperm: national trends and perinatal outcomes. American journal of obstetrics and gynecology. 2018. 218(4):421-e1. Donor sperm
14. S. Talebi Chahvar, V. Biondini, S. Battistoni, S. Giannubilo and A. L. Tranquilli. Obstetric outcomes in oocyte donor pregnancy. 2011. 26: i235.
15. L. Guilbaud, E. Studer, P. Santulli, V. Gayet, F. Goffinet and C. Le Ray. Obstetric and perinatal outcomes of twin gestations after oocyte donation. 2015. 212 (1 SUPPL. 1): S401.
16. L. Sekhon, R. Gerber, A. Rebarber, D. Saltzman, C. Klauser, S. Gupta and N. Fox. The effect of oocyte donation on pregnancy outcomes in IVF twin gestations. 2014. 210 (1 SUPPL. 1): S289
17. Eaton JL, Truong T, Li Y, Polotsky AJ. Prevalence of a healthy birth following in vitro fertilization with fresh versus cryopreserved donor oocytes in the united states: a 2012-2014 national study. Fertility and Sterility. 2017. 108(3):e77-8.
18. M. V. Sauer, R. J. Paulson and R. A. Lobo. Pregnancy in women 50 or more years of age: outcomes of 22 consecutively established pregnancies from oocyte donation. 1995. 64 (1): 111-5
19. Vega MG, Zaghi S, Buyuk E, Jindal SK, Yu B. Perinatal outcomes in autologous versus donor egg recipient (DER) cycles in older patients: analysis of 156,873 cycles reported to SART cors. Fertility and Sterility. 2017. 108(3):e71-2.
20. Y. B. Jeve, N. Potdar and M. Khare. Does donor oocyte conception act as an independent risk factor for pregnancy complications?. 2015. 30: i30.
21. S. Talebi Chahvar, V. Biondini and A. L. Tranquilli. The risk of adverse perinatal outcomes in oocyte donor pregnancies. 2010. 23: 365.
22. S. Lin, T. Singer, E. Milbank, N. Ward, D. Skupski and A. Grunebaum. Pregnancy outcomes in multiparous women 40 years and older with spontaneous, IVF autologous oocyte and donor oocyte singleton pregnancies. 2012. 19 (3 SUPPL. 1): 285A.
23. H. Check, S. Patel, J. K. Choe and D. Brasile. The effect of donated oocytes or embryos on preterm delivery and birth weight in women with multiple gestation. 2010. 93 (5 SUPPL 1): S10-S11.
24. Dang. To compare obstetric and perinatal outcomes in oocyte recipients and in vitro fertilisation pregnancies. 2014. 121: 84.
25. Klenov V, Boulet S, Mejia R, Kissin DM, Munch E, Mancuso A, Van Voorhis B. Live birth and multiple birth rates in donor oocyte cycles using elective single embryo transfer vs double embryo transfer in United States in-vitro-fertilization clinics. Fertility and Sterility. 2017. 108(3):e98.
26. Takahashi N.; Miyake T.; Nakamura H.; Kakigano A.; Takiuchi T.; Matsuzaki S.; Mimura K.; Kumasawa K.; Tomimatsu T.; Endo M.; Kimura T. . Obstetric and neonatal complications in pregnancies achieved by oocyte donation in our institution.Journal of Obstetrics and Gynaecology Research. 2018. 44(8):1634
27. Szymusik I, Kosinski P, Kosinska-Kaczynska K, Warzecha D, Karwacka A, Kaczynski B, Wielgos M. The first trimester aneuploidy biochemical markers in IVF/ICSI patients have no additional benefit compared to spontaneous conceptions in the prediction of pregnancy complications. Journal of perinatal medicine. 2018. 46(9):953-9.
28. R. Juneau, M. D. Werner, J. Franasiak, K. A. Green, S. Lubitz and R. T. Scott. Conception period does not improve oocyte quality in donor IVF. 2016. 127: 62S.
29. Capelouto SM, Nagy ZP, Spencer JB, Shapiro D, Hipp HS. Effects of Male Partner Age on Pregnancy and Obstetric Outcomes in Frozen Oocyte Donor Cycles [40n]. Obstetrics & Gynecology. 2018. 131:162S-3S.
30. S. N. Lin, T. Singer, E. Milbank, M. Biewald and A. Grunebaum. Comparison of neonatal and maternal outcomes in nulliparous women 40 years or older with spontaneous versus IVF autologous egg or IVF donor egg singleton pregnancies. 2011. 96 (3 SUPPL. 1): S182.
31. Marklund A, Nasiell J, Berger AS, Fagerberg A, Rodriguez-Wallberg KA. Pregnancy achieved using donor eggs in cancer survivors with treatment-induced ovarian failure: obstetric and perinatal outcome. Journal of Women's Health. 2018. 27(7):939-45.
32. Woo I, Hindoyan R, Landay M, Ho J, Ingles SA, McGinnis LK, Paulson RJ, Chung K. Perinatal outcomes after natural conception versus in vitro fertilization (IVF) in gestational surrogates: a model to evaluate IVF treatment versus maternal effects. Fertility and sterility. 2017. 108(6):993-8. gestational surrogates
33. Sunkara SK, Antonisamy B, Kamath MS. Perinatal outcomes following gestational surrogacy versus autologous IVF: analysis of 87,815 singleton live births. Human Reproduction. 2017. 32(1): 56.
34. Preaubert L, Vincent A, Santulli P, Gayet V, Goffinet F, Le Ray C. 920: Outcomes of pregnancies achieved by double gamete donation: a comparison with donor oocyte pregnancies. American Journal of Obstetrics & Gynecology. 2017. 216(1): S524-5.
35. S. Saito, Y. Nakabayashi, A. Nakashima, T. Shima and O. Yoshino. A new era in reproductive medicine: consequences of third-party oocyte donation for maternal and fetal health. 2016. 38 (6): 687-697.
36. Thurin-Kjellberg, S. Nejdet, K. Kallen and U. B. Wennerholm. Maternal and perinatal outcomes in oocyte donation pregnancies in Sweden 2003-2012. 2015. 30: i373.
37. M. N. Baumgarten, D. Stoop, P. Haentjes, G. Verheyen, F. De Schrijver, I. Liebaers, M. Camus, M. Bonduelle and P. Devroey. Oocyte donation is a risk factor for first trimester bleeding and pregnancy induced hypertension but without effect on the perinatal outcome. 2010. 25: i237-i238.
38. T. Tarlatzi, R. Imbert, I. Demeestere, C. Venetis, Y. Englert and A. Delbaere. Is oocyte donation an independent risk factor for preeclampsia: A propensity score analysis. 2014. 102 (3 SUPPL. 1): e111.
39. U. B. Wennerholm, S. Nejdet, K. Kallen and A. Thurin. Obstetric outcome after oocyte donation (OD). 2015. 212 (1 SUPPL. 1): S52.
40. M. Kamath, B. Antonisamy and S. K. Sunkara. Perinatal outcomes following oocyte donation versus autologous IVF: Analysis of 99,111 singleton live births. 2016. 31: i6-i7.
41. S. Lin, T. Singer, E. Milbank, N. Ward, D. Skupski and A. Grunebaum. Neonatal and maternal outcomes in nulliparous women 40 years or older with spontaneous, IVF autologous oocyte or IVF donor oocyte singleton pregnancies. 2012. 19 (3 SUPPL. 1): 285A.
42. S. Nejdet,​ C. Bergh,​ K. Kallen,​ U.-B. Wennerholm and A. Thurin-Kjellberg. High risks of maternal and perinatal complications in singletons born after oocyte donation. 2016. 95 (8): 879-86.
43. S. K. Kalra,​ S. J. Ratcliffe,​ C. Coutifaris,​ T. Molinaro and K. T. Barnhart. Ovarian stimulation and low birth weight in newborns conceived through in vitro fertilization. 2011. 118 (4): 863-71.
44. S. Antinori,​ C. Versaci,​ C. Panci,​ B. Caffa and G. H. Gholami. Fetal and maternal morbidity and mortality in menopausal women aged 45-63 years. 1995. 10 (2): 464-9.
45. M. V. Sauer,​ J. G. Wang,​ N. C. Douglas,​ G. S. Nakhuda,​ P. Vardhana,​ V. Jovanovic and M. M. Guarnaccia. Providing fertility care to men seropositive for human immunodeficiency virus: reviewing 10 years of experience and 420 consecutive cycles of in vitro fertilization and intracytoplasmic sperm injection. 2009. 91 (6): 2455-60.
46. J. C. Dumoulin,​ J. A. Land,​ A. P. Van Montfoort,​ E. C. Nelissen,​ E. Coonen,​ J. G. Derhaag,​ I. L. Schreurs,​ G. A. Dunselman,​ A. D. Kester,​ J. P. Geraedts and J. L. Evers. Effect of in vitro culture of human embryos on birthweight of newborns. 2010. 25 (3): 605-12.
47. C. Srisombut,​ A. Rojanasakul,​ C. Suchartwatnachai,​ W. Choktanasiri,​ S. Weerakiet and S. Chinsomboon. Outcome of pregnancy in IVF-ET cycle at Ramathibodi Hospital. 1995. 78 (12): 657-61.
48. C. Wang,​ G. Feng,​ B. Zhang,​ H. Zhou,​ J. Shu,​ R. Lin,​ H. Chen and Z. Wu. Effect of different artificial shrinkage methods,​ when applied before blastocyst vitrification,​ on perinatal outcomes. 2017. 15 (1): 32.
49. D. H. Kort,​ J. Gosselin,​ J. M. Choi,​ M. H. Thornton,​ J. Cleary-Goldman and M. V. Sauer. Pregnancy after age 50: defining risks for mother and child. 2012. 29 (4):245-50.
50. N. Chevalier,​ H. Letur,​ D. Lelannou,​ J. Ohl,​ D. Cornet,​ C. Chalas-Boissonnas,​ R. Frydman,​ S. Catteau-Jonard,​ T. Greck-Chassain,​ A. Papaxanthos-Roche,​ M.-C. Dulucq,​ M.-L. Couet,​ I. Cedrin-Durnerin,​ J.-L. Pouly,​ P. Fenichel and D. French Study Group for Oocyte. Materno-fetal cardiovascular complications in Turner syndrome after oocyte donation: insufficient prepregnancy screening and pregnancy follow-up are associated with poor outcome. 2011. 96 (2): E260-7.
51. H. I. Abdalla and A. K. S. Kan. Outcome of ovum donation pregnancies. 1998. 10 (3): 165-170.
52. Guilbaud L, Santulli P, Studer E, Gayet V, Goffinet F, Le Ray C. Impact of oocyte donation on perinatal outcome in twin pregnancies. Fertility and sterility. 2017. 107(4):948-53.
53. H. I. Abdalla, A. Billett, A. K. Kan, S. Baig, M. Wren, L. Korea and J. W. Studd. Obstetric outcome in 232 ovum donation pregnancies. 1998. 105 (3): 332-7.
54. Rodriguez-Wallberg K.A.; Berger A.-S.; Fagerberg A.; Olofsson J.I.; Scherman-Pukk C.; Lindqvist P.G.; Nasiell J. Increased incidence of obstetric and perinatal complications in pregnancies achieved using donor oocytes and single embryo transfer in young and healthy women. A prospective hospital-based matched cohort study. Gynecological Endocrinology. (Nasiell) Department of Obstetrics and Gynecology, Karolinska University Hospital, Stockholm, Sweden. 2018.
55. Yu B, Vega M, Zaghi S, Fritz R, Jindal S, Buyuk E. Comparison of perinatal outcomes following frozen embryo transfer cycles using autologous versus donor oocytes in women 40 to 43 years old: analysis of SART CORS data. Journal of assisted reproduction and genetics. 2018. 35(11):2025-9.
56. Melo P, Supramaniam PR, Mittal M, Lim LN. Oocyte donation is not an independent risk factor for adverse perinatal outcomes in IVF pregnancies: results of a 5-year cohort in a UK high-risk unit. Human Reproduction. 2018. 33: 496-497.
57. Dior UP, Laufer N, Chill HH, Granovsky-Grisaru S, Yagel S, Yaffe H, Gielchinsky Y. Increased incidence of preeclampsia in mothers of advanced age conceiving by oocyte donation. Archives of gynecology and obstetrics. 2018. 297(5): 1293-9.
58. Boria F.; Cabrero M.; Mateos A.; Martin-Camean M.; De La Calle M. Obstetric and neonatal complications in twin pregnancies after oocyte donation. Twin Research and Human Genetics. 2017. 20(6): 629.
59. Boria F, de la Calle M, Cuerva M, Sainz A, Bartha JL. Impact of oocyte donation on obstetric and perinatal complications in twin pregnancies. The Journal of Maternal-Fetal & Neonatal Medicine. 2018 Nov 16:1-4.
60. Preaubert L, Vincent-Rohfritsch A, Santulli P, Gayet V, Goffinet F, Le Ray C. Outcomes of pregnancies achieved by double gamete donation: A comparison with pregnancies obtained by oocyte donation alone. European Journal of Obstetrics & Gynecology and Reproductive Biology. 2018. 222:1-6.
61. Massiah N, Pearce K, Choudhary M. Trends and reproductive outcomes of double gamete embryo donations: An analysis of a national data registry. Human Reproduction 2017. 32(1): S322.
62. Kamath MS, Antonisamy B, Mascarenhas M, Sunkara SK. High-risk of preterm birth and low birth weight after oocyte donation IVF: analysis of 133,785 live births. Reproductive biomedicine online. 2017 Sep 1;35(3):318-24.
63. Y. Yaron,​ Y. Ochshorn,​ A. Amit,​ A. Kogosowski,​ I. Yovel and J. B. Lessing. Oocyte donation in Israel: a study of 1001 initiated treatment cycles. 1998. 13 (7): 1819-24.
64. W. Shih,​ D. D. Rushford,​ H. Bourne,​ C. Garrett,​ J. C. McBain,​ D. L. Healy and H. W. G. Baker. Factors affecting low birthweight after assisted reproduction technology: Difference between transfer of fresh and cryopreserved embryos suggests an adverse effect of oocyte collection. 2008. 23 (7): 1644-1653.
65. V. Soderstrom-Anttila,​ A. Tiitinen,​ T. Foudila and O. Hovatta. Obstetric and perinatal outcome after oocyte donation: comparison with in-vitro fertilization pregnancies. 1998. 13 (2): 483-90.
66. T. B. Tarlatzi,​ R. Imbert,​ B. Alvaro Mercadal,​ I. Demeestere,​ C. A. Venetis,​ Y. Englert and A. Delbaere. Does oocyte donation compared with autologous oocyte IVF pregnancies have a higher risk of preeclampsia?. 2017. 34 (1): 11-18.
67. S. R. Soares,​ C. Troncoso,​ E. Bosch,​ V. Serra,​ C. Simon,​ J. Remohi and A. Pellicer. Age and uterine receptiveness: predicting the outcome of oocyte donation cycles. 2005. 90 (7): 4399-404.
68. S. Malchau,​ A. Loft,​ S. Rasmusen,​ E. C. Larsen,​ A. Nyboe Andersen and A. Pinborg. Perinatal outcome in 375 children born after Oocyte Donation n a Danish national controlled cohort study. 2012. 27.
69. M. Storgaard,​ A. Loft,​ C. Bergh,​ U. B. Wennerholm,​ V. Soderstrom-Anttila,​ L. B. Romundstad,​ K. Aittomaki,​ N. Oldereid,​ J. Forman and A. Pinborg. Obstetric and neonatal complications in pregnancies conceived after oocyte donation: a systematic review and meta-analysis. 2017. 124 (4): 561-572.
70. L. H. Sekhon,​ R. S. Gerber,​ A. Rebarber,​ D. H. Saltzman,​ C. K. Klauser,​ S. Gupta and N. S. Fox. Effect of oocyte donation on pregnancy outcomes in in vitro fertilization twin gestations. 2014. 101 (5): 1326-30.
71. L. Guilbaud,​ P. Santulli,​ E. Studer,​ V. Gayet,​ F. Goffinet and C. Le Ray. Impact of oocyte donation on perinatal outcome in twin pregnancies. 2017. 107 (4): 948-953.e1.
72. G. Sheffer-Mimouni,​ S. Mashiach,​ J. Dor,​ D. Levran and D. S. Seidman. Factors influencing the obstetric and perinatal outcome after oocyte donation. 2002. 17 (10): 2636-40.
73. G. Pados,​ M. Camus,​ A. Van Steirteghem,​ M. Bonduelle and P. Devroey. The evolution and outcome of pregnancies from oocyte donation. 1994. 9 (3): 538-42.
74. E. Clua,​ E. Meler,​ D. Rodriguez,​ B. Coroleu,​ I. Rodriguez,​ F. Martinez and R. Tur. Obstetric and perinatal complications in an oocyte donation programme. Is it time to limit the number of embryos to transfer?. 2016. 32 (4): 267-71.
75. E. Elenis,​ G. Sydsjo,​ A. Skalkidou,​ C. Lampic and A. S. Svanberg. Neonatal outcomes in pregnancies resulting from oocyte donation: a cohort study in Sweden. 2016. 16 (1): 170.
76. D. Stoop,​ M. Baumgarten,​ P. Haentjens,​ N. P. Polyzos,​ M. De Vos,​ G. Verheyen,​ M. Camus and P. Devroey. Obstetric outcome in donor oocyte pregnancies: A matched-pair analysis. 2012. 10.
77. D. Galliano,​ N. Garrido,​ V. Serra-Serra and A. Pellicer. Difference in birth weight of consecutive sibling singletons is not found in oocyte donation when comparing fresh versus frozen embryo replacements. 2015. 104 (6): 1411-3.
78. D. Ameratunga,​ G. Weston,​ T. Osianlis,​ J. Catt and B. Vollenhoven. In vitro fertilisation (IVF) with donor eggs in post-menopausal women: Are there differences in pregnancy outcomes in women with premature ovarian failure (POF) compared with women with physiological age-related menopause?. 2009. 26 (9-10): 511-514.
79. C. Le Ray,​ S. Scherier,​ O. Anselem,​ A. Marszalek,​ V. Tsatsaris,​ D. Cabrol and F. Goffinet. Association between oocyte donation and maternal and perinatal outcomes in women aged 43 years or older. 2012. 27 (3): 896-901.
80. F. Gundogan,​ D. W. Bianchi,​ S. A. Scherjon and D. J. Roberts. Placental pathology in egg donor pregnancies. 2010. 93 (2): 397-404.
81. S. S. Malchau,​ A. Loft,​ E. C. Larsen,​ A.-K. Aaris Henningsen,​ S. Rasmussen,​ A. N. Andersen and A. Pinborg. Perinatal outcomes in 375 children born after oocyte donation: a Danish national cohort study. 2013. 99 (6): 1637-43.
82. A. Cobo,​ M. Meseguer,​ M. Morgan,​ S. Fortno,​ V. Serra and J. Remohi. Obstetric and perinatal outcome of babies born after oocyte vitrification. 2010. 94 (4 SUPPL. 1): S68.
83. A. Cobo,​ V. Serra,​ N. Garrido,​ I. Olmo,​ A. Pellicer and J. Remohi. Obstetric and perinatal outcome of babies born from vitrified oocytes. 2014. 102 (4): 1006-1015.e4.
84. Hagman,​ A. Loft,​ U.-B. Wennerholm,​ A. Pinborg,​ C. Bergh,​ K. Aittomaki,​ K.-G. Nygren,​ L. Bente Romundstad,​ J. Hazekamp and V. Soderstrom-Anttila. Obstetric and neonatal outcome after oocyte donation in 106 women with Turner syndrome: a Nordic cohort study. 2013. 28 (6): 1598-609.
85. A. L. Tranquilli,​ V. Biondini,​ S. Talebi Chahvar,​ A. Corradetti,​ D. Tranquilli and S. Giannubilo. Perinatal outcomes in oocyte donor pregnancies. 2013. 26 (13): 1263-7.
86. A. M. Dude,​ J. S. Yeh and S. J. Muasher. Donor oocytes are associated with preterm birth when compared to fresh autologous in vitro fertilization cycles in singleton pregnancies. 2016. 106 (3): 660-5.
87. B. A. Mercadal,​ R. Imbert,​ I. Demeestere,​ Y. Englert and A. Delbaere. Pregnancy outcome after oocyte donation in patients with Turners syndrome and partial X monosomy. 2011. 26 (8): 2061-2068.
88. B. Kallen,​ O. Finnstrom,​ A. Lindam,​ E. Nilsson,​ K.-G. Nygren and P. O. Olausson. Blastocyst versus cleavage stage transfer in in vitro fertilization: differences in neonatal outcome?. 2010. 94 (5): 1680-3.
89. C. Wood, A. Trounson, J. F. Leeton, P. M. Renou, W. A. Walters, B. W. Buttery, J. C. Grimwade, J. C. Spensley and V. Y. Yu. Clinical features of eight pregnancies resulting from in vitro fertilization and embryo transfer. 1982. 38 (1): 22-9.

**Siblings/ Vanishing twins/multiples/not singleton**

1. A. Strauss, B. W. Paek, O. Genzel-Boroviczeny, A. Schulze, U. Janssen and H. Hepp. Multifetal gestation--maternal and perinatal outcome of 112 pregnancies. 2002. 17 (4): 209-17.
2. C. Centers for Disease and Prevention. Contribution of assisted reproductive technology and ovulation-inducing drugs to triplet and higher-order multiple births--United States, 1980-1997. 2000. 49 (24): 535-8.
3. Anonymous. Multiple gestation pregnancy. The ESHRE Capri Workshop Group. 2000. 15 (8): 1856-64.
4. M. L. Ho, J. Y. Chen, U. P. Ling, J. H. Chen, C. M. Huang, C. C. Chang and P. H. Su. Changing epidemiology of triplet pregnancy: etiology and outcome over twelve years. 1996. 13 (5): 269-75.
5. N. Mordel, G. Zajicek, A. Benshushan, J. G. Schenker, N. Laufer and E. Sadovsky. Elective suture of uterine cervix in triplets. 1993. 10 (1): 14-6.
6. S. Lipitz, B. Reichman, G. Paret, M. Modan, J. Shalev, D. M. Serr, S. Mashiach and Y. Frenkel. The improving outcome of triplet pregnancies. 1989. 161 (5): 1279-84.
7. A. Tandberg, T. Bjorge, O. Nygard, P. E. Bordahl and R. Skjaerven. Trends in incidence and mortality for triplets in Norway 1967-2006: The influence of assisted reproductive technologies. 2010. 117 (6): 667-675.
8. L. Keith and G. Breborowicz. Triplet pregnancies and their aftermaths. Part I: Basic considerations. 2002. 47 (6): 254-64.
9. A. K. Oleszczuk, J. J. Oleszczuk and L. G. Keith. Defining the high-risk nature of triplet pregnancies. 2002. 47 (4): 182-90.
10. G. Zanconato, S. Poggi, R. Ruffo, A. Gazzoni, E. M. Padovani and M. Franchi. Antepartum management and neonatal outcome of triplet pregnancies. 2005. 271 (4): 320-4.
11. S. A. Al-Suleiman, F. E. Al-Jama, J. Rahman and M. S. Rahman. Obstetric complications and perinatal outcome in triplet pregnancies. 2006. 26 (3): 200-4.
12. J. L. Eaton, X. Zhang and R. R. Kazer. First-trimester bleeding and twin pregnancy outcomes following in vitro fertilization (IVF). 2014. 21 (3 SUPPL. 1): 235A.
13. G. Chistyakova, I. Gazieva, I. Remizova, L. Ustyantseva, V. Lyapunov and S. Bychkova. Risk factors vary early preterm birth and perinatal complications after assisted reproductive technology. 2016. 32 (sup2): 56-61.
14. B. Luke, D. Gopal, H. Cabral, H. Diop and J. E. Stern. Perinatal outcomes of singleton siblings: the effects of changing maternal fertility status. 2016. 33 (9): 1203-13.
15. N. S. Fox, E. M. Stern, D. H. Saltzman, C. K. Klauser, S. Gupta and A. Rebarber. The association between maternal weight gain and spontaneous preterm birth in twin pregnancies. 2014. 27 (16): 1652-5.
16. A. Ghalili, A. McLennan, L. Pedersen, G. Kesby and J. Hyett. Outcomes of monochorionic diamniotic twin pregnancies: a comparison of assisted and spontaneous conceptions. 2013. 53 (5): 437-42.
17. S. Kawachiya, D. Bodri, N. Shimada, K. Kato, Y. Takehara and O. Kato. Blastocyst culture is associated with an elevated incidence of monozygotic twinning after single embryo transfer. 2011. 95 (6): 2140-2.
18. K. Morcel, V. Lavoue, A. Beuchee, D. Le Lannou, P. Poulain and P. Pladys. Perinatal morbidity and mortality in twin pregnancies with dichorionic placentas following assisted reproductive techniques or ovarian induction alone: a comparative study. 2010. 153 (2): 138-42.
19. C. O. Brock and C. Gyamfi-Bannerman. Assisted reproductive technology and preterm delivery in twin gestations. 2017. 24 (1 Supplement 1): 248A.
20. E. Mierzejewska and K. Szamotulska. Trends in occurrence of dizygotic and monozygotic twins in Poland: An indicator of the frequency of assisted reproductive technology treatments?. 2012. 27 (1 SUPPL. 1): S49-S50.
21. J. Holte, L. Berglund, N. Hadziosmanovic, J. Tilly, H. Pettersson and T. Bergh. The construction and validation of a prediction model to minimize twin rates at preserved live birth rates in ART. 2011. 26: i62.
22. L. Sperling, C. Kiil, L. U. Larsen, I. Qvist, M. Schwartz, C. Jorgensen, K. Skajaa, J. Bang and A. Tabor. Naturally conceived twins with monochorionic placentation have the highest risk of fetal loss. 2006. 28 (5): 644-52.
23. R. Kalish. Comparison of neonatal outcomes in singleton, twin and triplet pregnancies conceived with in vitro fertilization. 2014. 210 (1 SUPPL. 1): S117.
24. B. Blondel, P. Tuppin, M. Kaminski and G. Breart. Multiple pregnancies. Evolution of the frequency and perinatal risk. 1994. 7 (1-2): 9-14.
25. B. Luke, D. Gopal, H. Cabral, J. E. Stern and H. Diop. Adverse pregnancy, birth, and infant outcomes in twins: effects of maternal fertility status and infant gender combinations; the Massachusetts Outcomes Study of Assisted Reproductive Technology. 2017.
26. M. E. Geisler, A. O'Mahony, S. Meaney, J. J. Waterstone and K. O'Donoghue. Obstetric and perinatal outcomes of twin pregnancies conceived following IVF/ICSI treatment compared with spontaneously conceived twin pregnancies. 2014. 181: 78-83.
27. Gyamfi-Bannerman and R. S. Miller. The role of 17-OHPC in preventing recurrent preterm birth in a current twin gestation. 2015. 22: 151A.
28. J. Seitz, J. Williams, C. Bamrick and J. Ayers. Decreased human chorionic gonadotropin and preterm delivery in dichorionic twin gestations. 2014. 123: 78S.
29. Gerber R, Fields J, Barberio A, Bodenlos K, Fox N. Outcomes of Twin Pregnancies in Women Age 45 or Older [30R]. Obstetrics & Gynecology. 2017. 129:190S-1S.
30. S. Amrane, P. Ghosh, D. E. Reichman, Z. Rosenwaks and S. E. Gelber. Maternal and neonatal outcomes in women of advanced maternal age (AMA) undergoing two in vitro fertilization (IVF) singleton pregnancies, as compared to one IVF twin pregnancy. 2016. 106: e34.
31. Trivedi K. Twin pregnancy: Maternal and fetal outcome. International Journal of Gynecology and Obstetrics. 2018. 143 Supplement 3: 623.
32. Yee L, Caughey A, Grobman W, Cheng Y. Relationship between mode of conception and perinatal outcomes among US women with twin gestations. American Journal of Obstetrics and Gynecology. 2017. 216(1): S295-S295.
33. Okby R, Al Atawna A, Wainstock T, Sergienko R, Sheiner E. 978: Placental abruption in twin pregnancies, risk factors and perinatal outcomes. American Journal of Obstetrics & Gynecology. 2017. 216(1): S551.
34. C. A. M. Jansen, H. C. Van Os, P. M. Rijnders and K. E. Tucker. The obstetric outcome of twin pregnancies after Double Embryo Transfer (DET) in patients "deselected" for risk factors of premature delivery. 2009. 24: i126.
35. Sazonova, K. Kuallen, A. Thurin-Kjellberg, U. B. Wennerholm and C. Bergh. Dramatically impaired outcome for one IVF twin pregnancy compared to two IVF singleton pregnancies. 2012. 98 (3 SUPPL. 1): S273.
36. S. N. Lin, T. Singer, D. Lee, S. Purisch, D. Skupski and A. Grunebaum. Neonatal and maternal outcomes in women 40 years and older with IVF autologous oocyte and IVF donor oocyte twin pregnancies. 2012. 19 (3 SUPPL. 1): 285A-286A.
37. S. Sapra, K. J. Hensel, I. V. Landres, S. Jaffe and S. E. Gelber. Associations between maternal age and obstetric and perinatal outcomes in twin IVF pregnancies. 2012. 19 (3 SUPPL. 1): 113A.
38. Pinborg. Vanishing twins in in-vitro fertilization (IVF). 2010. 13 (3): 283.
39. Barda G, Gluck O, Mizrachi Y, Bar J. A comparison of maternal and perinatal outcome between in vitro fertilization and spontaneous dichorionic-diamniotic twin pregnancies. The Journal of Maternal-Fetal & Neonatal Medicine. 2017. 30(24):2974-7.
40. Tiitinen. Elective singe embry transfer and multiple births - Trends in Nordic countries. 2012. 91: 40.
41. Bordi G, D’Ambrosio A, Gallotta I, Di Benedetto L, Frega A, Torcia F, Schimberni M, Bonito M, Caserta D. The influence of ovulation induction and assisted conception on maternal and perinatal outcomes of twin pregnancies. European review for medical and pharmacological sciences. 2017. 21:3998-4006.
42. Wang Y.A.; Safi N.; Ali F.; Lui K.; Li Z.; Umstad M.; Sullivan E. Increased rate of inferior neonatal outcomes among twins following assisted reproductive technology. Human Reproduction. 2017. 32(1).
43. Brock CO, Chauhan SP, Blackwell SC, Sibai BM. 223: Pregnancy outcome in twins gestations: Spontaneous vs. assisted reproductive modes of conception. American Journal of Obstetrics & Gynecology. 2018. 218(1):S146-7.
44. Daskalakis G, Antsaklis P, Gourounti K, Theodora M, Sindos M, Papantoniou N, Loutradis D. Chorionic Villus Sampling in Assisted Versus Spontaneous Conception Twins. Ultraschall in der Medizin-European Journal of Ultrasound. 2017. 38(04):437-42.
45. Tabanelli, A. P. Ferraretti, E. Feliciani, C. Cetera, M. Gergolet, M. C. Magli and L. Gianaroli. Vanishing twins in IVF/ICSI pregnancies: A case-control study. 2009. 24: i130-i131.
46. Vieira L, Warren L, Robles B, Pan S, Overbey J, Ferrara L, Stone J. 530: Comparison of pregnancy outcomes in 2-to-1 multifetal pregnancy reduction and ongoing twins. American Journal of Obstetrics & Gynecology. 2018. 218(1):S317-8.
47. Shah J, Nasab SH, Chen H, Chappell NR, Schutt A, Mendez-Figueroa H. Pregnancy outcomes among twins stratified by method of conception, a secondary analysis of maternal fetal medicine units (MFMU) network database. Fertility and Sterility. 2017. 108(3):e350.
48. L. Meints and D. Akers. Effect of conception type on pregnancy outcomes in twin gestations. 2016. 23 (1 SUPPL. 1): 271A.
49. Pinborg, G. O. Lykkegaard, A. Loft, S. C. Rasmussen and H. J. Ingerslev. Cervical conisation and risk of preterm delivery in assisted reproductive technology (ART) singleton and twin pregnancies-Danish national cohort study. 2014. 29: i67-i68.
50. Takyi A, Gyamfi-Bannerman C. The Association between Assisted Reproductive Technology (ART) and Preterm Delivery (PTD) in Twin Pregnancies. InREPRODUCTIVE SCIENCES 2018. 25: 180A-181A.
51. L. Sekhon, K. Shaia, J. Rodriguez-Purata, J. A. Lee and A. B. Copperman. Does fresh embryo transfer accentuate the perinatal risks of dichorionic twin gestations?. 2016. 106: e202.
52. Běhávková K, Krofta L, Macková K, Vojtěch J, Hašlík L, Pock R, Hympánová L, Kučerová M, Heřman H, Straňák Z, Feyereisl J. Retrospective analysis of monochorionic twin pregnancies born in the Institute for the Care of Mother and Child between 2012-2015. Ceska gynekologie. 2017. 82(3):180-9.
53. Z. Breen, T. Child, A. Gwynn, E. McVeigh and K. Turner. Comparison of pregnancy outcomes for singletons and twins created following IVF and ICSI treatment over a two year period at the oxford fertility unit. 2014. 28: S4.
54. Luke B, Gopal D, Cabral H, Diop H, Stern J. Adverse pregnancy, birth, and infant outcomes in twins: effects of method of conception and zygosity. American Journal of Obstetrics and Gynecology. 2017. 216(1): S497.
55. Luo L, Jie H, Chen M, Wang Q. Is it worth reducing twins to singletons after IVF-ET? a retrospective cohort study using propensity score matching. Fertility and Sterility. 2017. 108(3):e378-9.
56. O. Health Quality. In vitro fertilization and multiple pregnancies: an evidence-based analysis. 2006. 6 (18): 1-63.
57. Ozek M.A.; Karaagaoglu E.; Orgul G.; Gumruk F.; Yurdakok M.; Sinan Beksac M. Vanished twin: Obstetric and perinatal outcomes and association with methylenetetrahydrofolate reductase (mthfr) polymorphisms(s). Twin Research and Human Genetics. 2017. 20(6): 605.
58. Lopez Y.C.; Ortuno P.A.; Larroca S.G.-T.; Hernandez I.C.; De Leon Luis J.A. Perinatal outcome according to chorionicity in twin pregnancy. Twin Research and Human Genetics. 2017. 20(6): 611.
59. Pinborg, G. Ortoft, A. Loft and H. J. Ingerslev. Cervical conisation doubles the risk of preterm and very preterm delivery in ART twins-national controlled cohort stury. 2014. 102 (3 SUPPL. 1): e60.
60. Romanski P, Brady P, Kaser D, Assens M, Walsh B, Carusi D, Missmer S, Farland L, Mallard C, Racowsky C. Is a viable fetus in a vanishing twin before demise associated with obstetrical outcomes of the surviving twin?. Fertility and Sterility. 2017. 107(3): e12.
61. Romanski P, Brady P, Farland L, Carusi D, Missmer S, Kaser D, Walsh B, Mallard C, Racowsky C. Perinatal and maternal outcomes in vanishing twin pregnancies achieved by in vitro fertilization. Fertility and Sterility. 2018. 109(3): e45.
62. J. P. Elliott and T. G. Radin. Quadruplet pregnancy: contemporary management and outcome. 1992. 80 (3 Pt 1): 421-4.
63. Margaux A, Phillips S, Ferrieres-Hoa A, Gala A, Fournier A, Vincens C, Vintejoux E, Bissonnette F, Kadoch IJ, Hamamah S. Embryo cryopreservation is associated with significantly higher birth weight compared with sibling cohort fresh embryo transfer: a bicenter cohort. Human Reproduction. 2018. 33: 224-224.
64. A.-K. A. Henningsen,​ A. Pinborg,​ O. Lidegaard,​ C. Vestergaard,​ J. L. Forman and A. N. Andersen. Perinatal outcome of singleton siblings born after assisted reproductive technology and spontaneous conception: Danish national sibling-cohort study. 2011. 95 (3): 959-63.
65. D. W. Skupski. Maternal complications of twin gestation. 1996. 21 (4): 72-84.
66. A. Pinborg,​ A. A. Henningsen,​ A. Loft,​ S. S. Malchau,​ J. Forman and A. N. Andersen. Large baby syndrome in singletons born after frozen embryo transfer (FET): is it due to maternal factors or the cryotechnique?. 2014. 29 (3): 618-27.
67. Luke B, Brown MB, Wantman E, Stern JE, Toner JP, Coddington CC. Increased risk of large-for-gestational age birthweight in singleton siblings conceived with in vitro fertilization in frozen versus fresh cycles. Journal of assisted reproduction and genetics. 2017. 34(2):191-200.
68. Opdahl S, Henningsen AA, Tiitinen A, Bergh C, Pinborg A, Romundstad PR, Wennerholm UB, Gissler M, Skjærven R, Romundstad LB. Risk of hypertensive disorders in pregnancies following assisted reproductive technology: a cohort study from the CoNARTaS group. Human Reproduction. 2015. 30(7):1724-31.
69. A. Pinborg, A. Loft, L. Noergaard, A. A. Henningsen, S. Rasmussen and A. Nyboe Andersen. Singletons born after frozen embryo transfer (FET) have an increased risk of being large for gestational age n Danish National controlled cohort study of 15078 singletons. 2011. 26: i92-.
70. B. Luke,​ M. B. Brown,​ E. Wantman,​ J. E. Stern,​ J. P. Toner and C. C. Coddington,​ 3rd. Increased risk of large-for-gestational age birthweight in singleton siblings conceived with in vitro fertilization in frozen versus fresh cycles. 2017. 34 (2): 191-200.
71. N. Pereira,​ K. P. Pryor,​ A. C. Petrini,​ J. P. Lekovich,​ J. Stahl,​ R. T. Elias and S. D. Spandorfer. Perinatal Risks Associated with Early Vanishing Twin Syndrome following Transfer of Cleavage- or Blastocyst-Stage Embryos. 2016.
72. P. De Sutter,​ J. Bontinck,​ V. Schutysers,​ J. Van der Elst,​ J. Gerris and M. Dhont. First-trimester bleeding and pregnancy outcome in singletons after assisted reproduction. 2006. 21 (7): 1907-11.
73. A. Pinborg, O. Lidegaard, N. la Cour Freiesleben and A. N. Andersen. Consequences of vanishing twins in IVF/ICSI pregnancies. 2005. 20 (10): 2821-9.
74. Romanski PA, Carusi DA, Farland LV, Missmer SA, Kaser DJ, Walsh BW, Racowsky C, Brady PC. Perinatal and peripartum outcomes in vanishing twin pregnancies achieved by in vitro fertilization. Obstetrics & Gynecology. 2018. 131(6):1011-20.
75. Kamath MS, Antonisamy B, Selliah HY, Sunkara SK. Perinatal outcomes of singleton live births with and without vanishing twin following transfer of multiple embryos: analysis of 113 784 singleton live births. Human Reproduction. 2018. 33(11):2018-22.
76. Magnus MC, Ghaderi S, Morken NH, Magnus P, Bente Romundstad L, Skjærven R, Wilcox AJ, Eldevik Håberg S. Vanishing twin syndrome among ART singletons and pregnancy outcomes. Human Reproduction. 2017. 32(11):2298-304.
77. O. Shebl,​ T. Ebner,​ M. Sommergruber,​ A. Sir and G. Tews. Birth weight is lower for survivors of the vanishing twin syndrome: a case-control study. 2008. 90 (2): 310-4.
78. S. E. Barton,​ S. A. Missmer and M. D. Hornstein. Twin pregnancies with a 'vanished' embryo: a higher risk multiple gestation group?. 2011. 26 (10): 2750-3.
79. R. Mansour,​ O. Ishihara,​ G. D. Adamson,​ S. Dyer,​ J. de Mouzon,​ K. G. Nygren,​ E. Sullivan and F. Zegers-Hochschild. International Committee for Monitoring Assisted Reproductive Technologies world report: Assisted Reproductive Technology 2006. 2014. 29 (7): 1536-51.
80. N. Zhang,​ H. Chen,​ Z. Xu,​ B. Wang,​ H. Sun and Y. Hu. Pregnancy,​ Delivery,​ and Neonatal Outcomes of In Vitro Fertilization-Embryo Transfer in Patient with Previous Cesarean Scar. 2016. 22: 3288-95.
81. A. Wisanto,​ M. Bonduelle,​ M. Camus,​ H. Tournaye,​ M. Magnus,​ I. Liebaers,​ A. Van Steirteghem and P. Devroey. Obstetric outcome of 904 pregnancies after intracytoplasmic sperm injection. 1996. 11 Suppl 4: 121-130.
82. M. Dommergues,​ I. Nisand,​ L. Mandelbrot,​ E. Isfer,​ N. Radunovic and Y. Dumez. Embryo reduction in multifetal pregnancies after infertility therapy: obstetrical risks and perinatal benefits are related to operative strategy. 1991. 55 (4): 805-11.
83. K. Coyne,​ L. D. Whigham,​ K. O'Leary,​ J. K. Yaklic,​ R. A. Maxwell and S. R. Lindheim. Gestational carrier BMI and reproductive,​ fetal and neonatal outcomes: Are the risks the same with increasing obesity?. 2016. 40 (1): 171-175.
84. J. Parkinson,​ C. Tran,​ T. Tan,​ J. Nelson,​ J. Batzofin and P. Serafini. Perinatal outcome after in-vitro fertilization-surrogacy. 1999. 14 (3): .671-6.
85. D. M. Saunders and P. Lancaster. The wider perinatal significance of the Australian in vitro fertilization data collection program. 1989. 6 (2): .252-7.
86. B. Li,​ Y. Ma,​ J. Huang,​ X. Xiao,​ L. Li,​ C. Liu,​ Y. Shi,​ D. Wang and X. Wang. Probing the effect of human normal sperm morphology rate on cycle outcomes and assisted reproductive methods selection. 2014. 9 (11):.e113392.
87. Razaz N, Avitan T, Ting J, Pressey T, Joseph KS. Perinatal outcomes in multifetal pregnancy following fetal reduction. CMAJ. 2017. 189(18):E652- E658.
88. Wang AY, Safi N, Ali F, Lui K, Li Z, Umstad MP, Sullivan EA. Neonatal outcomes among twins following assisted reproductive technology: an Australian population-based retrospective cohort study. BMC pregnancy and childbirth. 2018. 18(1):320.
89. J. L. Eaton,​ X. Zhang and R. R. Kazer. First-trimester bleeding and twin pregnancy outcomes after in vitro fertilization. 2016. 106 (1): 140-143.
90. I. Laskov,​ N. Michaan,​ A. Cohen,​ Z. Tsafrir,​ S. Maslovitz,​ M. Kupferminc,​ J. Lessing and A. Many. Outcome of twin pregnancy in women >=45 years old: a retrospective cohort study. 2013. 26 (7): 669-72.
91. F. Causio,​ T. Leonetti and M. Falagario. Incidence and outcome of multiple pregnancy after in vitro fertilization. 1995. 26 (1): 41-4.
92. B. Luke,​ J. E. Stern,​ M. Kotelchuck,​ E. R. Declercq,​ M. D. Hornstein,​ D. Gopal,​ L. Hoang and H. Diop. Adverse pregnancy outcomes after in vitro fertilization: effect of number of embryos transferred and plurality at conception. 2015. 104 (1): 79-86.
93. B. Luke,​ M. B. Brown,​ J. E. Stern,​ D. A. Grainger,​ N. Klein and M. Cedars. Effect of embryo transfer number on singleton and twin implantation pregnancy outcomes after assisted reproductive technology. 2010. 55 (9-10): 387-94.
94. A. R. Shewale and B. Shewale. Preterm delivery and growth restriction in multifetal pregnancies reduced to twins: Case-control series. 2014. 5 (2) 40-43.
95. A. P. Domingues,​ S. R. Dinis,​ A. Belo,​ D. Couto,​ E. Fonseca and P. Moura. Impact of induced pregnancies in the obstetrical outcome of twin pregnancies. 2014. 101 (1):172-7.
96. F. Leroy, F. Puissant, P. Barlow and G. de Maertelaer. Guidelines for the prevention of multiple pregnancy in treatment by in vitro fertilization. 1990. 39 (3): 371-8.
97. M. Vulic, D. Roje, Z. Mestrovic, T. Strinic, I. Stipic and I. Vrkic. Is there difference in perinatal outcome of singleton and twin pregnancies after assisted conception: two-year experience. 2013. 52 (2): 241-6.
98. A. Ayres and T. R. B. Johnson. Management of multiple pregnancy: prenatal care-part I. 2005. 60 (8): 527-37.
99. S. D'Arpe, S. Franceschetti, M. G. De Stefano, R. D'Amelio, A. M. Maragno, M. Candelieri, L. Muzii and P. Benedetti Panici. The impact of chorionicity and type of conception on maternal-neonatal outcome in twin pregnancies. 2016. 43 (1): 88-92.
100. Okby R, Harlev A, Sacks KN, Sergienko R, Sheiner E. Preeclampsia acts differently in in vitro fertilization versus spontaneous twins. Archives of gynecology and obstetrics. 2018. 297(3):653-8.
101. Gerber RS, Fields JC, Barberio AL, Bodenlos K, Fox NS. Outcomes of twin pregnancies in women 45 years of age or older. Obstetrics & Gynecology. 2017. 129(5):827-30.
102. Feng C, Li WJ, He RH, Sun XW, Wang G, Wang LQ. Impacts of different methods of conception on the perinatal outcome of intrahepatic cholestasis of pregnancy in twin pregnancies. Scientific reports. 2018. 8(1):3985.
103. Oberg AS, VanderWeele TJ, Almqvist C, Hernandez-Diaz S. Pregnancy complications following fertility treatment—disentangling the role of multiple gestation. International journal of epidemiology. 2018. 47(4):1333-42.
104. Hack KE, Vereycken ME, Torrance HL, Koopman‐Esseboom C, Derks JB. Perinatal outcome of monochorionic and dichorionic twins after spontaneous and assisted conception: a retrospective cohort study. Acta obstetricia et gynecologica Scandinavica. 2018. 97(6):717-26.
105. Saccone G, Zullo F, Roman A, Ward A, Maruotti G, Martinelli P, Berghella V. Risk of spontaneous preterm birth in IVF-conceived twin pregnancies. The Journal of Maternal-Fetal & Neonatal Medicine. 2019. 32(3):369-76.
106. Jonsdottir F, Nilas L, Andreasen KR, Grinsted J, Christiansen M, Hedley PL, Naver KV. Obstetrical complications in dichorionic twin pregnancies in women with polycystic ovary syndrome. Acta obstetricia et gynecologica Scandinavica. 2017. 96(12):1453-9.
107. Deltombe‐Bodart S, Deruelle P, Drumez E, Cordiez S, Catteau‐Jonard S, Garabedian C. Obstetrical and perinatal complications of twin pregnancies: is there a link with the type of infertility treatment?. Acta obstetricia et gynecologica Scandinavica. 2017. 96(7):844-51.
108. Meyer R, Orvieto R, Israel A, Mohr-Sasson A, Timerman Y, Gorodesky T, Toussia-Cohen S, Hendler I, Simchen MJ, Machtinger R. Outcomes of singleton versus twin pregnancies in the fifth and sixth decades. European Journal of Obstetrics & Gynecology and Reproductive Biology. 2018. 231:255-61.
109. Luo L, Cai B, Jie HY, Gao Y, Chen M, Zhou CQ, Wang Q. Influence of spontaneous fetal reduction on dichorionic diamniotic twin pregnancy outcomes after in vitro fertilization: a large-sample retrospective study. The journal of maternal-fetal & neonatal medicine: the official journal of the European Association of Perinatal Medicine, the Federation of Asia and Oceania Perinatal Societies, the International Society of Perinatal Obstetricians. 2018.1-6.
110. W. Ombelet,​ I. Cadron,​ J. Gerris,​ P. De Sutter,​ E. Bosmans,​ G. Martens,​ G. Ruyssinck,​ P. Defoort,​ G. Molenberghs and W. Gyselaers. Obstetric and perinatal outcome of 1655 ICSI and 3974 IVF singleton and 1102 ICSI and 2901 IVF twin births: a comparative analysis. 2005. 11 (1): 76-85.
111. T. Simoes,​ A. Queiros,​ A. T. Marujo,​ S. Valdoleiros,​ P. Silva and I. Blickstein. Outcome of monochorionic twins conceived by assisted reproduction. 2015. 104 (3): 629-32.
112. S. Pinzauti,​ C. Ferrata,​ S. Vannuccini,​ G. Di Rienzo,​ F. M. Severi,​ F. Petraglia and M. Di Tommaso. Twin pregnancies after assisted reproductive technologies: the role of maternal age on pregnancy outcome. 2016. 206: 198-203.
113. S. Bhandari,​ I. Ganguly,​ P. Agrawal,​ S. Bhandari,​ A. Singh and N. Gupta. Comparative analysis of perinatal outcome of spontaneous pregnancy reduction and multifetal pregnancy reduction in triplet pregnancies conceived after assisted reproductive technique. 2016. 9 (3): 173-178.
114. R. Bajoria,​ S. B. Ward and A. L. Adegbite. Comparative study of perinatal outcome of dichorionic and trichorionic iatrogenic triplets. 2006. 194 (2): 415-424.
115. R. Chibber,​ M. Fouda,​ W. Shishtawy,​ M. Al-Dossary,​ J. Al-Hijji,​ A. Amen and A. T. Mohammed. Maternal and neonatal outcome in triplet,​ quadruplet and quintuplet gestations following ART: a 11-year study. 2013. 288 (4): 759-67.
116. O. Erez,​ A. Mayer,​ I. Shoham-Vardi,​ D. Dukler and M. Mazor. Primiparity,​ assisted reproduction,​ and preterm birth in twin pregnancies: a population based study. 2008. 277 (4): 311-7.
117. N. Mordel,​ N. Laufer,​ G. Zajicek,​ D. Dorembus,​ A. Benshushan and J. G. E. Schenker Sadovsky. Menotropins as a possible risk factor for premature deliveries in triplet pregnancies. 1991. 5 (3): 197-201.
118. N. Mordel,​ N. Laufer,​ G. Zajicek,​ D. Dorembus,​ A. Benshushan and J. G. E. Schenker Sadovsky. Menotropins as a possible risk factor for premature deliveries in triplet pregnancies. 1991. 5 (3): 197-201.
119. M. R. Dare,​ C. A. Crowther,​ J. M. Dodd and R. J. Norman. Single or multiple embryo transfer following in vitro fertilisation for improved neonatal outcome: a systematic review of the literature. 2004. 44 (4): 283-91.
120. M. I. Evans,​ M. Dommergues,​ R. J. Wapner,​ L. Lynch,​ Y. Dumez,​ J. D. Goldberg,​ I. E. Zador,​ K. H. Nicolaides,​ M. P. Johnson and M. S. Golbus. Efficacy of transabdominal multifetal pregnancy reduction: collaborative experience among the world's largest centers. 1993. 82 (1): 61-6.
121. L.-y. Cai,​ S.-i. Izumi,​ S. Koido,​ N. Uchida,​ T. Suzuki,​ H. Matsubayashi,​ T. Sugi,​ N. Shida,​ K. Kikuchi and K. Yoshikata. Abnormal placental cord insertion may induce intrauterine growth restriction in IVF-twin pregnancies. 2006. 21 (5): 1285-90.
122. L. M. DeLuca,​ N. S. Fox,​ R. S. Green,​ A. Stroustrup,​ M. Harris,​ I. R. Holzman and K. Gibbs. Ovulation induction and small for gestational age neonates in twin pregnancies. 2013. 6 (3): 217-24.
123. K. Ozgur,​ H. Bulut,​ M. Berkkanoglu and K. Coetzee. Perinatal outcomes in singleton and twin ICSI pregnancies following hysteroscopic correction of partial intrauterine septa. 2015. 32 (4): 533-41.
124. J. Haas,​ A. Hourvitz,​ J. Dor,​ S. Elizur,​ Y. Yinon,​ E. Barzilay and A. Shulman. Perinatal outcome of twin pregnancies after early transvaginal multifetal pregnancy reduction. 2014. 101 (5): 1344-1348.
125. J. Haas,​ A. Hourvitz,​ J. Dor,​ Y. Yinon,​ S. Elizur,​ S. Mazaki-Tovi,​ E. Barzilay and A. Shulman. Pregnancy outcome of early multifetal pregnancy reduction: Triplets to twins versus triplets to singletons. 2014. 29 (6): 717-721.
126. I. Ganguly,​ P. Agrawal,​ S. Bhandari,​ A. Singh and N. Gupta. Comparative analysis of perinatal outcome of spontaneous pregnancy reduction and multifetal pregnancy reduction in triplet pregnancies conceived after assisted reproductive technique. 2016. 9 (3): 173-178.
127. G. Fait,​ J. Har-Toov,​ I. Gull,​ J. B. Lessing,​ A. Jaffa and I. Wolman. Cervical length,​ multifetal pregnancy reduction,​ and prediction of preterm birth. 2005. 33 (7): 329-32.
128. F. Olivennes,​ P. Kadhel,​ P. Rufat,​ R. Fanchin,​ H. Fernandez and R. Frydman. Perinatal outcome of twin pregnancies obtained after in vitro fertilization: comparison with twin pregnancies obtained spontaneously or after ovarian stimulation. 1996. 66 (1): 105-9.
129. D. Soriano,​ B. Weisz,​ D. S. Seidman,​ A. Chetrit,​ E. Schiff,​ S. Lipitz and R. Achiron. The role of sonographic assessment of cervical length in the prediction of preterm birth in primigravidae with twin gestation conceived after infertility treatment. 2002. 81 (1): 39-43.
130. D. Shan,​ Y. Hu,​ P. Qiu,​ B. S. Mathew,​ Y. Chen,​ S. Li,​ Y. Hu,​ L. Lin,​ Z. Wang and L. Li. Intrahepatic Cholestasis of Pregnancy in Women With Twin Pregnancy. 2016. 19 (6): 697-707.
131. C.-U. Cheang,​ L.-S. Huang,​ T.-H. Lee,​ C.-H. Liu,​ Y.-T. Shih and M.-S. Lee. A comparison of the outcomes between twin and reduced twin pregnancies produced through assisted reproduction. 2007. 88 (1): 47-52.
132. C. Donner,​ J. A. McGinnis,​ P. Simon and F. Rodesch. Multifetal pregnancy reduction: a Belgian experience. 1991. 38 (3): 183-7.
133. A. Weghofer,​ K. Klein,​ M. Stammler-Safar,​ C. Worda,​ D. H. Barad,​ P. Husslein and N. Gleicher. The impact of fetal gender on prematurity in dichorionic twin gestations after in vitro fertilization. 2010. 8: 57.
134. B. Luke,​ M. B. Brown,​ C. Nugent,​ V. H. Gonzalez-Quintero,​ F. R. Witter and R. B. Newman. Risk factors for adverse outcomes in spontaneous versus assisted conception twin pregnancies. 2004. 81 (2) 315-319.
135. B. Luke,​ M. B. Brown,​ D. A. Grainger,​ J. E. Stern,​ N. Klein and M. I. Cedars. The effect of early fetal losses on twin assisted-conception pregnancy outcomes. 2009. 91 (6): 2586-92.
136. A. Pinborg,​ A. Loft and A. Nyboe Andersen. Neonatal outcome in a Danish national cohort of 8602 children born after in vitro fertilization or intracytoplasmic sperm injection: the role of twin pregnancy. 2004. 83 (11):1071-8.
137. A. Pinborg,​ G. Ortoft,​ A. Loft,​ S. C. Rasmussen and H. J. Ingerslev. Cervical conization doubles the risk of preterm and very preterm birth in assisted reproductive technology twin pregnancies. 2015. 30 (1):197-204.
138. A. Sazonova,​ K. Kallen,​ A. Thurin-Kjellberg,​ U.-B. Wennerholm and C. Bergh. Neonatal and maternal outcomes comparing women undergoing two in vitro fertilization (IVF) singleton pregnancies and women undergoing one IVF twin pregnancy. 2013. 99 (3): 731-7.
139. J. M. Antoine,​ A. Gomes,​ S. Uzan,​ S. Alvarez,​ D. Cornet,​ C. Tibi,​ J. Mandelbaum,​ M. Plachot and J. Salat-Barroux. [Outcome beyond the 1st trimester of 305 pregnancies conceived by fertilization in vitro]. 1990. 19 (7): 901-7.
140. J. Yeh,​ S. Leipzig,​ E. A. Friedman and M. M. Seibel. Results of in vitro fertilization pregnancies: experience at Boston's Beth Israel Hospital. 1990. 35 (2): 116-9.
141. D. M. Saunders and P. Lancaster. The wider perinatal significance of the Australian in vitro fertilization data collection program. 1989. 6 (2): .252-7.
142. P. A. L. Lancaster,​ W. I. H. Johnston,​ W. R. Jones,​ E. C. Wood,​ J. L. Yovich,​ D. M. Saunders,​ C. D. Matthews,​ T. D. Thomas,​ J. F. Hennessey,​ F. M. Graham,​ J. F. Correy,​ D. C. Macourt,​ M. Brinsmead and G. L. Driscoll. In-vitro fertilization pregnancies in Australia and New Zealand,​ 1979-1985. 1988. 148 (9): 429-436.
143. E. R. Myers,​ D. C. McCrory,​ A. A. Mills,​ T. M. Price,​ G. K. Swamy,​ J. Tantibhedhyangkul,​ J. M. Wu and D. B. Matchar. Effectiveness of assisted reproductive technology (ART). 2008. (167): 1-195.
144. H.-J. Wei,​ R. Young,​ I. L. Kuo,​ C.-M. Liaw,​ H.-S. Chiang and C.-Y. Yeh. Abnormal preconception oral glucose tolerance test predicts an unfavorable pregnancy outcome after an in vitro fertilization cycle. 2008. 90 (3): 613-8.
145. S. A. Krieg,​ M. B. Henne and L. M. Westphal. Obstetric outcomes in donor oocyte pregnancies compared with advanced maternal age in in vitro fertilization pregnancies. 2008. 90 (1): 65-70.
146. A. Pinborg,​ O. Lidegaard,​ N. l. C. Freiesleben and A. N. Andersen. Vanishing twins: a predictor of small-for-gestational age in IVF singletons. 2007. 22 (10): 2707-14.
147. D. A. Keegan,​ L. C. Krey,​ H.-C. Chang and N. Noyes. Increased risk of pregnancy-induced hypertension in young recipients of donated oocytes. 2007. 87 (4): 776-81.
148. D. Hvidtjorn, J. Grove, D. Schendel, C. Svaerke, L. A. Schieve, P. Uldall, E. Ernst, B. Jacobsson and P. Thorsen. Multiplicity and early gestational age contribute to an increased risk of cerebral palsy from assisted conception: a population-based cohort study. 2010. 25 (8): 2115-23.
149. A. Hourvitz,​ S. Pri-Paz,​ J. Dor and D. S. Seidman. Neonatal and obstetric outcome of pregnancies conceived by ICSI or IVF. 2005. 11 (4): 469-75.
150. M. Bonduelle,​ I. Liebaers,​ V. Deketelaere,​ M.-P. Derde,​ M. Camus,​ P. Devroey and A. Van Steirteghem. Neonatal data on a cohort of 2889 infants born after ICSI (1991-1999) and of 2995 infants born after IVF (1983-1999). 2002. 17 (3): 671-94.
151. R. M. Kermani,​ B. Allahverdi,​ H. Gourabi,​ J. Koohpayezade,​ M. R. Nateghi and S. Dadashloo. Perinatal outcomes of newborn infants conceived by assisted reproductive techniques in Royan Institute. 2009. 3 (2): 62-65.
152. G. D. Palermo,​ Q. V. Neri,​ T. Takeuchi,​ J. Squires,​ F. Moy and Z. Rosenwaks. Genetic and epigenetic characteristics of ICSI children. 2008. 17 (6): 820-833.
153. K. Chung,​ C. Coutifaris,​ R. Chalian,​ K. Lin,​ S. J. Ratcliffe,​ A. J. Castelbaum,​ M. F. Freedman and K. T. Barnhart. Factors influencing adverse perinatal outcomes in pregnancies achieved through use of in vitro fertilization. 2006. 86 (6): 1634-1641.
154. X. Xiong,​ R. P. Dickey,​ P. Buekens,​ J. G. Shaffer and G. Pridjian. Use of Intracytoplasmic Sperm Injection and Birth Outcomes in Women Conceiving through In Vitro Fertilization. 2017. 31 (2): 108-115.
155. W. Ombelet,​ G. Martens and L. Bruckers. Pregnant after assisted reproduction: a risk pregnancy is born! 18-years perinatal outcome results from a population-based registry in Flanders,​ Belgium. 2016. 8 (4): 193-204.
156. J.-B. Qin,​ X.-Q. Sheng,​ H. Wang,​ G.-C. Chen,​ J. Yang,​ H. Yu and T.-B. Yang. Worldwide prevalence of adverse pregnancy outcomes associated with in vitro fertilization/intracytoplasmic sperm injection among multiple births: a systematic review and meta-analysis based on cohort studies. 2017. 295 (3): 577-597.
157. M. Wang,​ C. Hao,​ H. Bao,​ X. Huang,​ Z. Liu,​ W. Zhang and F. Li. Effect of elevated estradiol levels on the hCG administration day on IVF pregnancy and birth outcomes in the long GnRH-agonist protocol: analysis of 3393 cycles. 2017. 295 (2): 407-414.
158. S. K. Sunkara,​ B. Antonisamy,​ H. Y. Selliah and M. S. Kamath. Pre-term birth and low birth weight following preimplantation genetic diagnosis: analysis of 88 010 singleton live births following PGD and IVF cycles. 2017. 32 (2): 432-438.
159. B. Valenzuela-Alcaraz,​ F. Crispi,​ D. Manau,​ M. Cruz-Lemini,​ A. Borras,​ J. Balasch and E. Gratacos. Differential effect of mode of conception and infertility treatment on fetal growth and prematurity. 2016. 29 (23): 3879-84.
160. L. Zhu,​ Y. Zhang,​ Y. Liu,​ R. Zhang,​ Y. Wu,​ Y. Huang,​ F. Liu,​ M. Li,​ S. Sun,​ L. Xing,​ Y. Zhu,​ Y. Chen,​ L. Xu,​ L. Zhou,​ H. Huang and D. Zhang. Maternal and Live-birth Outcomes of Pregnancies following Assisted Reproductive Technology: A Retrospective Cohort Study. 2016. 6: 35141.
161. A. Maheshwari,​ E. A. Raja and S. Bhattacharya. Obstetric and perinatal outcomes after either fresh or thawed frozen embryo transfer: an analysis of 112,​432 singleton pregnancies recorded in the Human Fertilisation and Embryology Authority anonymized dataset. 2016. 106 (7): 1703-1708.
162. J. F. Kawwass,​ A. D. Kulkarni,​ H. S. Hipp,​ S. Crawford,​ D. M. Kissin and D. J. Jamieson. Extremities of body mass index and their association with pregnancy outcomes in women undergoing in vitro fertilization in the United States. 2016. 106 (7): 1742-1750.
163. X. Xiong,​ R. P. Dickey,​ G. Pridjian and P. Buekens. Maternal age and preterm births in singleton and twin pregnancies conceived by in vitro fertilisation in the United States. 2015. 29 (1): 22-30.
164. L. Xiang,​ Z. Wei,​ J. Wu,​ P. Zhou,​ H. Xiang and Y. Cao. Clinical significance of first-trimester intrauterine haematomas detected in pregnancies achieved by IVF-embryo transfer. 2014. 29 (4): .445-51.
165. S. Korosec,​ H. Ban Frangez,​ I. Verdenik,​ U. Kladnik,​ V. Kotar,​ I. Virant-Klun and E. Vrtacnik Bokal. Singleton pregnancy outcomes after in vitro fertilization with fresh or frozen-thawed embryo transfer and incidence of placenta praevia. 2014.
166. J. Chai,​ T. W. Y. Yeung,​ V. C. Y. Lee,​ R. H. W. Li,​ E. Y. L. Lau,​ W. S. B. Yeung,​ P. C. Ho and E. H. Y. Ng. Live birth rate,​ multiple pregnancy rate,​ and obstetric outcomes of elective single and double embryo transfers: Hong Kong experience. 2014. 20 (2): 102-6.
167. M. M. J. van Heesch,​ J. L. H. Evers,​ J. C. M. Dumoulin,​ M. A. H. B. M. van der Hoeven,​ C. E. M. van Beijsterveldt,​ G. J. Bonsel,​ R. H. M. Dykgraaf,​ J. B. van Goudoever,​ C. Koopman-Esseboom,​ W. L. D. M. Nelen,​ K. Steiner,​ P. Tamminga,​ N. Tonch,​ P. van Zonneveld and C. D. Dirksen. A comparison of perinatal outcomes in singletons and multiples born after in vitro fertilization or intracytoplasmic sperm injection stratified for neonatal risk criteria. 2014. 93 (3): 277-86.
168. K. Nouri,​ J. Ott,​ L. Stoegbauer,​ D. Pietrowski,​ S. Frantal and K. Walch. Obstetric and perinatal outcomes in IVF versus ICSI-conceived pregnancies at a tertiary care center--a pilot study. 2013. 11: 84.
169. R. P. Dickey,​ X. Xiong,​ Y. Xie,​ R. E. Gee and G. Pridjian. Effect of maternal height and weight on risk for preterm singleton and twin births resulting from IVF in the United States,​ 2008-2010. 2013. 209 (4): 349.e1-6.
170. S. Y. Liu,​ B. Teng,​ J. Fu,​ X. Li,​ Y. Zheng and X. X. Sun. Obstetric and neonatal outcomes after transfer of vitrified early cleavage embryos. 2013. 28 (8): 2093-100.
171. S. S. Malchau,​ A. Loft,​ E. C. Larsen,​ A.-K. Aaris Henningsen,​ S. Rasmussen,​ A. N. Andersen and A. Pinborg. Perinatal outcomes in 375 children born after oocyte donation: a Danish national cohort study. 2013. 99 (6): 1637-43.
172. J. Mangot-Bertrand,​ F. Fenollar,​ F. Bretelle,​ M. Gamerre,​ D. Raoult and B. Courbiere. Molecular diagnosis of bacterial vaginosis: impact on IVF outcome. 2013. 32 (4): 535-41.
173. A. Nakashima,​ R. Araki,​ H. Tani,​ O. Ishihara,​ A. Kuwahara,​ M. Irahara,​ Y. Yoshimura,​ T. Kuramoto,​ H. Saito,​ A. Nakaza and T. Sakumoto. Implications of assisted reproductive technologies on term singleton birth weight: an analysis of 25,​777 children in the national assisted reproduction registry of Japan. 2013. 99 (2): 450-5.
174. D. L. Herbert,​ J. C. Lucke and A. J. Dobson. Birth outcomes after spontaneous or assisted conception among infertile Australian women aged 28 to 36 years: a prospective,​ population-based study. 2012. 97 (3): 630-8.
175. D. A. Lawlor and S. M. Nelson. Effect of age on decisions about the numbers of embryos to transfer in assisted conception: a prospective study. 2012. 379 (9815): 521-7.
176. R. Grady,​ N. Alavi,​ R. Vale,​ M. Khandwala and S. D. McDonald. Elective single embryo transfer and perinatal outcomes: a systematic review and meta-analysis. 2012. 97 (2): 324-31.
177. S. Kansal Kalra 2011
178. S. Kansal Kalra,​ S. J. Ratcliffe,​ L. Milman,​ C. R. Gracia,​ C. Coutifaris and K. T. Barnhart. Perinatal morbidity after in vitro fertilization is lower with frozen embryo transfer. 2011. 95 (2): 548-53.
179. D. J. McLernon,​ K. Harrild,​ C. Bergh,​ M. J. Davies,​ D. de Neubourg,​ J. C. M. Dumoulin,​ J. Gerris,​ J. A. M. Kremer,​ H. Martikainen,​ B. W. Mol,​ R. J. Norman,​ A. Thurin-Kjellberg,​ A. Tiitinen,​ A. P. A. van Montfoort,​ A. M. van Peperstraten,​ E. Van Royen and S. Bhattacharya. Clinical effectiveness of elective single versus double embryo transfer: meta-analysis of individual patient data from randomised trials. 2010. 341: 6945.
180. P. C. Klatsky,​ S. S. Delaney,​ A. B. Caughey,​ N. D. Tran,​ G. L. Schattman and Z. Rosenwaks. The role of embryonic origin in preeclampsia: a comparison of autologous in vitro fertilization and ovum donor pregnancies. 2010. 116 (6): 1387-92.
181. S. Pelkonen,​ R. Koivunen,​ M. Gissler,​ S. Nuojua-Huttunen,​ A. M. Suikkari,​ C. Hyden-Granskog,​ H. Martikainen,​ A. Tiitinen and A. L. Hartikainen. Perinatal outcome of children born after frozen and fresh embryo transfer: the Finnish cohort study 1995-2006. 2010. 25 (4): 914-23.
182. B. Luke,​ M. B. Brown,​ D. E. Morbeck,​ S. B. Hudson,​ C. C. Coddington,​ 3rd and J. E. Stern. Factors associated with ovarian hyperstimulation syndrome (OHSS) and its effect on assisted reproductive technology (ART) treatment and outcome. 2010. 94 (4): 1399-404.
183. B. Luke, M. B. Brown, J. E. Stern, S. A. Missmer, V. Y. Fujimoto and R. E. Leach. Racial and ethnic disparities in Assisted Reproductive Technology (ART) pregnancy rates and obstetric outcomes within BMI groups. 2010. 17 (3 SUPPL. 1): 289A.
184. V. M. Savasi,​ L. Mandia,​ A. Laoreti and I. Cetin. Maternal and fetal outcomes in oocyte donation pregnancies. 2016. 22 (5): 620-633.
185. L. Yin,​ F. Hang,​ L. J. Gu,​ B. Xu,​ D. Ma and G. J. Zhu. Analysis of birth defects among children 3 years after conception through assisted reproductive technology in China. 2013. 97 (11): 744-749.
186. L. J. Salomon,​ S. Hourrier,​ R. Fanchin,​ Y. Ville and P. Rozenberg. Is first-trimester crown-rump length associated with birthweight?. 2011. 118 (10): 1223-1228.
187. B. Almog,​ I. Levin,​ I. Wagman,​ R. Kapustiansky,​ J. B. Lessing,​ A. Amit and F. Azem. Adverse obstetric outcome for the vanishing twin syndrome. 2010. 20 (2): 256-260.
188. A. Berkovitz,​ A. Hershko-Klement and M. Fejgin. Nulliparity,​ fertility treatments and twins: a time for rethinking. 2010. 93 (6): 1957-60.
189. A. Ciavattini,​ P. Stortoni,​ F. Mancioli,​ D. Puglia,​ A. L. Tranquilli and C. A. Liverani. The impact of loop electrosurgical excision procedure (LEEP) for CIN 2,​3 on spontaneous preterm delivery in twin pregnancies by assisted reproductive technique: Preliminary data. 2014. 27 (11): 1169-1171.
190. A. Manzur,​ M. P. Goldsman,​ S. C. Stone,​ J. L. Frederick,​ J. P. Balmaceda and R. H. Asch. Outcome of triplet pregnancies after assisted reproductive techniques: how frequent are the vanishing embryos?. 1995. 63 (2): 252-7.
191. M. I. Evans, R. L. Berkowitz, R. J. Wapner, R. J. Carpenter, J. D. Goldberg, M. A. Ayoub, J. Horenstein, M. Dommergues, B. Brambati, K. H. Nicolaides, W. Holzgreve and I. E. Timor-Tritsch. Improvement in outcomes of multifetal pregnancy reduction with increased experience. 2001. 184 (2): 97-103.
192. Y. Mehlman. Multi-fetal pregnancy reduction. 1994. No. 27: 35-68.
193. H. H. Kanhai, M. de Haan, L. A. van Zanten, C. Geerinck-Vercammen, H. M. van der Ploeg and J. B. Gravenhorst. Follow-up of pregnancies, infants, and families after multifetal pregnancy reduction. 1994. 62 (5): 955-9.
194. D. Delafontaine, S. Mugniot-bellamy, S. Simeon and M. N. Menard. [Selective embryonic or fetal reduction by surgical transvaginal ultrasonography]. 1991. 19 (6): 473-81.
195. P. D. Dimcev. Selective embrioreduction in multifetal pregnancies following art procedures. 2012. 25: 57-58.
196. B. Romero Guadix, A. Clavero Gilabert, S. Zamora, M. C. Gonzalvo, J. A. Castilla Alcala and L. Martinez Navarro. Reduction of multiple pregnancy rate when we transfer a single embryo in IVF cycles. 2010. 23: 472.
197. R. V. Haning Jr, D. B. Seifer, C. A. Wheeler, G. N. Frishman, H. Silver and D. J. Pierce. Effects of fetal number and multifetal reduction on length of in vitro fertilization pregnancies. 1996. 87 (6): 964-968.
198. P. Patkos. Embryonic reduction, selective termination. 2003. 3 (4): 290-293.
199. A. Ayres and T. R. B. Johnson. Management of multiple pregnancy: prenatal care--part II. 2005. 60 (8): 538-49.
200. I. Blickstein and L. G. Keith. Multiple gestations. 2005. 32 (1): xiii-xiv.
201. I. Blickstein and L. G. Keith. Iatrogenic multiple pregnancy. 2002. 7 (3): 169-76.
202. R. L. Bergmann, R. Richter, K. E. Bergmann and J. W. Dudenhausen. The prevalence of preterm deliveries in Berlin has not changed over 7 years: the impact of multiple births. 2004. 32 (3): 234-9.
203. G. B. White and S. R. Leuthner. Infertility treatment and neonatal care: the ethical obligation to transcend specialty practice in the interest of reducing multiple births. 2001. 12 (3): 223-30.
204. Y. R. Lien, C. H. Jehng, J. L. Hwang, H. J. Jou, A. L. Jenh and G. T. Tsaur. First-trimester selective termination in multiple gestation by transvaginal ultrasound-guided intrathoracic injection of potassium chloride. 1994. 39 (2): 90-4.
205. P. Doyle. The outcome of multiple pregnancy. 1996. 11 Suppl 4: 110-20.
206. R. M. Zaner, F. H. Boehm and G. A. Hill. Selective termination in multiple pregnancies: ethical considerations. 1990. 54 (2): 203-5.
207. S. Vaksmann, P. Bouchart, P. Patey-Savatier, C. Maunoury-Lefebvre, D. Vinatier and J. C. Monnier. [Multiple pregnancies. II. Epidemiology, clinical aspects]. 1990. 19 (4): 383-94.
208. M. Milicevic and S. Potic. Assessing the risk of cerebral palsy in children born after assisted conception - The role of multiple pregnancy and preterm delivery. 2015. 32 (2): 101-113.
209. A. Perez-Munuzuri, A. Iglesias Deus, J. R. Cervilla, A. Bana Souto, A. Urisarri Ruiz de Cortazar and M. L. Couce Pico. The effect of in vitro fetilization on multiple gestation and preterm birth. Assessment in 55.725 deliveries. 2014. 27: 428.
210. V. Bitsadze, N. Makatsariya, R. Abramian and Z. Gadaeva. Prevention of reproductive losses in women with thrombophilia and multiple pregnancy after IVF. 2014. 27: 31-32.
211. J. Milliez and B. Dickens. Ethical aspects of multiple pregnancy. 2009. 3 (1): 41-46.
212. Anonymous. ACOG educational bulletin. Special problems of multiple gestation. Number 253, November 1998 (Replaces Number 131, August 1989). American College of Obstetricians and Gynecologists. 1999. 64 (3): 323-33.
213. Anonymous. Multiple pregnancy associated with infertility therapy. 2004. 82: (SUPPL. 1): S153-S157.
214. E. R. Norwitz. Multiple pregnancy: Trends past, present, and future. 1998. 9 (3): 351-369.
215. E. Mueller-Heubach. Complications of multiple gestation. 1984. 27 (4): 1003-1013.

## Did not identify frozen or Fresh embryo transfer

1. Carbillon L, Gronier H, Cedrin-Durnerin I, Pharisien I, Nguyen MT, Valensi P, Cosson E. The impact of ovulation induction and ovarian stimulation on the risk of pregnancy-induced hypertension and on neonatal outcomes: A case/control study. European Journal of Obstetrics & Gynecology and Reproductive Biology. 2017. 217:137-43.
2. Qin J, Sheng X, Wu D, Gao S, You Y, Yang T, Wang H. Adverse obstetric outcomes associated with in vitro fertilization in singleton pregnancies: a prospective cohort study. Reproductive Sciences. 2017. (4):595-608.
3. Liu J, Linara E, Zhao W, Ma H, Ahuja K, Wang J. Neonatal and obstetric outcomes of in vitro fertilization (IVF) and natural conception at a Chinese reproductive unit. Clin Exp Obstet Gynecol. 2015. 42(4):452-6.
4. Ochsenkühn R, Strowitzki T, Gurtner M, Strauss A, Schulze A, Hepp H, Hillemanns P. Pregnancy complications, obstetric risks, and neonatal outcome in singleton and twin pregnancies after GIFT and IVF. Archives of gynecology and obstetrics. 2003 Oct 1;268(4):256-61.
5. N. Dayan, A. Lanes, M. C. Walker, K. A. Spitzer and C. A. Laskin. Effect of chronic hypertension on assisted pregnancy outcomes: a population-based study in Ontario, Canada. 2016. 105 (4): 1003-9.
6. Frankenthal D, Hirsh-Yechezkel G, Boyko V, Orvieto R, Ron-El R, Lerner-Geva L, Farhi A. The effect of body mass index (BMI) and gestational weight gain on adverse obstetrical outcomes in pregnancies following assisted reproductive technology as compared to spontaneously conceived pregnancies. Obesity research & clinical practice. 2018. (Israel)
7. Middelburg KJ, Haadsma ML, Heineman MJ, Bos AF, Hadders-Algra M. Ovarian hyperstimulation and the in vitro fertilization procedure do not influence early neuromotor development; a history of subfertility does. Fertility and sterility. 2012. 27(5):1343-50. (Netherland)
8. K. J. Middelburg,​ M. L. Haadsma,​ M. J. Heineman,​ A. F. Bos and M. Hadders-Algra. Ovarian hyperstimulation and the in vitro fertilization procedure do not influence early neuromotor development; a history of subfertility does. 2010. 93 (2): 544-53. (Netherland)
9. Wang AY, Chughtai AA, Lui K, Sullivan EA. Morbidity and mortality among very preterm singletons following fertility treatment in Australia and New Zealand, a population cohort study. BMC pregnancy and childbirth. 2017. 17(1):50.
10. M. Hayashi, A. Nakai, S. Satoh and Y. Matsuda. Adverse obstetric and perinatal outcomes of singleton pregnancies may be related to maternal factors associated with infertility rather than the type of assisted reproductive technology procedure used. 2012. 98 (4): 922-8.
11. M. Toshimitsu, T. Nagamatsu, T. Nagasaka, Y. Iwasawa-Kawai, A. Komatsu, T. Yamashita, Y. Osuga and T. Fujii. Increased risk of pregnancy-induced hypertension and operative delivery after conception induced by in vitro fertilization/intracytoplasmic sperm injection in women aged 40 years and older. 2014. 102 (4): 1065-1070.e1.
12. H. Li, H.-L. Zhu, X.-H. Chang, Y. Li, Y. Wang, J. Guan and H. Cui. Effects of Previous Laparoscopic Surgical Diagnosis of Endometriosis on Pregnancy Outcomes. 2017. 130 (4): 428-433.
13. A. Farhi,​ B. Reichman,​ V. Boyko,​ A. Hourvitz,​ R. Ron-El and L. Lerner-Geva. Maternal and neonatal health outcomes following assisted reproduction. 2013. 26 (5): 454-461.
14. S. Raisanen, K. Randell, H. S. Nielsen, M. Gissler, M. R. Kramer, R. Klemetti and S. Heinonen. Socioeconomic status affects the prevalence, but not the perinatal outcomes, of in vitro fertilization pregnancies. 2013. 28 (11): 3118-25.
15. Y. Zhong,​ R. Bradshaw,​ A. P. Stanley and A. O. Odibo. The impact of assisted reproductive technology on the association between first-trimester pregnancy-associated plasma protein a and human chorionic gonadotropin and adverse pregnancy outcomes. 2011. 28 (5): 347-54.
16. M. J. Pelinck,​ M. Hadders-Algra,​ M. L. Haadsma,​ W. L. Nijhuis,​ S. M. Kiewiet,​ A. Hoek,​ M. J. Heineman and K. J. Middelburg. Is the birthweight of singletons born after IVF reduced by ovarian stimulation or by IVF laboratory procedures?. 2010. 21 (2): 245-251.
17. Chughtai AA, Wang AY, Hilder L, Li Z, Lui K, Farquhar C, Sullivan EA. Gestational age-specific perinatal mortality rates for assisted reproductive technology (ART) and other births. Human reproduction (Oxford, England). 2018. 33(2):320.
18. Dunietz GL, Holzman C, Zhang Y, Li C, Todem D, Boulet SL, McKane P, Kissin DM, Copeland G, Bernson D, Diamond MP. Assisted reproduction and risk of preterm birth in singletons by infertility diagnoses and treatment modalities: a population-based study. Journal of assisted reproduction and genetics. 2017. 34(11):1529-35.
19. Eldar-Geva T, Srebnik N, Altarescu G, Varshaver I, Brooks B, Levy-Lahad E, Bromiker R, Schimmel MS. Neonatal outcome after preimplantation genetic diagnosis. Fertility and sterility. 2014. 102(4):1016-21.
20. Frankenthal D, Hirsh-Yechezkel G, Boyko V, Orvieto R, Ron-El R, Lerner-Geva L, Farhi A. The effect of body mass index (BMI) and gestational weight gain on adverse obstetrical outcomes in pregnancies following assisted reproductive technology as compared to spontaneously conceived pregnancies. Obesity research & clinical practice. 2018.
21. Fujii M, Matsuoka R, Bergel E, van der Poel S, Okai T. Perinatal risk in singleton pregnancies after in vitro fertilization. Fertility and sterility. 2010. 94(6):2113-7.
22. Hansen M, Kurinczuk JJ, Bower C, Webb S. The risk of major birth defects after intracytoplasmic sperm injection and in vitro fertilization. New England Journal of Medicine. 2002 Mar 7;346(10):725-30.
23. Harlev A, Walfisch A, Oran E, Har‐Vardi I, Friger M, Lunenfeld E, Levitas E. The effect of fertility treatment on adverse perinatal outcomes in women aged at least 40 years. International Journal of Gynecology & Obstetrics. 2018. 140(1):98-104.
24. Kalayci H, Ozdemir H, Alkas D, Cok T, Tarim E. Is primiparity a risk factor for advanced maternal age pregnancies?. The Journal Of Maternal-fetal & Neonatal Medicine. 2017. 30(11):1283-7.
25. Kelley-Quon LI, Tseng CH, Janzen C, Shew SB. Congenital malformations associated with assisted reproductive technology: A California statewide analysis. Journal of pediatric surgery. 2013. 48(6):1218-24.
26. Kouhkan A, Khamseh ME, Pirjani R, Moini A, Arabipoor A, Maroufizadeh S, Hosseini R, Baradaran HR. Obstetric and perinatal outcomes of singleton pregnancies conceived via assisted reproductive technology complicated by gestational diabetes mellitus: a prospective cohort study. BMC pregnancy and childbirth. 2018. 18(1):495.
27. Lucovnik M, Blickstein I, Mirkovic T, Verdenik I, Bricelj K, Simic MV, Tul N, Bregar AT. Effect of pre-gravid body mass index on outcomes of pregnancies following in vitro fertilization. Journal of assisted reproduction and genetics. 2018. 35(7):1309-15.
28. Luke B, Gopal D, Cabral H, Stern JE, Diop H. Pregnancy, birth, and infant outcomes by maternal fertility status: the Massachusetts Outcomes Study of Assisted Reproductive Technology. American journal of obstetrics and gynecology. 2017. 217(3):327-e1.
29. Olivennes F, Rufat P, Andre B, Pourade A, Quiros MC, Frydman R. Pregnancy: The increased risk of complication observed in singleton pregnancies resulting from in-vitro fertilization (IVF) does not seem to be related to the IVF method itself. Human Reproduction. 1993. 8(8):1297-300.
30. Olson CK, Keppler-Noreuil KM, Romitti PA, Budelier WT, Ryan G, Sparks AE, Van Voorhis BJ. In vitro fertilization is associated with an increase in major birth defects. Fertility and sterility. 2005. 84(5):1308-15.
31. Willem O, Karen P, De Sutter P, Jan G, Eugene B, Guy M, Gunther R, Paul D, Geert M, Wilfried G. Perinatal outcome of ICSI pregnancies compared with a matched group of natural conception pregnancies in Flande
32. Reubinoff BE, Samueloff A, Ben-Haim M, Friedler S, Schenker JG, Lewin A. Is the obstetric outcome of in vitro fertilized singleton gestations different from natural ones? A controlled study. Fertility and sterility. 1997. 67(6):1077-83.
33. T. Shevell, F. D. Malone, J. Vidaver, T. F. Porter, D. A. Luthy, C. H. Comstock, G. D. Hankins, K. Eddleman, S. Dolan, L. Dugoff, S. Craigo, I. E. Timor, S. R. Carr, H. M. Wolfe, D. W. Bianchi and M. E. D'Alton. Assisted reproductive technology and pregnancy outcome. 2005. 106 (5 Pt 1): 1039-45.
34. Tan SL, Doyle P, Campbell S, Beral V, Rizk B, Brinsden P, Mason B, Edwards RG. Obstetric outcome of in vitro fertilization pregnancies compared with normally conceived pregnancies. American Journal of Obstetrics and Gynecology. 1992. 167(3):778-84.
35. Tomic V, Tomic J. Neonatal outcome of IVF singletons versus naturally conceived in women aged 35 years and over. Archives of gynecology and obstetrics. 2011. 284(6):1411-6.
36. Wang YA, Sullivan EA, Black D, Dean J, Bryant J, Chapman M. Preterm birth and low birth weight after assisted reproductive technology-related pregnancy in Australia between 1996 and 2000. Fertility and sterility. 2005. 83(6):1650-8.
37. M. Knoester,​ F. M. Helmerhorst,​ J. P. Vandenbroucke,​ L. A. J. van der Westerlaken,​ F. J. Walther,​ S. Veen and P. Leiden Artificial Reproductive Techniques Follow-up. Perinatal outcome,​ health,​ growth,​ and medical care utilization of 5- to 8-year-old intracytoplasmic sperm injection singletons. 2008. 89 (5): 1133-46.
38. S. Ensing, A. Abu-Hanna, T. J. Roseboom, S. Repping, F. van der Veen, B. W. J. Mol and A. C. J. Ravelli. Risk of poor neonatal outcome at term after medically assisted reproduction: a propensity score-matched study. 2015. 104 (2): 384-90.e1.

**Without control group**

1. K. R. Brown, N. Onwubalili, A. J. Fechner, S. K. Jindal, L. T. Goldsmith and P. G. McGovern. Single embryo transfer (SET) during in vitro fertilization (IVF) does not significantly reduce the risk of preterm delivery. 2012. 19 (3 SUPPL. 1): 127A.
2. W. Liu, X. F. Li, W. Y. Xi, L. Tan, L. Q. Fan and G. X. Lu. The follow-up study of pregnancy and neonates outcomes of 10648 AID cycles. 2011. 26: i277-i278.
3. N. S. Uysal, C. Gulumser and F. B. Yanik. Maternal and perinatal characteristics of small-for-gestational-age newborns: Ten-year experience of a single center. 2017. 18 (2): 90-95.
4. M. S. el-Ashnehi, M. E. Ibrahim, H. el-Tamamy and D. Murad. Pregnancy outcome after female infertility in Kuwait. Comparison of medical and surgical treatment. 1990. 69 (5): 389-92.
5. F. Olivennes, R. Frydman, P. Rufat, J. de Mouzon and M. Dehan. How to halve the prematurity rates of in vitro fertilization pregnancies in 4 days. 1992. 9 (4): 406-7.
6. Anonymous. In-vitro fertilization pregnancies in Australia and New Zealand, 1979-1985. Australian In-Vitro Fertilization Collaborative Group. 1988. 148 (9): 429-6.
7. D. Tabs,​ T. Vejnovic and N. Radunovic. Preterm and premature rupture of membranes in pregnancies after in vitro fertilization. 2005. 58 (7-8): 375-9.
8. Carassou-Maillan,​ J. L. Pouly,​ A. Mulliez,​ L. Dejou-Bouillet,​ A. S. Gremeau,​ F. Brugnon,​ L. Janny and M. Canis. [Adverse pregnancy outcomes after Assisted Reproduction Technology in women with endometriosis]. 2014. 42 (4): 210-5.
9. J. P. Relier. Results for treating hypofertility: The neonatal risks after IVF. 1992. 20 (2): 136-141.
10. Sunkara SK, Antonisamy B, Selliah HY, Kamath MS. Pre-term birth and low birth weight following preimplantation genetic diagnosis: analysis of 88 010 singleton live births following PGD and IVF cycles. Human Reproduction. 2017. 32(2):432-8.
11. C. Wittemer, N. Machev and S. Viville. [Follow-up of children born by ICSI]. 2004. 20 (3): 352-6.
12. Pereira N, Elias RT, Christos PJ, Petrini AC, Hancock K, Lekovich JP, Rosenwaks Z. Supraphysiologic estradiol is an independent predictor of low birth weight in full-term singletons born after fresh embryo transfer. Human Reproduction. 2017. 32(7):1410-7.
13. R. P. Dickey, X. Xiong, G. Pridjian and M. C. Klempel. Singleton birthweight by gestational age following in vitro fertilization in the United States. 2016. 214 (1):101.e1-101.e13
14. K. Y. Cha, H. M. Chung, D. R. Lee, H. Kwon, M. K. Chung, L. S. Park, D. H. Choi and T. K. Yoon. Obstetric outcome of patients with polycystic ovary syndrome treated by in vitro maturation and in vitro fertilization-embryo transfer. 2005. 83 (5): 1461-5.
15. Marconi N, Raja EA, Bhattacharya S, Maheshwari A. Birthweight and gestational age following blastocyst transfer compared to cleavage stage embryo transfer: an analysis of 68,042 singleton births. Human Reproduction. 2018. 33:32-32.
16. I. Grefenstette, D. Royere, C. Barthelemy, M. J. Tharanne and J. Lansac. [Outcome of 470 pregnancies after artificial insemination with frozen sperm]. 1990. 19 (6): 737-44.
17. Kamath MS, Antonisamy B, Selliah HY, Sunkara SK. Does transfer of multiple embryos affect perinatal outcomes of the resulting singleton live births? Analysis of 113 784 singleton live births following ART. Human Reproduction. 2018. 33: 33-33.
18. E. Behl, O. Abuzeid, T. Buikema, G. Kazzi, J. Murphy, M. Abuzeid and M. Ashraf. Incidence of preterm birth with primary infertility, normal uterine cavity and subsequent singleton gestation conceived spontaneously, via intrauterine insemination, or in vitro fertilization. 2015. 212 (1 SUPPL. 1): S140.
19. M. C. Tio, J. Ten, A. Rodriguez-Arnedo, J. Guerrero, B. Moliner, F. Sellers, J. Llacer and R. Bernabeu. Effect of embryo freezing process on birth weight. 2015. 30: i253.
20. H. Burks, J. Bruno-Gaston, F. Stanczyk, K. Bendikson, R. Paulson and K. Chung. Is endometrial preparation during frozen embryo transfer associated with adverse obstetrical outcomes?. 2015. 103 (2 SUPPL. 1): e16.
21. Lin R, Feng G, Shu J, Zhang B, Zhou H, Gan X, Wang C, Chen H. Blastocoele re‐expansion time in vitrified–warmed cycles is a strong predictor of clinical pregnancy outcome. Journal of Obstetrics and Gynaecology Research. 2017. (4):689-95.
22. M. C. Andrews, S. J. Muasher, D. L. Levy, H. W. Jones, Jr., J. E. Garcia, Z. Rosenwaks, G. S. Jones and A. A. Acosta. An analysis of the obstetric outcome of 125 consecutive pregnancies conceived in vitro and resulting in 100 deliveries. 1986. 154 (4): 848-54.
23. Iaconelli Jr A, Zanetti BF, Braga DP, Setti AS, Figueira RC, Borges Jr E. Endometrial thickness and oocyte quality affect perinatal outcomes in intracytoplasmic sperm injection cycles. Human Reproduction 2018. 33:336-337.
24. Marozio L, Picardo E, Filippini C, Mainolfi E, Berchialla P, Cavallo F, Tancredi A, Benedetto C. Maternal age over 40 years and pregnancy outcome: a hospital-based survey. The journal of maternal-fetal & neonatal medicine: the official journal of the European Association of Perinatal Medicine, the Federation of Asia and Oceania Perinatal Societies, the International Society of Perinatal Obstetricians. 2017. 1-7.
25. Rendtorff R, Hinkson L, Kiver V, Dröge LA, Henrich W. Pregnancies in women aged 45 years and older–a 10-year retrospective analysis in Berlin. Geburtshilfe und Frauenheilkunde. 2017. 77(03):268-75.
26. Insua MF, Cobo AC, Larreategui Z, Ferrando M, Serra V, Meseguer M. Obstetric and perinatal outcomes of pregnancies conceived with embryos cultured in a time-lapse monitoring system. Fertility and sterility. 2017. 108(3):498-504.
27. Khatibi A, Andersen AM, Gissler M, Morken NH, Jacobsson B. Obstetric and neonatal outcome in women aged 50 years and up: A collaborative, Nordic population-based study. European Journal of Obstetrics & Gynecology and Reproductive Biology. 2018. 224:17-20.
28. W. S. Lai, S. S. F. Yung and E. H. Y. Ng. Endometrial vascularity is lower in pregnancies with pregnancy-induced hypertension or small-for-gestational-age fetus in live birth after in-vitro fertilization. 2014. 44 (4): 455-60.
29. Enache, B. Marinescu and T. Enache. Pregnancy outcome after IVF. 2012. 60 (1): 57-60.
30. U. B. Wennerholm,​ P. O. Janson,​ M. Wennergren and I. Kjellmer. Pregnancy complications and short-term follow-up of infants born after in vitro fertilization and embryo transfer (IVF/ET). 1991. 70 (7-8): 565-73.
31. G. Oron,​ W.-Y. Son,​ W. Buckett,​ T. Tulandi and H. Holzer. The association between embryo quality and perinatal outcome of singletons born after single embryo transfers: a pilot study. 2014. 29 (7): 1444-51.
32. Haddad,​ F. Abirached,​ C. Louis-Sylvestre,​ J. Le Blond,​ B. J. Paniel and J. R. Zorn. Predictive value of early human chorionic gonadotrophin serum profiles for fetal growth retardation. 1999. 14 (11): 2872-5.
33. G. D. t. Royster,​ K. Krishnamoorthy,​ J. M. Csokmay,​ B. J. Yauger,​ R. J. Chason,​ A. H. DeCherney,​ E. F. Wolff and M. J. Hill. Are intracytoplasmic sperm injection and high serum estradiol compounding risk factors for adverse obstetric outcomes in assisted reproductive technology?. 2016. 106 (2): 363-370.e3.
34. S. Marcus,​ T. al-Shawaf and P. Brinsden. The obstetric outcome of in vitro fertilization and embryo transfer in women with congenital uterine malformation. 1996. 175 (1): .85-9.
35. P. Doyle,​ V. Beral and N. Maconochie. Preterm delivery,​ low birthweight and small-for-gestational-age in liveborn singleton babies resulting from in-vitro fertilization. 1992. 7 (3): 425-8.
36. U. B. Wennerholm,​ P. O. Janson,​ M. Wennergren and I. Kjellmer. Pregnancy complications and short-term follow-up of infants born after in vitro fertilization and embryo transfer (IVF/ET). 1991. 70 (7-8): 565-73.
37. Li Y, Li G, Broadwell CE, Jones JM, Boehnlein L, Stanic-Kostic A. Intracytoplasmic sperm injection and low body mass index independently associate with low fetal birth weight in singleton art cycles. Fertility and Sterility. 2017. 108(3):e347.
38. N. Pereira,​ R. T. Elias,​ P. J. Christos,​ A. C. Petrini,​ K. Hancock,​ J. P. Lekovich and Z. Rosenwaks. Supraphysiologic estradiol is an independent predictor of low birth weight in full-term singletons born after fresh embryo transfer. 2017. 1-8.
39. W. Zhou,​ Y. Zhuang,​ Y. Pan and F. Xia. Effects and safety of GnRH-a as a luteal support in women undertaking assisted reproductive technology procedures: follow-up results for pregnancy,​ delivery,​ and neonates. 2017. 295 (5): 1269-1275.
40. T. Kanninen, S. Spandorfer, D. Goldschlag and S. Witkin. Early luteal serum levels of biomarkers are predictive of preterm delivery (PTD) in singleton IVF pregnancies. 2014. 102 (3 SUPPL. 1): e60-e61N. Pereira,​ T. Cozzubbo,​ S. Cheung,​ Z. Rosenwaks,​ G. D. Palermo and Q. V. Neri. Identifying Maternal Constraints on Fetal Growth and Subsequent Perinatal Outcomes Using a Multiple Embryo Implantation Model. 2016. 11 (11): e0166222.
41. Tatsumi T, Jwa SC, Kuwahara A, Irahara M, Kubota T, Saito H. No increased risk of major congenital anomalies or adverse pregnancy or neonatal outcomes following letrozole use in assisted reproductive technology. Human Reproduction. 2016. 32(1):125-32.
42. Pereira N, Tsolakian I, Hutchinson AP, Stone LD, Lekovich J, Elias R, Rosenwaks Z. Baseline uterine volume assessment predicts term singleton birth in women undergoing fresh in vitro fertilization. Fertility and Sterility. 2017. 108(3):e262.
43. N. L. Sandi-Monroy, T. Winkle, N. Huzurudin, K. Eibner, N. Reeka and F. Gagsteiger. Does in vitro culture of human preimplantation embryos negatively affect perinatal outcomes?. 2014. 102 (3 SUPPL. 1): e306-e307S. K. Sunkara,​ A. La Marca,​ P. T. Seed and Y. Khalaf. Increased risk of preterm birth and low birthweight with very high number of oocytes following IVF: an analysis of 65 868 singleton live birth outcomes. 2015. 30 (6): 1473-80.
44. R. Burks, F. Stanczyk, M. Wilson, R. Paulson, K. Bendikson and K. Chung. Can adverse obstetric outcomes after frozen embryo transfer be predicted by estradiol levels or endometrial thickness prior to transfer?. 2013. 100 (3 SUPPL. 1): S282-S283.
45. S. Desmyttere, W. Verpoest, M. De Rycke, C. Staessen, A. De Vos, I. Liebaers and M. Bonduelle. Neonatal outcome of 995 children conceived after embryo biopsy compared to children born after intracytoplasmic sperm injection. 2010. 25: i249
46. S. M. Nelson and D. A. Lawlor. Predicting live birth,​ preterm delivery,​ and low birth weight in infants born from in vitro fertilisation: a prospective study of 144,​018 treatment cycles. 2011. 8 (1).

**Did not evaluate spontaneous conception as a control group**.

1. G. M. Chambers,​ A. A. Chughtai,​ C. M. Farquhar and Y. A. Wang. Risk of preterm birth after blastocyst embryo transfer: a large population study using contemporary registry data from Australia and New Zealand. 2015. 104 (4): .997-1003. (Australia and New Zealand).
2. Ernstad E, Khatibi A, Petzold M, Wennerholm UB, Bergh C. Obstetric outcome after IVF/ICSI in frozen embryo transfer (FET). American Journal of Obstetrics and Gynecology. 2019. 220(1)
3. A. Khalil, A. Syngelaki, N. Maiz, Y. Zinevich and K. H. Nicolaides. Maternal age and adverse pregnancy outcomes: A cohort study. 2013. 68 (12): 779-781.
4. O. Basso and D. D. Baird. Infertility and preterm delivery, birthweight, and Caesarean section: a study within the Danish National Birth Cohort. 2003. 18 (11): 2478-84Sokalska A, Mainigi MA, Vresilovic J, Senapati S. Elevated estradiol in frozen-thawed embryo transfers cycles and perinatal risk. Fertility and Sterility. 2017. 108(3):e167.
5. S. K. Sunkara, M. S. Kamat and B. Antonisamy. Perinatal outcomes following preimplantation genetic diagnosis versus IVF or ICSI: Analysis of 99,498 singleton live births. 2016. 31: i108-i109Mannini L, Sorbi F, Noci I, Ghizzoni V, Perelli F, Di Tommaso M, Mattei A, Fambrini M. New adverse obstetrics outcomes associated with endometriosis: a retrospective cohort study. Archives of gynecology and obstetrics. 2017. 295(1):141-51.
6. A. Fata, A. El-Ghandour, A. Serour, E. Ahmed, A. Nassar, M. Serag and G. I. Serour. Complications of assisted reproductive technique among 1400 intra-cytoplasmic sperm injection cycles at Azhart unit. 2009. 107: S177.
7. O. Finnstrom, B. Kallen, A. Lindam, E. Nilsson, K.-G. Nygren and P. O. Olausson. Maternal and child outcome after in vitro fertilization--a review of 25 years of population-based data from Sweden. 2011. 90 (5): 494-500.
8. I. Laskov, R. Birnbaum, S. Maslovitz, M. Kupferminc, J. Lessing and A. Many. Outcome of singleton pregnancy in women >= 45 years old: a retrospective cohort study. 2012. 25 (11): 2190-3.
9. A. P. Londero, S. Bertozzi, S. Visentin, A. Fruscalzo, L. Driul and D. Marchesoni. High placental index and poor pregnancy outcomes: a retrospective study of 18,386 pregnancies. 2013. 29 (7): 666-9.
10. A. Murray. Biological risk versus socio-economic advantage: low birth-weight, multiple births and income variations among Irish infants born following fertility treatments. 2014. 183 (4): 667-70.
11. S. Narayan, K. S. Rana, M. Sharma, R. K. Sharma, P. Talwar, K. Kapur and B. K. Goyal. Profile of Live-born Infants of In-vitro Fertilisation. 2010. 66 (1): 18-21.
12. P. Kuivasaari-Pirinen, K. Raatikainen, M. Hippelainen and S. Heinonen. Adverse Outcomes of IVF/ICSI Pregnancies Vary Depending on Aetiology of Infertility. 2012. 2012: 451915.
13. S. K. Kathpalia, K. Kapoor and A. Sharma. Complications in pregnancies after in vitro fertilization and embryo transfer. 2016. 72 (3): 211-4.
14. O. Finnstrom, B. Kallen, A. Lindam, E. Nilsson, K.-G. Nygren and P. O. Olausson. Maternal and child outcome after in vitro fertilization--a review of 25 years of population-based data from Sweden. 2011. 90 (5): 494-500.
15. L. A. Kondapalli, R. Collins, W. B. Schoolcraft and M. Katz-Jaffe. Perinatal outcomes among IVF singletons are reassuring after comprehensive chromosomal screening and frozen blastocyst transfer. 2015. 104 (3 SUPPL. 1): e13.
16. Zhang J, Mao X, Wang Y, Chen Q, Lu X, Hong Q, Kuang Y. Neonatal outcomes and congenital malformations in children born after human menopausal gonadotropin and medroxyprogesterone acetate treatment cycles. Archives of gynecology and obstetrics. 2017. 296(6):1207-17.
17. O. Abuzeid, A. Abdelrahman, E. Behl, G. Kazzi, J. Murphy and M. Abuzeid. Preterm birth and IVF-ET in singleton pregnancy after hysteroscopic correction of uterine septum. 2016. 127: 73S.
18. N. Imudia,​ A. O. Awonuga,​ J. O. Doyle,​ D. L. Wright,​ T. L. Toth and A. K. Styer. The impact of peak serum estradiol level during controlled ovarian hyperstimulation on adverse obstetrical outcomes in singleton pregnancy conceived following IVF-ET. 2011. 96 (3 SUPPL. 1): S189.
19. U. B. Wennerholm, A. Sazonova, K. Kallen, A. Thurin and C. Bergh. Obstetric outcome in singletons after in-vitro fertilization with frozen-thawed embryos. 2012. 206 (1 SUPPL. 1): S42.
20. N. Imudia, A. O. Awonuga, A. J. Kaimal, D. L. Wright, A. K. Styer and T. L. Toth. Elective cryopreservation of all embryos with interval cryothaw transfer in patients at risk for OHSS reduces the risk of SGA and preeclampsia compared to similar group of patients with fresh transfer. 2012. 98 (3 SUPPL. 1): S184.
21. Macer ML, Barritt J, Surrey MW, Danzer H, Ghadir S, Chang W, Pisarska MD. Pregnancy outcomes following single versus double euploid embryo transfer. Fertility and Sterility. 2017. 107(3):e28-9.
22. Tomas, P. Humaidan, S. Pelkonen, A. Perheentupa, S. Nuojua-Huttunen, H. Tinkanen and H. Martikainen. The birth weight of term singletons after frozen-thawed embryo transfer (FET) treatments is higher using hormonal substitution than natural cycle. 2016. 31: i381.
23. Boerwinkle CH, Tolcher MC, Hoff HS, Aagaard KM. 768: Fresh versus frozen embryo transfer and obstetric and neonatal outcomes. American Journal of Obstetrics & Gynecology. 2018. 218(1):S459-60.
24. Jing S, Ge L, Fei G, Guangxiu L, Changfu L, Shuoping Z. Influence of natural and artificial cycle on obstetric and neonatal outcomes of cryo-thawed embryo transfer: a retrospective study Human Reproduction 2017. 32:497-498.
25. Pereira N, Tsolakian I, Petrini AC, Stone LD, Lekovich J, Elias R, Rosenwaks Z. Does the hyperestrogenic milieu in programmed frozen embryo cycles impact pregnancy and perinatal outcomes?. Fertility and Sterility. 2017. 108(3): e373-4.
26. S. Breheny, L. h. D, J. Halliday, A. Jaques, D. Rushford, C. Garrett, J. M. Talbot and H. W. G. Baker. Fresh is not always best. An analysis of birth outcomes for women having fresh and frozen embryo transfers. 2010. 50: 23-24.
27. P. Bergh and T. A. Molinaro. The risk of low birth weight in pregnancies conceived after frozen embryo transfer may be modified by the extent of estrogen exposure. 2013. 100 (3 SUPPL. 1): S96.
28. Karagozoglu, S. Kahraman, H. Yelke, G. Karlikaya, Y. Guler and Y. Kumtepe. Neonatal outcome of 197 babies born after 706 frozen-thawed embryo transfer cycles; comparing two different cryopreservation techiques. 2010. 25: i152.
29. Sazonova, C. Bergh, K. Kallen, A. Thurin-Kjellberg and U. B. Wennerholm. Obstetric outcome for IVF children born after single or double embryo transfer. 2010. 25: i241.
30. S. McClelland, K. Melzer, D. H. McCulloh and J. D. Grifo. A comparison of pregnancy outcomes between day 3 and day 5/6 fresh embryo transfers: Does day of embryo transfer (ET) really make a difference?. 2013. 100 (3 SUPPL. 1): S92.
31. U. B. Wennerholm, A. K. Henningsen, L. B. Romundstad, R. Skjaerven, K. G. Nygren, M. Gissler, A. Tiitinen and A. Pinborg. Perinatal outcome in singletons born after replacement of frozen/thawed embryos. 2013. 208 (1 SUPPL.1): S20.
32. X. Li, Y. Ouyang and Y. Yi. Pregnancy outcomes of single versus double embryo transfer in women with a congenital unicornuate uterus. 2016. 106: e207.
33. T. Shavit, G. Oron, T. Tulandi, W. Son, H. Holzer and W. Buckett. Single embryo transfer of frozen-thawed embryos is associated with increased maternal complications. 2015. 104 (3 SUPPL. 1): e196.
34. Sekigawa K, Suyama F, Beck W, Mukoda Y, Sasaki K, Okubo H, Sato M, Hara S, Ikuta S, Tokutomi R, Jinno A. Perinatal outcome in singletons after frozen-thawed blastocyst transfer: hormone replacement treatment cycles versus natural cycles. Human Reproduction. 33: 495-496.
35. Xu S.; Guo Y. A singleton pregnancy recommendation for women with polycystic ovary syndrome undergoing invitro fertili zation/intracytoplasmic sperm injection: A propensity score matching study Journal of Assisted Reproduction and Genetics. 2018. 35 (11): 2105.
36. T. Aydin and B. Yucel. Prediction of preterm deliveries by cervical length measurement with catheter before embryo transfer in ICSI pregnancies. 2014. 29: i276-i277.
37. S. Chan, Y. Greenstein, D. Dasig and L. Farah-Eways. Perinatal outcomes after fresh versus frozen embryo transfers. 2016. 106: e324-e325K. Styer, S. L. Mumford, T. Plowden, A. M. Lee, A. Christy and V. L. Baker. Racial disparities in live birth pregnancy outcomes following fresh elective single embryo transfer: A sart-cors analysis 2004-2013. 2016. 106: e98.
38. S. K. Sunkara, P. Seed and Y. Khalaf. Perinatal outcomes following stimulated versus natural cycle IVF: Analysis of 90,980 singleton live births following stimulated and unstimulated IVF. 2015. 30: i16.
39. Y. Khalaf, P. Seed and S. Sunkara. Does ovarian stimulation affect embryo implantation and perinatal outcomes more in fresh IVF transfer than in frozen embryo transfer cycles?. 2015. 30: i369.
40. Y. A. Wang, A. A. Chughtai, C. Farquhar and G. Chambers. No excess risk of preterm birth for singletons following blastocyst transfers compared to cleavage stage embryo transfers in Australia and New Zealand. 2015. 30: i360.
41. S. Jing, H. He, Y. Q. Tan, K. Tan, D. H. Cheng, K. L. Luo, F. Gong, J. Li, G. Lin and G. X. Lu. Obstetric and neonatal outcomes from trophoblast biopsy combined with frozen embryo transfer versus cleavage biopsy with fresh embryo transfer in PGD/PGS treatment. 2015. 30: i55-i56.
42. Li X, Ouyang Y, Wen Y. The perinatal outcomes of women with congenital unicornuate uterus after in vitro fertilization-embryo transfer. Fertility and Sterility. 2017. 108(3):e16.
43. M. Barsky, D. Wilson, D. Bernson, Y. Zhang and C. K. Sites. Embryo cryopreservation with warming increases preeclampsia compared to fresh embryo transfer. 2016. 106: e320.
44. L. Nervi, P. A. Bergh and T. A. Molinaro. Early subchorionic bleed (SCB) in assisted reproductive technology (ART) pregnancies does not influence birth weight. 2016. 106: e87.
45. L. Sekhon, K. Connolly, N. Herlihy, J. Rodriguez-Purata, J. A. Lee and A. B. Copperman. Perinatal outcomes in live births from fresh versus frozen single embryo transfers. 2016. 106: e203.
46. K. Styer, W. Vitek, M. S. Christianson, V. Baker, A. Armstrong, N. Santoro, B. Luke and A. J. Polotsky. Elective single embryotransfer (eSET) is associated with non-low birth weight term singleton outcomes: An analysis of 263,375 cycles. 2015. 104 (3 SUPPL. 1): e16.
47. G. Weiss,​ L. T. Goldsmith,​ R. Sachdev,​ S. Von Hagen and K. Lederer. Elevated first-trimester serum relaxin concentrations in pregnant women following ovarian stimulation predict prematurity risk and preterm delivery. 1993. 82 (5): 821-8.
48. Wang M, Hao C, Bao H, Huang X, Liu Z, Zhang W, Li F. Effect of elevated estradiol levels on the hCG administration day on IVF pregnancy and birth outcomes in the long GnRH-agonist protocol: analysis of 3393 cycles. Archives of gynecology and obstetrics. 2017. 295(2):407-14.
49. Zhang J, Sun L, Du M, Li Z, Hu J, Feng Y, Zhao B, Wang L. Freeze-only versus fresh embryo transfer in full-term singleton birth: a retrospective cohort study Human Reproduction 2018. 33: 487-487.
50. Vidal M, Vellvé K, González-Comadran M, Robles A, Prat M, Torné M, Carreras R, Checa MA. Perinatal outcomes in children born after fresh or frozen embryo transfer: a Catalan cohort study based on 14,262 newborns. Fertility and sterility. 2017. 107(4):940-7.
51. L. Leniaud, C. Poncelet, R. Porcher, B. Martin-Pont, I. Cedrin-Durnerin, J. N. Hugues, J. P. Wolf and C. Sifer. [Prospective evaluation of elective single-embryo transfer versus double-embryo transfer following in vitro fertilization: a two-year French hospital experience]. 2008. 36 (2):159-65.
52. R. Juneau, J. M. Franasiak, S. J. Morin, T. A. Molinaro and R. T. Scott. Obstetric outcomes following frozen embryo transfer (FET) in patients with an endometrial thickness measuring<7mm. 2016. 106 (#Issue#) e214-e215-.
53. Rallis and K. Tremellen. Controlled ovarian hyper-stimulation during IVF treatment does not increase the risk of preterm delivery compared to the transfer of frozen-thawed embryos in a natural cycle. 2013. 53 (2): 165-9.
54. Thurin, J. Hausken, T. Hillensjo, B. Jablonowska, A. Pinborg, A. Strandell and C. Bergh. Elective single-embryo transfer versus double-embryo transfer in in vitro fertilization. 2004. 351 (23): 2392-402.
55. Boerwinkle C, Tolcher M, Hoff H, Aagaard K. Obstetric and neonatal outcomes after cleavage versus blastocyst stage embryo transfer. American Journal of Obstetrics and Gynecology. 2019. 220(1).
56. Zhou W, Zhuang Y, Pan Y, Xia F. Effects and safety of GnRH-a as a luteal support in women undertaking assisted reproductive technology procedures: follow-up results for pregnancy, delivery, and neonates. Archives of gynecology and obstetrics. 2017. 295(5):1269-75.
57. Xiong X, Dickey RP, Buekens P, Shaffer JG, Pridjian G. Use of Intracytoplasmic sperm injection and birth outcomes in women conceiving through in vitro fertilization. Paediatric and perinatal epidemiology. 2017. 31(2):108-15.
58. Wu YT, Li C, Zhu YM, Zou SH, Wu QF, Wang LP, Wu Y, Yin R, Shi CY, Lin J, Jiang ZR. Outcomes of neonates born following transfers of frozen-thawed cleavage-stage embryos with blastomere loss: a prospective, multicenter, cohort study. BMC medicine. 2018. 16(1):96.
59. Schofield SJ, Doughty VL, van Stiphout N, Franklin RC, Johnson MR, Daubeney PE, Cullinan P. Assisted conception and the risk of CHD: a case–control study. Cardiology in the Young. 2017 Mar;27(3):473-9.
60. Amini P, Moghimbeigi A, Zayeri F, Mahjub H, Maroufizadeh S, Samani RO. Evaluating the impact of risk factors on birth weight and gestational age: a multilevel joint modeling approach. International journal of fertility & sterility. 2018. 12(2):106.
61. Cho K, Dunne C, Albert AY, Havelock JC. Adverse perinatal outcomes associated with crown-rump length discrepancy in in vitro fertilization pregnancies. Fertility and sterility. 2018. 109(1):123-9.
62. Li W, Xue X, Zhao W, Ren A, Zhuo W, Shi J. Blastocyst transfer is not associated with increased unfavorable obstetric and perinatal outcomes compared with cleavage-stage embryo transfer. Gynecological Endocrinology. 2017. 33(11):857-60.
63. Sites CK, Wilson D, Barsky M, Bernson D, Bernstein IM, Boulet S, Zhang Y. Embryo cryopreservation and preeclampsia risk. Fertility and sterility. 2017. 108(5):784-90.
64. Wu Y, Chen W, Zhou L, Gao X, Xi X. Single embryo transfer improve the perinatal outcome in singleton pregnancy. The Journal of Maternal-Fetal & Neonatal Medicine. 2019 Jan 15(just-accepted):1-1.
65. E. Kolibianakis,​ K. Osmanagaoglu,​ L. De Catte,​ M. Camus,​ M. Bonduelle,​ I. Liebaers,​ A. Van Steirteghem and P. Devroey. Prenatal genetic testing by amniocentesis appears to result in a lower risk of fetal loss than chorionic villus sampling in singleton pregnancies achieved by intracytoplasmic sperm injection. 2003. 79 (2): 374-8.
66. G. Oron,​ T. Sokal-Arnon,​ W.-Y. Son,​ E. Demirtas,​ W. Buckett,​ A. Zeadna,​ H. Holzer and T. Tulandi. Extended embryo culture is not associated with increased adverse obstetric or perinatal outcome. 2014. 211 (2): 165.e1-7.
67. D. Mayhew, S. Byerley, M. Herbert, A. Murdoch and L. Hyslop. Neonatal outcomes following cleavage versus blastocyst stage transfer. 2015. 18 (4).
68. R. Maslarska, M. Kalaidjieva, V. Konstantinova, S. Deneva, S. Kontilska, V. Alexandrova, E. Igova and B. Ivanova. Risk factors among low birth weight infants, born with assisted reproductive technology. 2014. 27: 403-404.
69. Al Chami, D. Mavrelos, M. Davies, A. Doshi and E. Yasmin. Impact of an elective single embryo transfer programme on live birth rates following in vitro fertilisation. 2016. 19 (1).
70. K. Takeshima,​ S. C. Jwa,​ H. Saito,​ A. Nakaza,​ A. Kuwahara,​ O. Ishihara,​ M. Irahara,​ F. Hirahara,​ Y. Yoshimura and T. Sakumoto. Impact of single embryo transfer policy on perinatal outcomes in fresh and frozen cycles-analysis of the Japanese Assisted Reproduction Technology registry between 2007 and 2012. 2016. 105 (2): 337-46.e3.
71. Aytoz,​ L. De Catte,​ M. Camus,​ M. Bonduelle,​ E. Van Assche,​ I. Liebaers,​ A. Van Steirteghem and P. Devroey. Obstetric outcome after prenatal diagnosis in pregnancies obtained after intracytoplasmic sperm injection. 1998. 13 (1O): 2958-61.
72. D. Fernando,​ J. L. Halliday,​ S. Breheny and D. L. Healy. Outcomes of singleton births after blastocyst versus nonblastocyst transfer in assisted reproductive technology. 2012. 97 (3): 579-84.
73. D. Zander-Fox and M. Lane. BMI: Does fat really matter? The impact of BMI on embryo development and pregnancy during IVF. 2009. 49: A29.
74. E. C. Nelissen,​ A. P. Van Montfoort,​ E. Coonen,​ J. G. Derhaag,​ J. P. Geraedts,​ L. J. Smits,​ J. A. Land,​ J. L. Evers and J. C. Dumoulin. Further evidence that culture media affect perinatal outcome: findings after transfer of fresh and cryopreserved embryos. 2012. 27 (7): 966-76.
75. E. J. Forman,​ K. H. Hong,​ J. M. Franasiak and R. T. Scott,​ Jr.. Obstetrical and neonatal outcomes from the BEST Trial: single embryo transfer with aneuploidy screening improves outcomes after in vitro fertilization without compromising delivery rates. 2014. 210 (2): 157.e1-6.
76. E. K. Sauber-Schatz,​ W. Sappenfield,​ V. Grigorescu,​ A. Kulkarni,​ Y. Zhang,​ H. M. Salihu,​ L. P. Rubin,​ R. S. Kirby,​ D. J. Jamieson and M. Macaluso. Obesity,​ assisted reproductive technology,​ and early preterm birth--Florida,​ 2004-2006. 2012. 176 (10): 886-96
77. F. Zegers-Hochschild,​ J. E. Schwarze,​ J. A. Crosby,​ C. Musri and M. do Carmo Borges de Souza. Assisted reproductive technologies in Latin America: the Latin American Registry,​ 2012. 2015. 30 (1): 43-51.
78. G. Rose,​ V. Beral,​ J. A. Davis,​ R. G. Edwards,​ P. S. Harper,​ J. D. O. Loudon,​ B. Mason,​ D. C. Taylor,​ J. Modle,​ M. Hally and J. Gunning. Births in Great Britain resulting from assisted conception,​ 1978-87. 1990. 300 (6734): 1229-1233.
79. J. G. Lemmen,​ A. Pinborg,​ S. Rasmussen and S. Ziebe. Birthweight distribution in ART singletons resulting from embryo culture in two different culture media compared with the national population. 2014. 29 (10): 2326-32.
80. J. Gunby,​ F. Bissonnette,​ C. Librach,​ L. Cowan,​ I. V. F. D. G. o. t. C. Fertility and S. Andrology. Assisted reproductive technologies in Canada: 2005 results from the Canadian Assisted Reproductive Technologies Register. 2009. 91 (5): 1721-30.
81. J. Qin,​ X. Sheng,​ D. Wu,​ S. Gao,​ Y. You,​ T. Yang and H. Wang. Adverse Obstetric Outcomes Associated With In Vitro Fertilization in Singleton Pregnancies. Reproductive sciences (Thousand Oaks, Calif.) / 2017;24(4):595-608
82. J. Zhu,​ Y. Lian,​ M. Li,​ L. Chen,​ P. Liu and J. Qiao. Does IVF cleavage stage embryo quality affect pregnancy complications and neonatal outcomes in singleton gestations after double embryo transfers?. 2014. 31 (12): 1635-41.
83. T. Tatsumi,​ S. C. Jwa,​ A. Kuwahara,​ M. Irahara,​ T. Kubota and H. Saito. No increased risk of major congenital anomalies or adverse pregnancy or neonatal outcomes following letrozole use in assisted reproductive technology. 2017. 32 (1): 125-132.
84. V. Soderstrom-Anttila,​ U.-B. Wennerholm,​ A. Loft,​ A. Pinborg,​ K. Aittomaki,​ L. B. Romundstad and C. Bergh. Surrogacy: outcomes for surrogate mothers,​ children and the resulting families-a systematic review. 2016. 22 (2): 260-76.
85. K. Ozgur,​ H. Bulut,​ M. Berkkanoglu and K. Coetzee. Reproductive outcomes of IVF patients with unicornuate uteri. 2017. 34 (3): 312-318.
86. L. A. Schieve,​ C. Ferre,​ H. B. Peterson,​ M. Macaluso,​ M. A. Reynolds and V. C. Wright. Perinatal outcome among singleton infants conceived through assisted reproductive technology in the United States. 2004. 103 (6): 1144-53.
87. P. Doyle,​ V. Beral and N. Maconochie. Preterm delivery,​ low birthweight and small-for-gestational-age in liveborn singleton babies resulting from in-vitro fertilization. 1992. 7 (3): 425-8.
88. R. Lin,​ G. Feng,​ J. Shu,​ B. Zhang,​ H. Zhou,​ X. Gan,​ C. Wang and H. Chen. Blastocoele re-expansion time in vitrified-warmed cycles is a strong predictor of clinical pregnancy outcome. 2017. 43 (4): 689-695.
89. S. L. Boulet,​ S. Crawford,​ Y. Zhang,​ S. Sunderam,​ B. Cohen,​ D. Bernson,​ P. McKane,​ M. A. Bailey,​ D. J. Jamieson,​ D. M. Kissin and A. R. T. C. States Monitoring. Embryo transfer practices and perinatal outcomes by insurance mandate status. 2015. 104 (2): 403-9.e1.
90. S. Pelkonen,​ M. Gissler,​ S. Koivurova,​ S. Lehtinen,​ H. Martikainen,​ A. L. Hartikainen and A. Tiitinen. Physical health of singleton children born after frozen embryo transfer using slow freezing: a 3-year follow-up study. 2015. 30 (10): 2411-8.
91. S. Sibtain,​ D. Janga,​ A. Shah and A. Gudi. Outcomes after IVF-frozen embryo versus fresh transfer. 2012. 119: 140.
92. X.-L. Hu,​ C. Feng,​ X.-H. Lin,​ Z.-X. Zhong,​ Y.-M. Zhu,​ P.-P. Lv,​ M. Lv,​ Y. Meng,​ D. Zhang,​ X.-E. Lu,​ F. Jin,​ J.-Z. Sheng,​ J. Xu and H.-F. Huang. High maternal serum estradiol environment in the first trimester is associated with the increased risk of small-for-gestational-age birth. 2014. 99 (6): 2217-24.
93. Z. J. Chen,​ Y. Shi,​ Y. Sun,​ B. Zhang,​ X. Liang,​ Y. Cao,​ J. Yang,​ J. Liu,​ D. Wei,​ N. Weng,​ L. Tian,​ C. Hao,​ D. Yang,​ F. Zhou,​ J. Shi,​ Y. Xu,​ J. Li,​ J. Yan,​ Y. Qin,​ H. Zhao,​ H. Zhang and R. S. Legro. Fresh versus frozen embryos for infertility in the polycystic ovary syndrome. 2016. 375 (6): 523-533.
94. Moaddab A, Chervenak FA, Mccullough LB, Sangi-Haghpeykar H, Shamshirsaz AA, Schutt A, Arian SE, Fox KA, Dildy GA, Shamshirsaz AA. Effect of advanced maternal age on maternal and neonatal outcomes in assisted reproductive technology pregnancies. European Journal of Obstetrics & Gynecology and Reproductive Biology. 2017. 216:178-83.
95. Chen X, Liu P, Sheng Y, Li W, Tang R, Ding L, Qin Y, Chen ZJ. The impact of unicornuate uterus on perinatal outcomes after IVF/ICSI cycles: a matched retrospective cohort study. The Journal of Maternal-Fetal & Neonatal Medicine. 2018. 2:1-6.
96. Prior M, Richardson A, Asif S, Polanski L, Parris‐Larkin M, Chandler J, Fogg L, Jassal P, Thornton JG, Raine‐Fenning NJ. Outcome of assisted reproduction in women with congenital uterine anomalies: a prospective observational study. Ultrasound in Obstetrics & Gynecology. 2018. 51(1):110-7.
97. Keyhan S, Truong T, Li YJ, Jackson-Bey T, Eaton JL. Preterm delivery and low birth weight among neonates conceived with intracytoplasmic sperm injection compared with conventional in vitro fertilization. Obstetrics & Gynecology. 2018. 131(2):262-8.
98. Kamath MS, Antonisamy B, Selliah HY, La Marca A, Sunkara SK. Perinatal outcomes following IVF with use of donor versus partner sperm. Reproductive biomedicine online. 2018. 36(6):705-10.
99. De Vos A, Santos-ribeiro S, Van Landuyt L, Van de Velde H, Tournaye H, Verheyen G. Birthweight of singletons born after cleavage-stage or blastocyst transfer in fresh and warming cycles. Human Reproduction. 2018. 33(2):196-201.
100. Bourdon M, Santulli P, Kefelian F, Vienet-Legue L, Maignien C, Pocate-Cheriet K, De Mouzon J, Marcellin L, Chapron C. Prolonged estrogen (E2) treatment prior to frozen-blastocyst transfer decreases the live birth rate. Human Reproduction. 2018. 33(5):905-13.
101. Du T, Wang Y, Fan Y, Zhang S, Yan Z, Yu W, Xi Q, Chen Q, Mol BW, Lyu Q, Kuang Y. Fertility and neonatal outcomes of embryos achieving blastulation on Day 7: are they of clinical value?. Human Reproduction. 2018. 33(6):1038-51.
102. Oron G, Hiersch L, Rona S, Prag-Rosenberg R, Sapir O, Tuttnauer-Hamburger M, Shufaro Y, Fisch B, Ben-Haroush A. Endometrial thickness of less than 7.5 mm is associated with obstetric complications in fresh IVF cycles: a retrospective cohort study. Reproductive biomedicine online. 2018. 37(3):341-8.
103. Ben-Haroush A, Sirota I, Salman L, Son WY, Tulandi T, Holzer H, Oron G. The influence of body mass index on pregnancy outcome following single-embryo transfer. Journal of assisted reproduction and genetics. 2018. 35(7):1295-300.
104. Palmsten K, Homer MV, Zhang Y, Crawford S, Kirby RS, Copeland G, Chambers CD, Kissin DM, Su HI, Collaborative SM. In vitro fertilization, interpregnancy interval, and risk of adverse perinatal outcomes. Fertility and sterility. 2018. ;109(5):840-8.
105. Zhang J, Du M, Li Z, Wang L, Hu J, Zhao B, Feng Y, Chen X, Sun L. Fresh versus frozen embryo transfer for full-term singleton birth: a retrospective cohort study. Journal of ovarian research. 2018. 11(1):59.
106. Holden EC, Kashani BN, Morelli SS, Alderson D, Jindal SK, Ohman-Strickland PA, McGovern PG. Improved outcomes after blastocyst-stage frozen-thawed embryo transfers compared with cleavage stage: a Society for Assisted Reproductive Technologies Clinical Outcomes Reporting System study. Fertility and sterility. 2018. 110(1):89-94.
107. Amrane S, Brown MB, Lobo RA, Luke B. Factors associated with short interpregnancy interval among women treated with in vitro fertilization. Journal of assisted reproduction and genetics. 2018. 35(9):1595-602.
108. de Wilde MA, Lamain-de Ruiter M, Veltman-Verhulst SM, Kwee A, Laven JS, Lambalk CB, Eijkemans MJ, Franx A, Fauser BC, Koster MP. Increased rates of complications in singleton pregnancies of women previously diagnosed with polycystic ovary syndrome predominantly in the hyperandrogenic phenotype. Fertility and sterility. 2017. 108(2):333-40.
109. Sunkara SK, Antonisamy B, Selliah HY, Kamath MS. Perinatal outcomes after gestational surrogacy versus autologous IVF: analysis of national data. Reproductive biomedicine online. 2017. 35(6):708-14.
110. Sabban H, Zakhari A, Patenaude V, Tulandi T, Abenhaim HA. Obstetrical and perinatal morbidity and mortality among in-vitro fertilization pregnancies: a population-based study. Archives of gynecology and obstetrics. 2017. 296(1):107-13.
111. Litzky JF, Boulet SL, Esfandiari N, Zhang Y, Kissin DM, Theiler RN, Marsit CJ. Effect of frozen/thawed embryo transfer on birthweight, macrosomia, and low birthweight rates in US singleton infants. American journal of obstetrics and gynecology. 2018. 218(4):433-e1.
112. Choux C, Barberet J, Ginod P, Cottenet J, Bruno C, Benzénine E, Sagot P, Fauque P. Severe ovarian hyperstimulation syndrome modifies early maternal serum beta-human chorionic gonadotropin kinetics, but obstetrical and neonatal outcomes are not impacted. Fertility and sterility. 2017. 108(4):650-8.
113. Quinn MM, Rosen MP, Huddleston HG, Cedars MI, Fujimoto VY. Interpregnancy Interval and Singleton Live Birth Outcomes From In Vitro Fertilization. Obstetrics & Gynecology. 2018. 132(1):115-21.
114. Y.-h. Lu,​ H.-j. Gao,​ B.-j. Li,​ Y.-m. Zheng,​ Y.-h. Ye,​ Y.-l. Qian,​ C.-m. Xu,​ H.-f. Huang and F. Jin. Different sperm sources and parameters can influence intracytoplasmic sperm injection outcomes before embryo implantation. 2012. 13 (1): 1-10.
115. Z. Li,​ Y. A. Wang,​ W. Ledger,​ D. H. Edgar and E. A. Sullivan. Clinical outcomes following cryopreservation of blastocysts by vitrification or slow freezing: a population-based cohort study. 2014. 29 (12): 2794-801.
116. Y. Guan,​ H. Fan,​ A. K. Styer,​ Z. Xiao,​ Z. Li,​ J. Zhang,​ L. Sun,​ X. Wang and Z. Zhang. A modified natural cycle results in higher live birth rate in vitrified-thawed embryo transfer for women with regular menstruation. 2016. 62 (5): 335-342.
117. Y. Wu,​ X. Kang,​ H. Zheng,​ H. Liu,​ Q. Huang and J. Liu. Effect of Paternal Age on Reproductive Outcomes of Intracytoplasmic Sperm Injection. 2016. 11 (2): e0149867.
118. X. Xiong,​ G. Pridjian and R. P. Dickey. Racial and ethnic disparities in preterm births in infants conceived by in vitro fertilization in the United States. 2013. 209 (2): 128.e1-6.
119. V. Vernaeve,​ M. Bonduelle,​ H. Tournaye,​ M. Camus,​ A. Van Steirteghem and P. Devroey. Pregnancy outcome and neonatal data of children born after ICSI using testicular sperm in obstructive and non-obstructive azoospermia. 2003. 18 (10): 2093-7.
120. W. Li,​ X. Xue,​ W. Zhao,​ A. Ren,​ W. Zhuo and J. Shi. Blastocyst transfer is not associated with increased unfavorable obstetric and perinatal outcomes compared with cleavage-stage embryo transfer. 2017. 1-4.
121. V. Y. Fujimoto,​ B. Luke,​ M. B. Brown,​ T. Jain,​ A. Armstrong,​ D. A. Grainger,​ M. D. Hornstein and G. Society for Assisted Reproductive Technology Writing. Racial and ethnic disparities in assisted reproductive technology outcomes in the United States. 2010. 93 (2): 382-90.
122. V. Savasi,​ M. Oneta,​ B. Parrilla and I. Cetin. Should HCV discordant couples with a seropositive male partner be treated with assisted reproduction techniques (ART)?. 2013. 167 (2): 181-4.
123. V. De Frene,​ S. Vansteelandt,​ G. T'Sjoen,​ J. Gerris,​ S. Somers,​ L. Vercruysse and P. De Sutter. A retrospective STUDY of the pregnancy,​ delivery and neonatal outcome in overweight versus normal weight women with polycystic ovary syndrome. 2014. 29 (10): 2333-2338.
124. U. B. Wennerholm,​ C. Bergh,​ L. Hamberger,​ G. Westlander,​ M. Wikland and M. Wood. Obstetric outcome of pregnancies following ICSI,​ classified according to sperm origin and quality. 2000. 15 (5): 1189-1194.
125. T. Fujii,​ O. Wada-Hiraike,​ T. Nagamatsu,​ M. Harada,​ T. Hirata,​ K. Koga,​ T. Fujii and Y. Osuga. Assisted reproductive technology pregnancy complications are significantly associated with endometriosis severity before conception: a retrospective cohort study. 2016. 14 (1): 73.
126. S. Sunderam,​ D. M. Kissin,​ S. B. Crawford,​ S. G. Folger,​ D. J. Jamieson,​ L. Warner and W. D. Barfield. Assisted Reproductive Technology Surveillance - United States,​ 2014. 2017. 66 (6): 1-24.
127. S. Porat,​ S. Savchev,​ Y. Bdolah,​ A. Hurwitz and R. Haimov-Kochman. Early serum beta-human chorionic gonadotropin in pregnancies after in vitro fertilization: contribution of treatment variables and prediction of long-term pregnancy outcome. 2007. 88 (1): 82-9.
128. S. K. Kalra,​ S. J. Ratcliffe,​ K. T. Barnhart and C. Coutifaris. Extended embryo culture and an increased risk of preterm delivery. 2012. 120 (1): 69-75.
129. S. K. Sunkara,​ A. LaMarca,​ N. P. Polyzos,​ P. T. Seed and Y. Khalaf. Live birth and perinatal outcomes following stimulated and unstimulated IVF: analysis of over two decades of a nationwide data. 2016. 31 (10): 2261-7.
130. S. Dar,​ C. L. Librach,​ J. Gunby,​ F. Bissonnette,​ L. Cowan,​ I. V. F. D. G. o. C. Fertility and S. Andrology. Increased risk of preterm birth in singleton pregnancies after blastocyst versus Day 3 embryo transfer: Canadian ART Register (CARTR) analysis. 2013. 28 (4): 924-8.
131. S. Fernando,​ S. Breheny,​ A. M. Jaques,​ J. L. Halliday,​ G. Baker and D. Healy. Preterm birth,​ ovarian endometriomata,​ and assisted reproduction technologies. 2009. 91 (2): 325-30.
132. R. Isaksson,​ A. Tiitinen and B. Cacciatore. Uterine artery impedance to blood flow on the day of embryo transfer does not predict obstetric outcome. 2000. 15 (6): 527-30.
133. O. J. Carpinello,​ P. R. Casson,​ C.-L. Kuo,​ R. S. Raj,​ E. S. Sills and C. A. Jones. Cost Implications for Subsequent Perinatal Outcomes After IVF Stratified by Number of Embryos Transferred: A Five Year Analysis of Vermont Data. 2016. 14 (3): 387-95.
134. O. Ishihara,​ R. Araki,​ A. Kuwahara,​ A. Itakura,​ H. Saito and G. D. Adamson. Impact of frozen-thawed single-blastocyst transfer on maternal and neonatal outcome: an analysis of 277,​042 single-embryo transfer cycles from 2008 to 2010 in Japan. 2014. 101 (1): 128-33.
135. N. Pereira,​ A. C. Petrini,​ J. P. Lekovich,​ G. L. Schattman and Z. Rosenwaks. Comparison of perinatal outcomes following fresh and frozen-thawed blastocyst transfer. 2016. 135 (1): 96-100.
136. M. Wikland,​ T. Hardarson,​ T. Hillensjo,​ C. Westin,​ G. Westlander,​ M. Wood and U. B. Wennerholm. Obstetric outcomes after transfer of vitrified blastocysts. 2010. 25 (7): 1699-707.
137. M. M. Aboulghar,​ M. A. Aboulghar,​ Y. M. Amin,​ H. G. Al-Inany,​ R. T. Mansour and G. I. Serour. The use of vaginal natural progesterone for prevention of preterm birth in IVF/ICSI pregnancies. 2012. 25 (2): 133-8.
138. M. Locci,​ G. Nazzaro,​ A. Merenda,​ M. L. Pisaturo,​ P. Laviscio,​ R. Poppiti,​ M. Miranda,​ A. Stile and G. De Placido. Atosiban vs ritodrine used prophylactically with cerclage in ICSI pregnancies to prevent pre-term birth in women identified as being at high risk on the basis of transvaginal ultrasound scan. 2006. 26 (5): 396-401.
139. M. Bonduelle,​ B. Mannaerts,​ A. Leader,​ C. Bergh,​ D. Passier and P. Devroey. Prospective follow-up of 838 fetuses conceived after ovarian stimulation with corifollitropin alfa: comparative and overall neonatal outcome. 2012. 27 (7): 2177-85.
140. M. Jacques,​ T. Freour,​ P. Barriere and S. Ploteau. Adverse pregnancy and neo-natal outcomes after assisted reproductive treatment in patients with pelvic endometriosis: a case-control study. 2016. 32 (6): 626-34.
141. L. Chen,​ Z. Xu,​ N. Zhang,​ B. Wang,​ H. Chen,​ S. Wang and H. Sun. Neonatal outcome of early rescue ICSI and ICSI with ejaculated sperm. 2014. 31 (7): 823-8.
142. L. Benaglia,​ G. Candotti,​ E. Papaleo,​ L. Pagliardini,​ M. Leonardi,​ M. Reschini,​ L. Quaranta,​ M. Munaretto,​ P. Vigano,​ M. Candiani,​ P. Vercellini and E. Somigliana. Pregnancy outcome in women with endometriosis achieving pregnancy with IVF. 2016. 31 (12): 2730-2736.
143. K. Vicdan,​ C. Akarsu,​ E. Sozen,​ B. Buluc,​ A. Vicdan,​ Y. Yilmaz and K. Biberoglu. Outcome of intracytoplasmic sperm injection using fresh and cryopreserved-thawed testicular spermatozoa in 83 azoospermic men with Klinefelter syndrome. 2016. 42 (11): 1558-1566.
144. K. Ozgur,​ M. Berkkanoglu,​ H. Bulut,​ P. Humaidan and K. Coetzee. Perinatal outcomes after fresh versus vitrified-warmed blastocyst transfer: retrospective analysis. 2015. 104 (4): 899-907.e3.
145. K. Devine,​ M. T. Connell,​ K. S. Richter,​ C. I. Ramirez,​ E. D. Levens,​ A. H. DeCherney,​ R. J. Stillman and E. A. Widra. Single vitrified blastocyst transfer maximizes liveborn children per embryo while minimizing preterm birth. 2015. 103 (6): 1454-60.e1.
146. K. Kapiteijn,​ C. S. de Bruijn,​ E. de Boer,​ A. J. M. de Craen,​ C. W. Burger,​ F. E. van Leeuwen and F. M. Helmerhorst. Does subfertility explain the risk of poor perinatal outcome after IVF and ovarian hyperstimulation?. 2006. 21 (12): 3228-34.
147. K. C. Calhoun,​ M. A. Fritz and A. Z. Steiner. Examining the relationship between ovarian reserve,​ as measured by basal FSH levels,​ and the risk of poor obstetric outcome in singleton IVF gestations. 2011. 26 (12): 3424-30.
148. J. Zeitlin,​ M.-J. Saurel-Cubizolles,​ J. De Mouzon,​ L. Rivera,​ P.-Y. Ancel,​ B. Blondel and M. Kaminski. Fetal sex and preterm birth: are males at greater risk?. 2002. 17 (10): 2762-8.
149. S. Fernando,​ S. Breheny,​ A. M. Jaques,​ J. L. Halliday,​ G. Baker and D. Healy. Preterm birth,​ ovarian endometriomata,​ and assisted reproduction technologies. 2009. 91 (2): 325-30.
150. J.-B. Qin,​ X.-Q. Sheng,​ D. Wu,​ S.-Y. Gao,​ Y.-P. You,​ T.-B. Yang and H. Wang. Worldwide prevalence of adverse pregnancy outcomes among singleton pregnancies after in vitro fertilization/intracytoplasmic sperm injection: a systematic review and meta-analysis. 2017. 295 (2): 285-301.
151. L. Benaglia,​ G. Candotti,​ E. Papaleo,​ L. Pagliardini,​ M. Leonardi,​ M. Reschini,​ L. Quaranta,​ M. Munaretto,​ P. Vigano,​ M. Candiani,​ P. Vercellini and E. Somigliana. Pregnancy outcome in women with endometriosis achieving pregnancy with IVF. 2016. 31 (12): 2730-2736.
152. S. K. Sunkara,​ A. LaMarca,​ N. P. Polyzos,​ P. T. Seed and Y. Khalaf. Live birth and perinatal outcomes following stimulated and unstimulated IVF: analysis of over two decades of a nationwide data. 2016. 31 (10): 2261-7.
153. F. Belva,​ M. Bonduelle,​ M. Roelants,​ G. Verheyen and L. Van Landuyt. Neonatal health including congenital malformation risk of 1072 children born after vitrified embryo transfer. 2016. 31 (7): 1610-20.
154. M. Vidal,​ K. Vellve,​ M. Gonzalez-Comadran,​ A. Robles,​ M. Prat,​ M. Torne,​ R. Carreras and M. A. Checa. Perinatal outcomes in children born after fresh or frozen embryo transfer: a Catalan cohort study based on 14,​262 newborns. 2017. 107 (4): 940-947.
155. Bay, H. J. Ingerslev, J. G. Lemmen, B. Degn, I. A. Rasmussen and U. S. Kesmodel. Preimplantation genetic diagnosis: a national multicenter obstetric and neonatal follow-up study. 2016. 106 (6):1363-1369.
156. B. Morse,​ K. T. Barnhart,​ S. Senapati,​ M. D. Sammel,​ E. C. Prochaska,​ A. Dokras,​ C. Chatzicharalampous and C. Coutifaris. Association of the very early rise of human chorionic gonadotropin with adverse outcomes in singleton pregnancies after in vitro fertilization. 2016. 105 (5): 1208-1214.e3.
157. Y. B. Jeve,​ N. Potdar,​ A. Opoku and M. Khare. Three-arm age-matched retrospective cohort study of obstetric outcomes of donor oocyte pregnancies. 2016. 133 (2): 156-8.
158. L. Zhou,​ X. Gao,​ Y. Wu and Z. Zhang. Analysis of pregnancy outcomes for survivors of the vanishing twin syndrome after in vitro fertilization and embryo transfer. 2016. 203: 35-9.
159. Q.-Z. Xie,​ W.-M. Xu,​ Q.-R. Qi,​ Z.-L. Luo and L. Dong. Patients with cervical Ureaplasma Urealyticum and Chlamydia Trachomatis infection undergoing IVF/ICSI-ET: The need for new paradigm. 2016. 36 (5): 716-722.
160. F. Zegers-Hochschild,​ J. E. Schwarze,​ J. A. Crosby,​ C. Musri,​ M. T. Urbina and R. Latin American Network of Assisted. Assisted reproductive techniques in Latin America: the Latin American Registry,​ 2013. 2016. 32 (6): 614-25.
161. Luke,​ M. B. Brown,​ E. Wantman,​ J. E. Stern,​ J. P. Toner and C. C. Coddington,​ 3rd. Increased risk of large-for-gestational age birthweight in singleton siblings conceived with in vitro fertilization in frozen versus fresh cycles. 2017. 34 (2): 191-200.
162. W. Mak,​ L. A. Kondapalli,​ G. Celia,​ J. Gordon,​ M. DiMattina and M. Payson. Natural cycle IVF reduces the risk of low birthweight infants compared with conventional stimulated IVF. 2016. 31 (4): 789-94.
163. L. Sterling,​ J. Liu,​ N. Okun,​ A. Sakhuja,​ S. Sierra and E. Greenblatt. Pregnancy outcomes in women with polycystic ovary syndrome undergoing in vitro fertilization. 2016. 105 (3): 791-7.e2.
164. S. Korosec,​ H. B. Frangez,​ L. Steblovnik,​ I. Verdenik and E. V. Bokal. Independent factors influencing large-for-gestation birth weight in singletons born after in vitro fertilization. 2016. 33 (1): 9-17.
165. K. Takeshima,​ S. C. Jwa,​ H. Saito,​ A. Nakaza,​ A. Kuwahara,​ O. Ishihara,​ M. Irahara,​ F. Hirahara,​ Y. Yoshimura and T. Sakumoto. Impact of single embryo transfer policy on perinatal outcomes in fresh and frozen cycles-analysis of the Japanese Assisted Reproduction Technology registry between 2007 and 2012. 2016. 105 (2): 337-46.e3.
166. J.-E. Schwarze,​ J. A. Crosby and F. Zegers-Hochschild. Effect of embryo freezing on perinatal outcome after assisted reproduction techniques: lessons from the Latin American Registry of Assisted Reproduction. 2015. 31 (1): 39-43.
167. Huang,​ K. Qian,​ Z. Li,​ J. Yue,​ W. Yang,​ G. Zhu and H. Zhang. Neonatal outcomes after early rescue intracytoplasmic sperm injection: an analysis of a 5-year period. 2015. 103 (6): 1432-7.e1.
168. J. Fechner,​ K. R. Brown,​ N. Onwubalili,​ S. K. Jindal,​ G. Weiss,​ L. T. Goldsmith and P. G. McGovern. Effect of single embryo transfer on the risk of preterm birth associated with in vitro fertilization. 2015. 32 (2): 221-4.
169. N. Imudia,​ A. O. Awonuga,​ A. J. Kaimal,​ D. L. Wright,​ A. K. Styer and T. L. Toth. Elective cryopreservation of all embryos with subsequent cryothaw embryo transfer in patients at risk for ovarian hyperstimulation syndrome reduces the risk of adverse obstetric outcomes: a preliminary study. 2013. 99 (1): 168-73.
170. N. Imudia,​ A. O. Awonuga,​ J. O. Doyle,​ A. J. Kaimal,​ D. L. Wright,​ T. L. Toth and A. K. Styer. Peak serum estradiol level during controlled ovarian hyperstimulation is associated with increased risk of small for gestational age and preeclampsia in singleton pregnancies after in vitro fertilization. 2012. 97 (6):1374-9.
171. L. Benaglia,​ A. Bermejo,​ E. Somigliana,​ C. Scarduelli,​ G. Ragni,​ L. Fedele and J. A. Garcia-Velasco. Pregnancy outcome in women with endometriomas achieving pregnancy through IVF. 2012. 27 (6): 1663-7.
172. Sazonova,​ K. Kallen,​ A. Thurin-Kjellberg,​ U.-B. Wennerholm and C. Bergh. Factors affecting obstetric outcome of singletons born after IVF. 2011. 26 (10): 2878-86.
173. Courbiere,​ V. Oborski,​ D. Braunstein,​ A. Desparoir,​ A. Noizet and M. Gamerre. Obstetric outcome of women with in vitro fertilization pregnancies hospitalized for ovarian hyperstimulation syndrome: a case-control study. 2011. 95 (5): 1629-32.
174. N.-H. Morken. Preterm delivery in IVF versus ICSI singleton pregnancies: a national population-based cohort. 2011. 154 (1): 62-6.
175. M.-J. Pelinck,​ M. H. Keizer,​ A. Hoek,​ A. H. M. Simons,​ K. Schelling,​ K. Middelburg and M. J. Heineman. Perinatal outcome in singletons after modified natural cycle IVF and standard IVF with ovarian stimulation. 2010. 148 (1): 56-61.
176. Tarlatzi TB, Imbert R, Mercadal BA, Demeestere I, Venetis CA, Englert Y, Delbaere A. Does oocyte donation compared with autologous oocyte IVF pregnancies have a higher risk of preeclampsia?. Reproductive biomedicine online. 2017. 34(1):11-8.

## Written in a language other than English and French

1. M. Storgaard, S. Malchau, A. Loft, E. Larsen and A. Pinborg. [Oocyte donation is associated with an increased risk of complications in the pregnant woman and the fetus]. 2017. 179 (11).
2. W. Jiang, F. Jin and L. Zhou. [Influence of the DNA integrity of optimized sperm on the embryonic development and clinical outcomes of in vitro fertilization and embryo transfer]. 2016. 22 (5): 425-31.
3. P. Basta, J. Schwarz, L. Laskowicz and A. Kotlarz. [Fertility-sparing surgical management of ovarian cancer]. 2015. 86 (10): 759-64.
4. L. Mo, C. Jin, Y. Wu, P. Wang, J. Lin and J. Zhao. [Incidence and management of monozygotic twin conceived by assisted reproductive techniques]. 2015. 50 (8): 586-90.
5. J. Reichelt, I. Kyvernitakis, B. Misselwitz, P. Hadji, S. Schmidt and M. Kalder. A population based evaluation of the mode of delivery in association with infertility treatment from 1990-2012. 2015. 219 (1): 37-44.
6. Anonymous. [Preimplantation genetic diagnosis and neonatal outcome]. 2014. 153 (12): 701-754.
7. L. Sun, Z. Chen, J. Liu and J. Fu. [Obstetric and neonatal outcomes of vanishing twin syndrome]. 2014. 34 (10): 1537-40.
8. G. Shaoqin, Z. Zhenghui, Z. Xueqian and H. Yuan. [Epigenetic modifications in human spermatozoon and its potential role in embryonic development]. 2014. 36 (5): 439-46.
9. V. V. Grabar. [Interconnection between assisted reproductive technologies, pregnancy complications and risk of birth defects]. 2014. (227): 7-14.
10. M. Lima, M. Sousa, C. Oliveira, J. Silva, J. Teixeira Da Silva, M. Cunha, P. Viana and A. Barros. [Ovarian hyperstimulation syndrome: experience of a reproductive medicine center 2005-2011]. 2013. 26 (1): 24-32.
11. M. Serdynska-Szuster, P. Jedrzejczak, K. Ozegowska, M. Korman and L. Pawelczyk. [Perinatal outcome among women undergoing in vitro fertilization procedures complicated by ovarian hyperstimulation syndrome]. 2012. 83 (2): 104-10.
12. J. Zamlynski, J. Rzempoluch, S. Horak, P. Bodzek, P. Binkiewicz and B. Zasada. [Perinatal outcome in multiple pregnancy after natural reproduction and ART techniques]. 2000. 71 (11): 1496-502.
13. T. Korzon, W. Gromadzki, M. Adamcio-Deptulska and J. Anders. [Pregnancy and labor after treatment of infertility in women with developmental anomalies of the uterus]. 1982. 53 (5-6): 321-6.
14. W. Gromadzki. [Determination of the degree of obstetrical risk in different states of marital subfertility]. 1974. 45 (7): 815-43.
15. B. Slancheva, N. yarakova, T. Pramatarova, S. Hitrova, P. Radulova, L. Vakrilova, A. Nikolov and T. Garnisov. ["In vitro" babies--dreams, that turned real, but a lot of questions and unknown items]. 2012. 51 (2): 3-11.
16. A.-K. A. Henningsen, A. Loft, S. S. Malchau and A. Pinborg. [The prognosis for children born after assisted reproduction]. 2012. 174 (41): 2462-6.
17. J.-H. Shu, B. Zhang, G.-X. Feng, X.-Y. Gan, H. Zhou, L. Zhou and Y. Liu. [Influence of sperm morphology on the outcomes and neonatal status in IVF-ET]. 2010. 16 (10): 897-900.
18. C. Bergh and U.-B. Wennerholm. [Low risk of illness among children born after fertilization in vitro]. 2010. 107 (42): 2554-9.\
19. H. Mumdzhiev and L. Pekova. [The assisted reproduction and the newborn child]. 2009. 48 (4): 34-40.
20. M. K. Bohlmann, B. Fritzsching, D. W. Luedders, A. Hornemann, W. Gopel, J. Poschl, K. Diedrich and G. Griesinger. [Impact of assisted reproduction on obstetrics and neonatology]. 2009. 213 (6): 221-7.
21. J.-Z. Zhao, X. Chen, P.-Y. Wang, W. Zhou, J.-J. Lin, W. Zhang, X.-F. Huang, W.-Q. Lin, H.-Y. Yang and Y. Chen. [Outcome of pregnancy in women with polycystic ovary syndrome treated by in vitro maturation of immature oocytes]. 2009. 44 (6): 409-12.
22. P. Fehr, K.-G. Nygren and C. De Geyter. [Effect of different embryo transfer strategies on the outcome of assisted reproduction]. 2009. 66 (12): 825-9
23. .F. Wang, Y.-P. Sun, H.-J. Kong, Y.-C. Su, Y.-H. Guo, J.-Y. Liang and P.-F. Li. [Analysis of 1540 neonates with birth defects related to different assisted reproductive technique]. 2009. 44 (2): 108-11.
24. V. R. Graner and S. M. O. de Barros. [Maternal complications and neonatal events associated to multiple pregnancies resulting from assisted reproduction techniques]. 2009. 43 (1): 103-9.
25. G. Sebastiani, A. Pertierra Cortada, E. Vidal Sorde, J. Figueras Aloy and J. Balasch Cortina. [Factors associated with assisted reproduction technologies and neonatal outcomes]. 2009. 70 (4): 323-32.
26. S.-l. Chen, S.-z. Li, L. Sun, H.-d. Song, J.-x. He, L.-h. Kong, L. Zhu, H. Li and F.-q. Xing. [Neonatal outcomes of pregnancy resulting from in vitro fertilization and embryo transfer: analysis of 1274 cases]. 2007. 27 (4): 439-41.
27. L. Sassi, E. Gorbe, E. Hruby, M. Perlaki, J. Jeager and Z. Papp. [The outcome of triplet pregnancies at the First Department of Obstetrics and Gynecology, Semmelweis University, in the last 15 years]. 2006. 147 (45): 169-73.
28. A. Teixeira, L. Calejo, G. Vasconcellos, G. Rocha, M. J. Centeno and H. Guimaraes. [Newborns from assisted reproductive technology at the Hospital de Sao Joao]. 2005. 18 (6): 409-15.
29. O. Hellmann and Y. Bentov. [Congenital malformations in children born after IVF]. 2005. 144 (12): 852-910.
30. A. Egic, Z. Mikovic, D. Filmonovic and A. Cirovic. [Birth weight discordance and perinatal mortality among triplets]. 2005. 133 (5-6): 254-7.
31. I. Oborna, J. Brezinova, M. Svobodova, M. Krskova, J. Dostal and S. Machac. [Multiple pregnancy--a significant complication of in vitro fertilization protocols]. 2003. 68 (4): 232-7.
32. B. Backe. [Assisted insemination--a risk factor in perinatal health care]. 2002. 122 (3): 317-8.
33. E. Garzena, A. Ventriglia, A. Patanella, A. Simonitti, A. Mammano, S. Garbarini, S. Costa, L. Becchino and C. Fabris. [Various features of neonatal outcome in assisted fertilization pregnancies]. 2000. 71 Suppl 1: 479-81.
34. B. Galanti, C. T. Kaihura, L. Ricci, L. Bedocchi, T. Rossi, G. Benassi and L. Benassi. [Perinatal morbidity and mortality in children born to mothers with gestational hypertension]. 2000. 71 Suppl 1: 361-5.
35. V. von During, J. M. Maltau, F. Forsdahl, T. Abyholm, R. Kolvik, G. Ertzeid, A. Steier, V. Baste and L. M. Irgens. [Pregnancy, births and infants after in-vitro-fertilization in Norway, 1988-1991]. 1995. 115 (17): 2054-60.
36. S. Ishii, K. Tanaka, T. Okai, T. Tanaka, T. Takahashi, K. Aoki, Y. Torii, F. Saji, K. Takahashi and K. Sato. [Perinatal outcome of pregnancies following therapy of infertility]. 1994. 46 (12): 1305-10.
37. K. Fiedler, W. Wurfel, G. Krusmann, M. Rothenaicher, P. Hirsch and W. Krusmann, Sr.. [Course of pregnancy and labor following in vitro fertilization. A retrospective study of 246 deliveries]. 1990. 194 (1): 8-12.
38. R. Wiedemann, M. Korell, T. Strowitzki and H. Hepp. [Course of pregnancy following in vitro fertilization and embryo transfer]. 1990. 194 (1): 1-7.
39. C. Fabris, D. Licata and E. Garzena. [The newborn infant of an assisted-fertilization pregnancy. A clinico-statistical survey]. 1990. 12 (5): 471-6.
40. V. Markova and R. Ianeva. [The course of pregnancy and labor following artificial insemination]. 1989. 28 (5): 29-34.
41. K. Fiedler, G. Krusmann, M. Rothenaicher, P. Hirsch and W. Wurfel. [295 children born following IVF/ET. An analysis of the course of pregnancy and labor]. 1989. 29 (Suppl 2): 306-8.
42. R. Lindemann and P. H. Finne. [Pediatric aspects of in vitro fertilization]. 1988. 108 (5): 405-392.
43. M. Sas. [Obstetric results and problems after induction of ovulation]. 1977. 95 (25): 2122-4.
44. M. Langer. Postmenopausal pregnancy after egg donation. 2014. 47 (4): 244-247.
45. A. Jakimiuk and A. Fritz-Rdzanek. Obstetric complications after assisted reproductive technology. 2012. 8 (1): 33-36.
46. X. X. Wang, C. X. Luan, W. Zhang and S. M. Hu. [Pregnancy outcomes of in vitro fertilization and embryo transfer in infertile women with polycystic ovarian syndrome]. 2012. 47 (10): 730-733.
47. M. Kasum. Outcome of pregnancy after in vitro fertilization. 2009. 53 (2): 77-81.
48. M. Kasum. Multiple pregnancies after in vitro fertilization. 2009. 18 (1): 23-29.
49. S. Kissler, I. Wiegratz and M. Kaufmann. Complications and risks of infertility treatment. 2007. 31 (4): 647-658.
50. W. Urdl, R. Felberbaum and W. Kupker. The problem of multiple pregnancies after assisted reproductive techniques. 2007. 5 (4): 212-218.
51. O. Finnstrom, K. G. Nygren and P. O. Olausson. . 2006. 103 (32-33): 2301-2305.
52. U. Ulug, A. Mesut, E. A. Jozwiak and M. Bahceci. Obstetric outcome of triplet pregnancies beyond viability following ICSI-ET. 2006. 20 (2): 109-113.
53. H. Kentenich and P. Kolm. Parent-child relationship after assisted reproductive medicine. 2006. 13 (2-4): 154-161.
54. B. Peter, S. Gveric-Ahmetasevic and D. Svaljug. Perinatal and neuromotor outcome of newborns after IVF-ET procedure. 2006. 15 (1): 65-72.
55. N. S. Severinski, O. Mamula and I. Vlastelic. Adverse perinatal outcome after assisted reproductive technology. 2006. 15 (1): 73-76.
56. S. Kilic, A. Beytur, B. Altunoluk, L. Beytur, F. Oguz and R. Atmaca. Results of 78 intrauterine insemination (IUI) cycles with husband sperm in infertility and factors affecting IUI success: A retrospective study. 2005. 31 (4): 516-523.
57. M. Ludwig. Risks involved in pregnancy and birth after assisted reproduction. 2005. 29 (1): 27-35.
58. I. Oborna, J. Brezinova, M. Svobodova, M. Krskova, J. Dostal and S. Machac. Multiple pregnancies - Important complication of IVF programme. 2003. 68 (4): 232-237.
59. M. Ludwig, M. Kohl, A. Kruger, M. Loning, A. Schroder, A. Katalinic and K. Diedrich. Complications in High Order Multiple Pregnancies. 2004. 64 (2): 168-177.
60. P. De Sutter and M. Dhont. Assisted reproductive technologies: Possible risks and complications for mother and child. 2003. 59 (21): 1283-1291.
61. A. Romeu, A. Monzo, P. J. Fernandez-Colom and S. Oehninger. Multiple pregnancy: How important is the problem?. 2002. 19 (3): 181-194.
62. T. Honda, K. Umeoka, T. Nakahori, A. Takahashi, Y. Isozaki, T. Takahashi and Y. Umaoka. Retrospective analysis of multiple pregnancies in in vitro fertilization. 2002. 47 (1): 55-60.
63. C. Fuhlrott and G. Jorch. Assisted reproduction techniques from the view of a pediatrician. 2001. 17 (2): 100-102.
64. N. Sakai, Y. Kishimoto, T. Saito, M. Ito, K. Nakahara, H. Saito and M. Hiroi. Perinatal risks after in vitro fertilization and embryo transfer. 1996. 41 (3): 35-42.
65. J. Weller. Course of pregnancy and childbirth after 104 successful artificial donogenic inseminations (ADI) with native and cold-storage sperm. 1980. 40 (3): 269-275.
66. de la Fuente L, Romero B, Monzó A, Casas AB, Castell AB, Llaneza P, Hernández C, Sáez MJ, Vacas R, Vázquez A, Alvarez C. Reproducción en pacientes de más de 40 años: estudio multicéntrico de los resultados obstétricos de 2.578 partos. Medicina Reproductiva y Embriología Clínica. 2018 Sep 1;5(3):145-53.
67. Wei DM, Zhang ZZ, Wang Z, Li P, Wang JF, Liu YJ, Zhang JT, Shi YH. Effect of hyperandrogenism on obstetric complications of singleton pregnancy from in vitro fertilization in women with polycystic ovary syndrome. Zhonghua fu chan ke za zhi. 2018 Jan;53(1):18-22.
68. Piera SG, Canals LC, Grau EG, Pueyo JC, Cantarell SM, Pérez C, Hernández RR, Estévez YC. Resultados obstétricos y perinatales en gestaciones tras técnicas de reproducción asistida. Progresos de Obstetricia y Ginecología 2017;60(3): 208-213.
69. Zhao L, Chen L, Wang T, Zheng Z, Zhang S, Ye Z, Qin J. Impact of in vitro fertilization-embryo transfer on adverse pregnancy outcomes: A prospective cohort study. Zhong nan da xue xue bao. Yi xue ban= Journal of Central South University. Medical sciences. 2018 Dec;43(12):1328-36.

## Study design: Abstract

1. Boyer P, Anzola AB, Pauly V, Sambuc R, Riviere O, Venditelli F, Boyer MJ. No differences found of Small for Gestational Age (SGA) between Fresh Embryo Transfer (ET) babies and Spontaneously Conceived (SC) ones. Human Reproduction 2018. 33:486-486.
2. Ernstad E, Khatibi A, Petzold M, Wennerholm UB, Bergh C. Obstetric outcome after IVF/ICSI in frozen embryo transfer (FET). American Journal of Obstetrics and Gynecology. 2019. 220(1).
3. Dimitriadis, M. Batsis, J. C. Petrozza, D. L. Wright and I. Souter. Are infertile women conceiving with in-vitro fertilization (IVF) at higher risk for adverse pregnancy outcomes than those conceiving with ovulation induction/intrauterine insemination (OI/IUI)?. 2013. 100 (3 SUPPL. 1): S491
4. O. Awonuga, A. N. Imudia, D. R. Armant, F. D. Yelian, M. P. Diamond and E. E. Puscheck. Is the level of estrogen on the day of hCG and pattern of estrogen rise during superovulation associated with adverse pregnancy outcomes in IVF/ICSI cycles?. 2009. 92 (3 SUPPL. 1): S168.
5. D. Nasioudis, L. El-Kabab, G. Sisti, T. Kanninen, A. M. Bongiovanni, S. S. Witkin and S. S. Spandorfer. The S100A8 concentration in sera prior to initiation of an in vitro fertilization (IVF) cycle predicts subsequent outcome. 2016. 23 (1 SUPPL. 1): 167A.
6. S. M. Nelson and D. A. Lawlor. Predicting live birth, preterm and low birth weight infant after in-vitro fertilization: A prospective study of 144,018 treatment cycles. 2010. 94 (4 SUPPL. 1): S82.
7. H. S. Wolff, R. P. Gada, P. H. Leonard and C. C. Coddington. Clinical implications of pregestational cervical length in patients undergoing IVF. 2011. 96 (3 SUPPL. 1): S13.
8. O. Mor, M. Stavsky, S. A. Mastrolia, B. Hamou, R. Beer-Weisel, T. Rafaeli, L. Besser, M. Mazor and O. Erez. Preeclampsia, SGA and cerebral palsy: Lesson from a population-based study. 2015. 212 (1 SUPPL. 1): S365.
9. T. Eldar-Geva, N. Srebnik, E. J. Margalioth, O. Schonberger, I. Varshaver, A. Perez, E. Rubinstein, P. Renbaum, N. Dekel, E. Levy-Lahad and G. Altarescu. Embryo biopsy for PGD has no significant impact on neonatal outcome. 2013. 26: S22.
10. D. A. Grainger, L. M. Frazier and C. A. Rowland. Preconception care and treatment with assisted reproductive technologies. 2006. 10 (SUPPL. 7): 161-164.
11. Anonymous. Abstracts of the 19th National Congress on Infertility and Reproduction. 2013. 11.
12. Silverman and Wojtowycz. The effects of in vitro fertilization on subsequent pregnancy outcome. 1998. 5 (4): 169.
13. T. Jackson-Bey, S. Keyhan, Y. Li, T. Truong and J. L. Eaton. Peak serum estradiol and neonatal birth weight following in vitro fertilization. 2016. 106: e179-e180.
14. N. Sharpe and M. Choudhary. Reproductive outcome following pre-implantation genetic diagnosis (PGD)-an analysis of UK national database over two decades. 2015. 104 (3 SUPPL. 1): e281.
15. H. C. Van Os, P. M. Rijnders, K. E. Tucker and C. A. M. Jansen. The obstetric outcome of singletons after compulsory single embryo transfer (CSET). 2009. 24: i149-i150.
16. M. Kupka, C. Calhaz-Jorge, J. A. Castilla Alcala, C. De Geyter, J. De Mouzon, T. D'Hooghe, K. Erb, A. P. Ferraretti and V. Goossens. Assisted reproductive technology (ART) in Europe 2012. Preliminary results generated from
17. O. Wada-Hiraike, T. Nagamatsu, M. Harada, T. Hirata, K. Koga, T. Fujii and Y. Osuga. Art pregnancy
18. complications are significantly associated with endometriosis severity before conception: A retrospective cohort study. 2017. 24 (1 Supplement 1): 232A-233A.
19. Moaddab, H. Sangi-Haghpeykar, S. Arian, S. Ramin, Z. Heidari-Bateni, H. Erfani, K. Fox, S. Clark, M. Belfort, G. Dildy, L. McCullough, F. Chervenak and A. Shamshirsaz. Effect of very advanced maternal age on early neonatal outcomes after assisted reproductive technology. 2017. 24 (1 Supplement 1): 262A-263A.
20. L. Sun, M. Xiaojuan, H. Jijun, Q. Ruofan, G. Yichun and W. Xingling. The effect of Fresh-ET and FET on maternal and neonatal outcomes. 2016. 31: i176.
21. L. Benaglia, G. Candotti, E. Papaleo, L. Pagliardini, M. Leonardi, M. Reschini, L. Quaranta, M. Munaretto, P. Vigano, M. Candiani, P. Vercellini and E. Somigliana. Pregnancy outcome in women with endometriosis achieving pregnancy with in vitro fertilization. 2016. 31: i250.
22. Harlev, I. Har-Vardi, E. Lunenfeld, A. Zeadna, I. Bord, G. Bar, M. Friger, R. Sergienko and E. Levitas. Is fertility treatment an additional perinatal risk factor in women over 40 years old?. 2016. 31: i5-i6.
23. Q. Chen, H. Ye, X. Ding, X. Shen, G. Huang, H. Deng, H. Wen, L. Pei and P. Zeng. A retrospective analysis of outcomes of 585 selectively reduced multiple pregnancies in IVF/ICSI-ET cycles. 2016. 31: i288.
24. H. He, S. Jing, Y. Q. Tan, K. L. Luo, D. H. Cheng, F. Gong, G. X. Lu and G. Lin. Blastocyst biopsy in PGD/PGS cycles does not increase the neonatal risk when compared to intracytoplasmic sperm injection cycles. 2016. 31: i374-i375.
25. Ozkok, N. Yilmaz, A. Karatas, A. Tokmak and S. Cavdar. Is there any predictive capability of the first beta-HCG level in IN vitro fertilization cycles. 2016. 106: e181.
26. J. F. Kawwass, A. Kulkarni, H. Hipp, S. Crawford, D. M. Kissin and D. J. Jamieson. Assisted reproductive technology cycle and obstetric outcomes among underweight and overweight women. 2016. 106: e21.
27. M. Barsky, P. St Marie, T. Rahil, G. R. Markenson and C. K. Sites. Is placental function affected by cryopreservation and warming in human in vitro fertilization cycles?. 2016. 23 (1 SUPPL. 1): 260A.
28. U. B. Wennerholm, E. G. Ernstad, A. Khatibi, K. Kallen and C. Bergh. Obstetric outcome after IVF/ICSI with blastocyst transfer (BC). 2016. 214 (1 SUPPL. 1): S69.
29. N. Zaninovic, Q. Zhan, R. Clarke, Z. Ye, J. Malmsten and Z. Rosenwaks. Perinatal outcome using time-lapse system and reduced oxygen culture in IVF patients. 2015. 104 (3 SUPPL. 1): e227-e228.
30. G. D. Royster, J. Csokmay, B. Yauger, A. DeCherney, E. F. Wolff and M. J. Hill. High estradiol and ICSI increase the risk of placental complications in pregnancy. 2015. 104 (3 SUPPL. 1): e222.
31. S. A. Greenberg, S. O'Gradney, L. Davis, E. A. Zbella and M. Sanchez. Perinatal risk in microtubal reanastomosis versus in vitro fertilization in patients with tubal factor infertility. 2015. 104 (3 SUPPL. 1): e177-e178.
32. T. Kanninen, I. Ramer, G. Sisti, S. S. Witkin and S. D. Spandorfer. Concentrations of insulin-like growth factor (IGF)-1 and 2 at the beginning of a matched donor cycle before stimulation predict outcome in recipients. 2015. 104 (3 SUPPL. 1): e149.
33. K. Perkins, S. Boulet, D. M. Kissin and D. J. Jamieson. Trends and outcomes of assisted reproductive technology (ART) cycles using gestational carriers in the United States, 1998-2012. 2015. 104 (3 SUPPL. 1): e100.
34. S. Korosec, H. Ban Frangez, L. Steblovnik, I. Verdenik and E. Vrtacnik Bokal. A multivariate analysis of large-for-gestation risk factors in IVF and naturally conceived singletons. 2015. 30: i16.
35. M. Kupka, C. Calhaz-Jorge, J. A. Castilla Alcala, C. De Geyter, J. De Mouzon, T. D'Hooghe, K. Erb, A. P. Ferraretti and V. Goossens. Assisted reproductive technology in Europe, 2011: Results generated from European registers by ESHRE. Preliminary results. 2014. 29: i54-i55.
36. W. Mak, L. A. Kondapalli, M. DiMattina, J. D. Gordon, G. Celia and M. Payson. Fewer preterm births in unstimulated versus stimulated in vitro fertilization (IVF). 2014. 102 (3 SUPPL. 1): e18-e19.
37. F. Verit, S. Keskin, A. K. Sargin, S. Karahuseyinoglu, O. Yucel and S. Yalcinkaya. Pregnancy outcomes in poor responders after ICSI cycles. 2013. 28: i334.
38. L. El Khattabi, E. Hafhouf, D. Royere, J. L. Pouly, J. De Mouzon and R. Levy. ICSI with non-ejaculated sperm is associated to higher perinatal risks than ICSI with ejaculated sperm or IVF. 2013. 28: i96-i97.
39. S. Senapati, S. Boudhar, M. D. Sammel, C. B. Morse and K. T. Barnhart. The association of endometriosis and preterm delivery: An evaluation from the society for reproductive technologies (SART) database. 2013. 100 (3 SUPPL. 1): S513-S514.
40. P. Bergh and T. A. Molinaro. Area under the curve (AUC) can be used to estimate the effect of estrogen exposure on pregnancy outcome during fresh IVF cycles. 2013. 100 (3 SUPPL. 1): S495.
41. M. Y. M. Leung, S. H. Chang, L. M. Pollack, J. S. Rhee and E. S. Jungheim. Neonatal outcomes for obese women undergoing in vitro fertilization (IVF). 2013. 100 (3 SUPPL. 1): S340.
42. V. Grigorescu, Y. Zhang, D. Kissin, E. Sauber-Schatz, M. Sunderam and D. Jamieson. Maternal characteristics and pregnancy out comes after assisted reproductive technologies (ART) by infertility diagnosis: Ovulatory dysfunction (OD) versus tubal obstruction (TO). 2012. 98 (3 SUPPL. 1): S200-S201.
43. M. Zanirato, C. N. Kao, P. Caballero Campo, M. I. Cedars, M. Rosen and P. Rinaudo. Advanced paternal age does not affect birth weightand gestational outcome in singleton pregnancies conceived by art. 2012. 98 (3 SUPPL. 1): S102.
44. V. Shavell, Z. Al-Safi, R. Roberts and M. Diamond. First trimester cervical length is associated with preterm delivery in pregnancies conceived with in vitro fertilization. 2011. 18 (6 SUPPL. 1) S157.
45. S. K. Jindal, M. Chuang, D. S. Berger, A. Polotsky, N. Santoro and L. Pal. Increase in ART-conceived miscarriage and preterm birth rates following Hurricane Katrina: Analysis of 104,724 cycles reported to SART. 2011. 96 (3 SUPPL. 1): S283-S284.
46. L. Rubal, N. Opper, B. Kashani, R. J. Paulson and K. Chung. Tubal transfer prior to day 3 is associated with improved perinatal outcomes after IVF. 2011. 96 (3 SUPPL. 1): S274.
47. S. N. Lin, A. Melnick, E. Milbank, M. Biewald and A. Grunebaum. Comparison of neonatal and maternal outcomes in multiparous women 40 years and older with spontaneous versus IVF singleton pregnancies. 2011. 96 (3 SUPPL. 1): S184-S185.
48. Morse, K. T. Barnhart, M. D. Sammel, E. C. Prochaska, A. Dokras and C. Coutifaris. Early rise in human chorionic gonadotropin (hCG) as a marker of placentation: A slow rise may predict low birth weight in assisted reproduction. 2011. 96 (3 SUPPL. 1): S81.
49. O. Doyle, A. N. Imudia, C. Veiga, D. L. Wright, A. K. Styer and T. L. Toth. Obstetrical outcomes of singleton and multiple gestatation IVF pregnancies conceived following transfer of cryopreserved and fresh embryos. 2011. 96 (3 SUPPL. 1): S28-S29.
50. O. Vincent-Boulay, N. G. Mahutte, V. Bissonauth and S. Ouhilal. First trimester cervical length measurement predicts preterm delivery in IVF patients. 2011. 96 (3 SUPPL. 1): S13.
51. Z. A. Al-Safi, V. I. Shavell, R. P. Roberts, M. Singh, E. E. Puscheck and M. P. Diamond. First trimester cervical length and preterm delivery in pregnancies conceived through in vitro fertilization. 2011. 96 (3 SUPPL. 1): S13.
52. R. Nagai, N. Fukunaga, H. Kitasaka, T. Yoshimura, F. Itoi, F. Tamura, K. Kitamura, N. Hasegawa, M. Kato, K. Nakayama, H. Honma, H. Oguri, M. Sano, Y. Hashiba and Y. Asada. Pregnancy & perinatal outcome after in vitro fertilization in 1341 cycles. 2011. 26: i273.
53. Laskov, R. Birenbaum, S. Amzalag, S. Maslovitz, M. Kupferminc, J. Lessing and A. Many. Multiple pregnancy in women >= 45 years old, risks and hazards. 2011. 18 (3 SUPPL. 1): 378A.
54. P. G. McGovern, A. Seungdamrong, D. H. McCulloh, G. Weiss and L. T. Goldsmith. Luteal mass predicts preterm birth. 2011. 18 (3 SUPPL. 1): 309A.
55. Lamazou, M. Frayssinet, M. Grynberg, V. Kerbrat, J. Stephan, N. Frydman, R. Fanchin and R. Frydman. Comparison of obstetrical and neonatal outcomes of pregnancies obtained after preimplantation diagnosis versus intracytoplasmic injection. 2010. 20: S41.
56. M. Bonduelle, S. Desmyttere, W. Verpoest, M. De Rycke, C. Staessen, A. Devos and I. Liebaers. PGD and children follow-up. 2010. 20: S13.
57. T. T. Saroyan, I. E. Korneeva, T. A. Nazarenko and S. G. Perminova. Characteristics of clinical course of IVF pregnancies after severe ovarian hyperstimulation syndrome (OHSS). 2010. 7 (4): 281.
58. T. T. Saroyan, I. E. Korneeva, T. A. Nazarenko and G. T. Sukhikh. Systemic inflammatory condition as a cause of ART pregnancy complications. 2010. 20: S77-S78.
59. E. Forman and R. T. Scott. Pregnancies conceived in women with thin endometria (<= 5MM maximal thickness) are at increased risk for poorobstetrical outcome. 2010. 94 (4 SUPPL. 1): S217-S218.
60. E. C. M. Nelissen, A. P. A. Van Montfoort, E. Coonen, J. G. Derhaag, J. L. H. Evers and J. C. M. Dumoulin. In vitro culture affects the birthweight of human singletons. 2010. 25: i238.
61. Hiraoka, M. Miyazaki, E. Fukunaga, T. Horiuchi, T. Kusuda, S. Okano, M. Kinutani and K. Kinutani. Perinatal outcomes following transfer of human blastocysts vitrifed at day 5, 6 and 7. 2010. 25: i60.
62. Healy. Should fresh et be abandoned in the interests of healthy mothers and babies?. 2009. 49: A11.
63. Z. Kollmann, Q. V. Neri, Z. Rosenwaks and G. D. Palermo. Is conception method or maternal factor affecting perinatal outcome?. 2009. 92 (3 SUPPL. 1): S149.
64. Luke, M. B. Brown, J. E. Stern, S. A. Missmer, V. Y. Fujimoto and R. Leach. Effect of maternal body mass index (BMI) on assisted reproductive technology (ART) pregnancy rates and obstetric outcomes. 2009. 92 (3 SUPPL. 1): S79.
65. Luke, M. B. Brown, J. E. Stern, S. A. Missmer, V. Y. Fujimoto and R. Leach. Maternal obesity adversely affects assisted reproductive technology (ART) pregnancy rates and obstetric outcomes. 2009. 92 (3 SUPPL. 1): S1.
66. McLennan AS, Victoria XY, Ananth CV, Gyamfi-Bannerman C, Miller RS. 277: Does abnormal placental cord insertion at anatomy ultrasound predict small-for-gestational-age infants?. American Journal of Obstetrics & Gynecology. 2017. 216(1): S170.
67. Aviram A, Gabbay-Benziv R, Hiersch L, Ashwal E, Hadar E, Shmueli A, Wiznitzer A, Yogev Y. Similar, yet not the same: pregnancy outcome of lga newborns stratified by the presence or absence of gdm. American Journal of Obstetrics and Gynecology. 2017. 216(1): S339-340.
68. Harlev A, Wainstock T, Walfisch A, Segal I, Landau D, Sheiner E. Long term endocrine disorders in children following assisted reproductive technology (ART) pregnancies compared with spontaneous pregnancies. American Journal of Obstetrics and Gynecology. 2017. 216(1): S349.
69. Croughan MS, Camarano L, Bernstein D, Chamberlain N, Schembri M. Childhood outcomes following infertility and infertility treatment. Fertility and Sterility. Andrology 2017. (5): S28.
70. Akselrod D, Tirosh NB, Stavsky M, Refaeli-Yehudai T, Dorit P, Imtirat M, Greenbaum S, Besser L, Erez O. Risk Factors for Preterm and Term Pre-Eclamptic Pregnancies Complicated by an SGA Neonate-A Population Based Study. Reproductive Sciences 2017. 24: 155A-155A.
71. Shamshirsaz A, Moaddab A, Sangi-Haghpeykar H, Arian S, Ramin S, Heidari-Bateni Z, Erfani H, Fox K, Clark S, Belfort M, Dildy G. Effect of Very Advanced Maternal Age on Early Neonatal Outcomes After Assisted Reproductive Technology. InREPRODUCTIVE SCIENCES 2017. 24:262A-263A.
72. Hollenbach SJ, Miller LA, Olson-Chen C, Li D, Dye T, Thornberg L. Impact of Assisted Reproductive Technologies on Pregnancy Outcomes Among Patients of Extremely Advanced Maternal Age. InReproductive Sciences 2017. 24: 231A-232A.
73. Fujii T, Wada-Hiraike O, Nagamatsu T, Harada M, Hirata T, Koga K, Fujii T, Osuga Y. ART Pregnancy Complications Are Significantly Associated with Endometriosis Severity Before Conception: A Retrospective Cohort Study. InREPRODUCTIVE SCIENCES 2017. 24: 232A-233A.
74. Fritz R, Jindal SK, Yu B, Vega MG, Buyuk E. Does donor sperm affect birth weight (BW), preterm birth (PB), And miscarriage rates in fresh autologous in vitro fertilization (IVF) cycles? analysis of 46,061 cycles reported to SART. Fertility and Sterility. 2017 Mar 1;107(3): e30.
75. Chowdhury G. Polycystic ovarian syndrome (PCOS) and pregnancy outcome, Journal of Obstetrics and Gynaecology Research. 2017. 43(1): 136.
76. Cao M, Liu Z, Yang D. The effect of positive TPO-Ab on pregnancy outcomes of euthyroid IVF women. Human Reproduction. 2017. 32: 464-464.
77. Haahr T, Humaidan P, Elbaek HO, Alsbjerg B, Laursen RJ, Rygaard K, Johannesen TB, Andersen PS, Ng KL, Jensen JS. Vaginal microbiota and IVF outcomes: development of a simple diagnostic tool to predict patients at risk of a poor reproductive outcome. Human Reproduction. 2017. 32(1).
78. Jacobs P, Ombelet W, Janssen M, Van der Auwera I, Vandenborne K, Dhont N, Mestdagh G, Campo R. Pregnant after using the simplified Walking Egg IVF culture system: Perinatal outcome of the first 60 babies. Human Reproduction. 2017. 32:92-93.
79. Pontesilli M. Effect of parental and treatment aspects of IVF/ICSI on perinatal outcomes: a nationwide study of IVF/ICSI singletons born in The Netherlands between 2000 and 2011. Human Reproduction 2017. 32:18-19.
80. Schirmer DA, Kulkarni A, Kawwass JF, Boulet S, Kissin DM. Ovarian hyperstimulation syndrome after assisted reproductive technology: trends, predictors, and pregnancy outcomes. Fertility and Sterility. 2017. 108(3): e23.
81. Del Porto F, Ferrero S, Proietta M, Cifani N, Catoni M, Ubaldi FM, Rienzi L, Ferri L, Di Rosa R. Autoimmunity and pregnancy: evidence from an observational study. Annals of the Rheumatic Diseases. 2017. 76 Supplement 2 879
82. Martinez M, Eichlin H, Molinaro T, Bergh P. Pregnancies achieved after fresh and frozen IVF cycles with a thinner endometrium are at higher risk for preterm delivery and low birth weight. Fertility and Sterility. 2018. 109(3): e37.
83. Gorgui J.; Sheehy O.; Trasler J.; Fraser W.; Berard A. Medically assisted reproduction and the risk of preterm birth Gorgui J.; Birth Defects Research. 2018. 110 (9): 756
84. Chang J, Zhang Y, Boulet SL, Crawford SB, Kissin DM. Assisted Reproductive Technology and Stillbirth Risk: Selected States, 2006–2011 [15op]. Obstetrics & Gynecology. 2018. 131:6S.
85. Abdala A, Vargas L, Passanante EG, Provenzano S, Singla J. ICSI outcomes in couples with male factor infertility do not differ from a control group presenting tubal factor diagnoses. Reproductive BioMedicine Online. 2018. 37: e6.
86. Sheehy O, Trasler J, Fraser W, Berard A. Medically assisted reproduction and the risk of preterm birth. Pharmacoepidemiology and Drug Safety 2018. 27: 237-237.
87. Rudenko E, Demura T, Kogan E, Trifonova N. Structural basis of complications in pregnancy using the donor egg in surrogate maternity. Virchows Archiv 2018. 473: S40-S40.
88. Lo J. A Comparison of Perinatal Outcomes Between Older and Younger Primiparous Women at a Tertiary Hospital. Australian & New Zealand JournaL of ObstetricS & Gynaecology 2018. 58: 64-64.
89. Martin VG, de Bantel AF, Bonnet E, Cedrin-Durnerin I, Merzouk M, Roset J, Avril C, Wainer R, Landais P. In vitro fertilization (IVF) outcomes after dramatic weight loss linked to bariatric surgery: a case-control study. Human Reproduction. 2018. 33:312-312.
90. Oberg S, VanderWeele T, Almqvist C, Hernandez-Diaz S. Pregnancy complications following assisted reproductive techniques-disentangling the role of multiple gestation. Human Reproduction. 2018. 33: 129-130.
91. Chen Y, Nisenblat V, Ma C. Comparison of the treatment outcomes in women with unicornuate uterus undergoing ART versus controls with normal uterine anatomy: a nested case-control retrospective analysis. Human Reproduction 2018. 33: 322-323.
92. Lanes A, Sprague A, Leader A, Potter B, Walker M. The effect of the underlying type of infertility on placental-mediated adverse outcomes for patients that used IVF: a population-level study Human Reproduction 2018. 33: 488-489.
93. Li X, Ouyang Y, Wen Y. Whether the surgical indications for septate uterus are reasonable in infertile patients before in vitro fertilization Human Reproduction. 2018. 33: 24-24.
94. Johnson KM, Hacker MR, Thornton K, Young BC, Modest AM. 713: The association between in vitro fertilization and ischemic placental disease by gestational age. American Journal of Obstetrics & Gynecology. 2019. 220(1): S470.
95. Davenport MJ, Sorby K, Osianlis T, MacLachlan VB, Vollenhoven BJ, Talmor AJ. Gonadotropin-releasing hormone agonist (GnRH-a) triggering may improve live birth rates and reduce ovarian hyperstimulation syndrome (OHSS) in'freeze-all'cycles: time to re-think conventional triggering?. Fertility and Sterility. 2017. 108(3):e238.
96. Stern JE, Liu C, Cabral HJ, Richards EG, Coddington C, Diop H, Missmer SA. Outcomes of cesarean section and vaginal deliveries in art-treated, subfertile and fertile women. Fertility and Sterility. 2017. 108(3):e73.
97. Connell MT, Csokmay JM, Yamasaki MU, Dolitsky S, Healy MW, DeCherney A, Hill MJ. Progesterone on the day of trigger and obstetric outcomes. Fertility and Sterility. 2017. 108(3):e348-9.
98. Litzky JF, Boulet S, Esfandiari N, Zhang Y, Kissin DM, Theiler RN, Marsit C. Birthweight in infants following blastocyst transfer compared to cleavage stage transfer. Fertility and Sterility. 2017. 108(3):e37.
99. Quinn M, Huddleston H, Rosen M, Cedars M, Fujimoto VY. Short interpregnancy interval (IPI) is associated with preterm delivery in singleton live births from a national cohort undergoing assisted reproductive technology (ART). Fertility and Sterility. 2017. 108(3):e40-1.
100. T. Ebner, O. Shebl, K. Tritscher, P. Oppelt, H. C. Duba, M. Maurer, G. Schappacher-Tilp, E. Petek and R. B. Mayer. Qualitative and quantitative grading of human blastocysts and its association with live-birth rate and neonatal outcome. 2015. 30: i44-i45.
101. L. Sterling, J. Liu, A. Sakhuja, S. Sierra, N. Okun and E. Greenblatt. Pregnancy outcomes in women with polycystic ovarian syndrome (PCOS) undergoing in vitro fertilization (IVF). 2015. 30: i371-i372.
102. L. Li, C. Givens and P. Chenette. Supernumerary frozen blastocyst transfers result in unacceptable rates of multiple gestation and preterm delivery. 2016. 105 (2 SUPPL. 1) e30-.
103. Enache, B. Marinescu and T. Enache. Parameters predicting pregnancy and fetal outcome in IVF/ICSI patients: Is it possible?. 2012. 119: S340.
104. Amodeo Hernandez, E. De La Hoz Freitas, B. Rodriguez Rodriguez, E. Mantrana Bermejo, M. R. Ostos Serna and R. Garrido Teruel. Preterm birth risk on multiple gestation: Value of the transvaginal ultrasound cervical length measurement and the fetal fibronectin test. 2010. 23: 307
105. Study design: Systematic review or/and meta-analysis
106. J. Hasson,​ D. Limoni,​ M. Malcov,​ T. Frumkin,​ H. Amir,​ T. Shavit,​ B. Bay,​ A. Many and B. Almog. Obstetric and neonatal outcomes of pregnancies conceived after preimplantation genetic diagnosis: cohort study and meta-analysis. 2017.
107. S. D. McDonald, Z. Han, S. Mulla, K. E. Murphy, J. Beyene, A. Ohlsson and G. Knowledge Synthesis. Preterm birth and low birth weight among in vitro fertilization singletons: a systematic review and meta-analyses. 2009. 146 (2): 138-48.
108. S. D. McDonald, Z. Han, S. Mulla, A. Ohlsson, J. Beyene, K. E. Murphy and G. Knowledge Synthesis. Preterm birth and low birth weight among in vitro fertilization twins: a systematic review and meta-analyses. 2010. 148 (2): 105-13.
109. D. Hvidtjorn, L. Schieve, D. Schendel, B. Jacobsson, C. Svaerke and P. Thorsen. Cerebral palsy, autism spectrum disorders, and developmental delay in children born after assisted conception: a systematic review and meta-analysis. 2009. 163 (1): 72-83.
110. S. Dar, T. Lazer, P. Shah and C. Librach. Singleton pregnancy outcomes after blastocyst versus cleavage stage embryo transfer: A systematic review and meta-analysis. 2013. 100 (3 SUPPL. 1): S248-S249.
111. M. Roque, M. Valle, A. Kostolias, M. Sampaio, S. Geber and M. A. Checa. Fresh versus frozen-thawed embryo transfers: A systematic review and meta-analysis of obstetric outcomes. 2016. 31: i368.
112. B. Velkeniers, A. Van Meerhaeghe, K. Poppe, D. Unuane, H. Tournaye and P. Haentjens. Levothyroxine treatment and pregnancy outcome in women with subclinical hypothyroidism undergoing assisted reproduction technologies: systematic review and meta-analysis of RCTs. 2013. 19 (3): 251-8.
113. M. Dziadosz and M. I. Evans. Re-Thinking Elective Single Embryo Transfer: Increased Risk of Monochorionic Twinning - A Systematic Review. 2017.
114. Hasson J, Limoni D, Malcov M, Frumkin T, Amir H, Shavit T, Bay B, Many A, Almog B. Obstetric and neonatal outcomes of pregnancies conceived after preimplantation genetic diagnosis: cohort study and meta-analysis. Reproductive biomedicine online. 2017. 35(2):208-18.
115. N. van Oostrum, P. De Sutter, J. Meys and H. Verstraelen. Risks associated with bacterial vaginosis in infertility patients: a systematic review and meta-analysis. 2013. 28 (7): 809-15.
116. Y. B. Jeve,​ N. Potdar,​ A. Opoku and M. Khare. Donor oocyte conception and pregnancy complications: a systematic review and meta-analysis. 2016. 123 (9): 1471-80.
117. W. P. Martins,​ C. O. Nastri,​ L. Rienzi,​ S. Z. van der Poel,​ C. R. Gracia and C. Racowsky. Obstetrical and perinatal outcomes following blastocyst transfer compared to cleavage transfer: a systematic review and meta-analysis. 2016. 31 (11): 2561-2569.
118. S. Dar,​ T. Lazer,​ P. S. Shah and C. L. Librach. Neonatal outcomes among singleton births after blastocyst versus cleavage stage embryo transfer: a systematic review and meta-analysis. 2014. 20 (3): 439-48.
119. A. Maheshwari,​ S. Pandey,​ A. Shetty,​ M. Hamilton and S. Bhattacharya. Obstetric and perinatal outcomes in singleton pregnancies resulting from the transfer of frozen thawed versus fresh embryos generated through in vitro fertilization treatment: a systematic review and meta-analysis. 2012. 98 (2): 368-9.
120. L. Sun,​ L. X. Jiang and H. Z. Chen. Obstetric outcome of vanishing twins syndrome: a systematic review and meta-analysis. 2017. 295 (3): .559-567.
121. J. Zhao,​ B. Xu,​ Q. Zhang and Y. P. Li. Which one has a better obstetric and perinatal outcome in singleton pregnancy,​ IVF/ICSI or FET?: a systematic review and meta-analysis. 2016. 14 (1): 51.
122. A. Maheshwari,​ T. Kalampokas,​ J. Davidson and S. Bhattacharya. Obstetric and perinatal outcomes in singleton pregnancies resulting from the transfer of blastocyst-stage versus cleavage-stage embryos generated through in vitro fertilization treatment: a systematic review and meta-analysis. 2013. 100 (6): 1615-10.
123. S. Mastenbroek, M. Twisk, F. van der Veen and S. Repping. Preimplantation genetic screening: A systematic review and meta-analysis of RCTs. 2011. 17 (4): 454-466.
124. A. Pinborg,​ U. B. Wennerholm,​ L. B. Romundstad,​ A. Loft,​ K. Aittomaki,​ V. Soderstrom-Anttila,​ K. G. Nygren,​ J. Hazekamp and C. Bergh. Why do singletons conceived after assisted reproduction technology have adverse perinatal outcome? Systematic review and meta-analysis. 2013. 19 (2): 87-104.
125. Tsoumpou, T. Gelbaya and L. Nardo. The likelihood of live birth and multiple births after single versus multiple embryo transfer: A systematic review and meta-analysis. 2009. 107: S363.
126. Tsoumpou, T. A. Gelbaya and L. G. Nardo. Likelihood of live and multiple births after single versus double embryo transfer in women younger than 36 years: Systematic review and meta-analysis. 2009. 24: i149.

## Full text not available

1. Z.A. Savasan, E. Heasley, A.Abdulahad, A. Zeb. Obstetric and neonatal outcomes in women aged 40 years or older after in vitro fertilization. (the same as the one below?)
2. S. Rakic, N. Zecevic, S. Jankovic-Raznatovic, M. Vasiljevic, R. Anicic. Obstetric and neonatal outcomes in women aged 40 years or older after in vitro fertilization. (says that I do not have access to the article)
3. P. De Sutter, I. Delbaere, J. Gerris and M. Temmerman. [Single embryo transfer]. 2010. 72 (3-4): 137-47.
4. R. M. S. Renuka and D. Gunasheela. Perinatal risks and health outcomes in children born through assisted reproductive technology. 2017. 17 (4): 123-132.
5. Anonymous. "Healthcare Cost, Quality, and Policy: Driving Stakeholder Innovation in Process and Practice". 2014. 21 (1): e116. (this is a collection of abstracts, I’m not sure which one we looked at)
6. M. L. Bonduelle. Long term development of children born after ART. 2009. 24: i83-i84.
7. S. Bhattacharya. Defending single embryo transfer. 2009. 24: i18-i19.
8. Anonymous. [Comparison of pregnancies issued from frozen embryo transfers and pregnancies issued from fresh embryo transfers in fertilization in vitro. FIVNAT]. 1994. 22 (5): 287-91.
9. S. Alur, E. Barrett, D. Li, K. M. Hoeger, M. Wojtowycz, P. Parker and T. Dye. Differences in birth outcomes between Caucasian and african american women undergoing art. 2015. 36.
10. U. B. Wennerholm. Neonatal outcomes following infertility treatments. 2015. 131: E11.
11. V. M. Moore, J. L. Marino, K. J. Willson and M. J. Davies. Perinatal outcomes by infertility treatment modality and subfertility in a population cohort. 2012. 27.
12. Luke and M. B. Brown. The effect of fetal loss on pregnancy outcomes: Lessons from assisted reproductive technology. 2012. 15 (2): 215-216
13. S. Antinori, G. H. Gholami, C. Versaci, M. Antinori, F. Cerusico, S. Antinori and R. Di Nardo. State of art in menopausal pregnancies. 2011. 14: 54-55.
14. Healy. Should fresh et be abandoned in the interests of healthy mothers and babies?. 2009. 49: A11.
15. Jeng, M. MacAluso, N. Larsen and J. Chang. The increased risk of birth outcomes among ART singletons in U.S. is mainly contributed by multiple-fetus pregnancies. 2009. 24: i74-i75.

## Study design: Narrative review

1. L. A. Schieve and M. A. Reynolds. What is the most relevant standard of success in assisted reproduction?: challenges in measuring and reporting success rates for assisted reproductive technology treatments: what is optimal?. 2004. 19 (4): 778-82.
2. A. J. Steel and A. Sutcliffe. Long-term health implications for children conceived by IVF/ICSI. 2009. 12 (1): 21-7.
3. C. Society of Obstetricians annd Gynaecologists of,​ N. Okun and S. Sierra. Pregnancy outcomes after assisted human reproduction. 2014. 36 (1): 64-83.
4. O. Shebl, T. Ebner, A. Sir, M. Sommergruber and G. Tews. The role of mode of conception in the outcome of twin pregnancies. 2009. 61 (2): 141-52.
5. M. Javed, N. Altorairi and H. Alsufyan. Dynamics of a Pregnancy When Two Become Four: A Case Report and Literature Review. 2016. 8 (11): e873.
6. J. J. Kurinczuk. Safety issues in assisted reproduction technology: From theory to reality - Just what are the data telling us about ICSI offspring health and future fertility and should we be concerned?. 2003. 18 (5): 925-931.
7. A. Ferraretti, V. Goossens, S. Bhattacharya, J. A. Castilla, J. De Mouzon, V. Korsak, A. N. Andersen and M. Kupka. Assisted reproductive technology in Europe 2010: Results generated from European registers by ESHRE. Preliminary results. 2013. 28: i38-i39.
8. P. S. Cartwright, D. E. Pittaway, H. W. Jones, 3rd and S. S. Entman. The use of prophylactic antibiotics in obstetrics and gynecology. A review. 1984. 39 (9): 537-54.
9. C. Bouillon and P. Fauque. [Follow-up of children conceived by assisted reproductive technologies]. 2013. 20 (5): 575-9.
10. K. P. Conrad and V. L. Baker. Corpus luteal contribution to maternal pregnancy physiology and outcomes in assisted reproductive technologies. 2013. 304 (2): 69-72.
11. K. J. Barrington and A. Janvier. The paediatric consequences of Assisted Reproductive Technologies, with special emphasis on multiple pregnancies. 2013. 102 (4): 340-8.
12. D. Goldberg, A. Tsafrir, N. Srebnik, M. Gal, E. J. Margalioth, P. Mor, R. Farkash, A. Samueloff and T. Eldar-Geva. How Many Embryos should be Transferred? The Relevance of Parity and Obstetric History. 2016. 18 (6): 313-7.
13. L. P. Reynolds, P. P. Borowicz, C. Palmieri and A. T. Grazul-Bilska. Placental vascular defects in compromised pregnancies: effects of assisted reproductive technologies and other maternal stressors. 2014. 814: 193-204.
14. L. Andreoli, G. K. Bertsias, N. Agmon-Levin, S. Brown, R. Cervera, N. Costedoat-Chalumeau, A. Doria, R. Fischer-Betz, F. Forger, M. F. Moraes-Fontes, M. Khamashta, J. King, A. Lojacono, F. Marchiori, P. L. Meroni, M. Mosca, M. Motta, M. Ostensen, C. Pamfil, L. Raio, M. Schneider, E. Svenungsson, M. Tektonidou, S. Yavuz, D. Boumpas and A. Tincani. EULAR recommendations for women's health and the management of family planning, assisted reproduction, pregnancy and menopause in patients with systemic lupus erythematosus and/or antiphospholipid syndrome. 2017. 76 (3): 476-485.
15. E. Garalejic. Polycystic ovary syndrome-infertility treatment and pregnancy outcome. 2015. 131: E69.
16. Anonymous. Pregnancies and births resulting from in vitro fertilization: French national registry,​ analysis of data 1986 to 1990. FIVNAT (French In Vitro National). 1995. 64 (4): 746-56.
17. A. A. Nardelli, T. Stafinski, T. Motan, K. Klein and D. Menon. Assisted reproductive technologies (ARTs): Evaluation of evidence to support public policy development. 2014. 11 (1).
18. S. Bhattacharya. Does it affect the mother and the offspring?. 2016. 31: i93-i94.
19. J. De Mouzon, V. Goossens, S. Bhattacharya, J. A. Castilla, A. P. Ferraretti, V. Korsak, M. Kupka, K. G. Nygren and A. Nyboe Andersen. Assisted reproductive technology in Europe, 2008: Results generated from European registers by ESHRE. Preliminary results. 2011. 26: i84.
20. D. F. Albertini, J. L. H. Evers, J. P. M. Geraedts, L. Gianaroli, R. Sharpe, K. D. Sinclair, A. Sunde, A. Van Steirteghem, D. T. Baird, P. G. Crosignani, P. Devroey, K. Diedrich, B. C. J.M. Fauser, L. Fraser, J. S. Tapanainen, B. Tarlatzis and A. Veiga. Birth defects and congenital health risks in children conceived through assisted reproduction technology (ART): A meeting report. 2014. 31 (8): 947-958.
21. R. Tsukinoki and Y. Murakami. Non-communicable disease epidemic: epidemiology in action (EuroEpi 2013 and NordicEpi 2013): Aarhus, Denmark from 11 August to 14 August 2013. 2013. 28 (1): 1-270.
22. A. Venn and J. Lumley. Births after a period of infertility in Victorian women 1982-1990. 1993. 33 (4): 379-84.
23. V. Grigorescu, Y. Zhang, D. Kissin, E. Sauber-Schatz, M. Sunderam, R. Kirby, H. Diop, P. McKane and D. Jamieson. Maternal characteristics and pregnancy outcomes among women undergoing Assisted Reproductive Technology (ART) by infertility diagnosis: Polycystic ovary syndrome versus tubal obstruction. 2013. 28: i8-i9.
24. B. Luke. Pregnancy and birth outcomes in couples with infertility with and without assisted reproductive technology: with an emphasis on US population-based studies. 2017.
25. M. Carolan. Maternal age >=45 years and maternal and perinatal outcomes: a review of the evidence. 2013. 29 (5).
26. N. Okun,​ S. Sierra,​ C. Genetics and C. Special. Pregnancy outcomes after assisted human reproduction. 2014. 36 (1): 64-83.
27. P. Rufat,​ F. Olivennes,​ J. de Mouzon,​ M. Dehan and R. Frydman. Task force report on the outcome of pregnancies and children conceived by in vitro fertilization (France: 1987 to 1989). 1994. 61 (2): 324-30.
28. V. Bonduelle,​ P. Braude,​ P. Devroey,​ J. L. H. Evers,​ B. C. J. M. Fauser,​ I. Liebaers,​ G. D. Palermo,​ A. Templeto,​ D. T. Baird,​ J. Cohen,​ E. Diczfalusy,​ K. Diedrich,​ L. Fraser,​ L. Gianaroli,​ A. Glasier,​ G. Ragni,​ A. Sunde,​ B. Tarlatzis,​ A. Van Steirteghem,​ J. Collins and P. G. Crosignani. Intracytoplasmic sperm injection (ICSI) in 2006: Evidence and evolution. 2007. 13 (6): 515-526.
29. Z. Pandian,​ J. Marjoribanks,​ O. Ozturk,​ G. Serour,​ S. Bhattacharya and R. Cochrane Database of Systematic. Number of embryos for transfer following in vitro fertilisation or intra-cytoplasmic sperm injection. 2013.
30. M. Chen and L. K. Heilbronn. The health outcomes of human offspring conceived by assisted reproductive technologies (ART). 2017. 1-15.
31. M. S. Kamath and S. K. Sunkara. Perinatal outcomes after oocyte donation and in-vitro fertilization. 2017. 29 (3): 126-130.
32. S. Bhattacharya. Maternal and perinatal outcomes after fresh versus frozen embryo transfer-what is the risk-benefit ratio?. 2016. 106 (2): 241-3.
33. E. Turkgeldi, H. Yagmur, A. Seyhan, B. Urman and B. Ata. Short and long term outcomes of children conceived with assisted reproductive technology. 2016. 207: 129-136.
34. A. Maheshwari, M. Hamilton and S. Bhattacharya. Should we be promoting embryo transfer at blastocyst stage?. 2016. 32 (2): 142-6.
35. B. Luke, J. E. Stern, M. D. Hornstein, M. Kotelchuck, H. Diop, H. Cabral and E. R. Declercq. Is the wrong question being asked in infertility research?. 2016. 33 (1): 3-8.
36. R. Weinerman and M. Mainigi. Why we should transfer frozen instead of fresh embryos: the translational rationale. 2014. 102 (1): 10-8.
37. B. S. Shapiro, S. T. Daneshmand, F. C. Garner, M. Aguirre and C. Hudson. Clinical rationale for cryopreservation of entire embryo cohorts in lieu of fresh transfer. 2014. 102 (1): 3-9.
38. A.-K. A. Henningsen and A. Pinborg. Birth and perinatal outcomes and complications for babies conceived following ART. 2014. 19 (4): 234-8.
39. M. Hansen and C. Bower. The impact of assisted reproductive technologies on intra-uterine growth and birth defects in singletons. 2014. 19 (4): 228-33.
40. E. Groeneveld, M. J. Lambers, C. B. Lambalk, K. A. Broeze, M. Haapsamo, P. de Sutter, B. C. Schoot, R. Schats, B. W. J. Mol and P. G. A. Hompes. Preconceptional low-dose aspirin for the prevention of hypertensive pregnancy complications and preterm delivery after IVF: a meta-analysis with individual patient data. 2013. 28 (6): 1480-8.
41. L. A. Kondapalli and A. Perales-Puchalt. Low birth weight: is it related to assisted reproductive technology or underlying infertility?. 2013. 99 (2): 303-10.
42. K. T. Barnhart. Assisted reproductive technologies and perinatal morbidity: interrogating the association. 2013. 99 (2): 299-302.
43. G. Neuman and G. Koren. Mothersisk update: reproductive outcomes after assisted conception. 2013. 59 (1): 33-36.
44. U. Zollner and J. Dietl. Perinatal risks after IVF and ICSI. 2013. 41 (1): 17-22.
45. V. S. Talaulikar and S. Arulkumaran. Reproductive outcomes after assisted conception. 2012. 67 (9): 566-83.
46. C. Bergh and U.-B. Wennerholm. Obstetric outcome and long-term follow up of children conceived through assisted reproduction. 2012. 26 (6): 841-52.
47. J. Arendt, C. Schilling, M. Peiffer, S. Ginter, A. F. Nahanb, C. Lemosb, M. Duboisb, F. Thononb, C. Jouanb, O. Gaspardb, M. E. Larcher, V. Gomez, S. Pereira and M. Geimer. [Retrospective studies of pregnancies after assisted medical reproduction from 2001-2009 and Central Hospital in Luxembourg (part 2)]. 2010. (2): 257-70.
48. P. Poikkeus and A. Tiitinen. Does single embryo transfer improve the obstetric and neonatal outcome of singleton pregnancy?. 2008. 87 (9): 888-92.
49. W. Ombelet, M. Camus and L. de Catte. Relative contribution of ovarian stimulation versus in vitro fertilization and intracytoplasmic sperm injection to multifetal pregnancies requiring reduction to twins. 2007. 88 (4): 997-9.
50. E. C. W. Group. Intracytoplasmic sperm injection (ICSI) in 2006: evidence and evolution. 2007. 13 (6): 515-26.
51. U. M. Reddy, R. J. Wapner, R. W. Rebar and R. J. Tasca. Infertility, assisted reproductive technology, and adverse pregnancy outcomes: executive summary of a National Institute of Child Health and Human Development workshop. 2007. 109 (4): 967-77.
52. N. Mukhopadhaya and S. Arulkumaran. Reproductive outcomes after in-vitro fertilization. 2007. 19 (2): 113-9.
53. I. Blickstein. Does assisted reproduction technology, per se, increase the risk of preterm birth?. 2006. 113 Suppl 3: il68-71.
54. A. P. Cheung. Assisted reproductive technology: both sides now. 2006. 51 (4): 283-92.
55. M. Ludwig. Risk during pregnancy and birth after assisted reproductive technologies: an integral view of the problem. 2005. 23 (4): 363-70.
56. L. A. Schieve, S. A. Rasmussen, G. M. Buck, D. E. Schendel, M. A. Reynolds and V. C. Wright. Are children born after assisted reproductive technology at increased risk for adverse health outcomes?. 2004. 103 (6): 1154-63.
57. R. D. Lambert. Safety issues in assisted reproductive technology: aetiology of health problems in singleton ART babies. 2003. 18 (10): 1987-91.
58. F. Olivennes. Patient-friendly ovarian stimulation. 2003. 7 (1): 30-4.
59. A. Gutierrez Najar, J. Stern Colin Y Nunes, M. E. Gonzalez Panzzi and M. Orbea Travez. Pregnancy and birth after assisted reproduction. 2002. 5 (1): 78-88.
60. M. Ludwig and K. Diedrich. Follow-up of children born after assisted reproductive technologies. 2002. 5 (3): 317-22.
61. F. Olivennes, R. Fanchin, N. Ledee, C. Righini, I. J. Kadoch and R. Frydman. Perinatal outcome and developmental studies on children born after IVF. 2002. 8 (2): 117-28.
62. J. L. Simpson and D. J. Lamb. Genetic effects of intracytoplasmic sperm injection. 2001. 19 (3): 239-49.
63. F. Olivennes. [ITwin pregnancy after treatment for infertility: failure or success? A failure]. 2001. 29 (3): 257-60.
64. B. C. Tarlatzis and G. Grimbizis. Pregnancy and child outcome after assisted reproduction techniques. 1999. 14 Suppl 1: 231-42.
65. S. E. Buitendijk. Children after in vitro fertilization. An overview of the literature. 1999. 15 (1): 52-65.
66. M. Dehan. [The role of infertility treatment in very premature births in France. The point of view of the neonatologist]. 1998. 26 (7-8): 512-6.
67. V. Y. Yu. Assisted reproduction technology, multiple births, and adverse perinatal outcome. 1998. 39 (2): 208-11.
68. P. A. Lancaster. Registers of in-vitro fertilization and assisted conception. 1996. 11 Suppl 4: 89-9.
69. F. M. Helmerhorst and M. J. N. C. Keirse. Assisted reproductive technology and pregnancy outcomes. 2016. 123 (8): 1329.
70. O. Irion and N. Fournet Irion. Pregnancy care in women older than 40 years and after egg donation. 2015. 11 (456-457): 68-71.
71. E. Somigliana, L. Benaglia, A. Paffoni, A. Busnelli, P. Vigano and P. Vercellini. Risks of conservative management in women with ovarian endometriomas undergoing IVF. 2014. 21 (4): 486-499.
72. Y. Shufaro and J. G. Schenker. The risks and outcome of pregnancy in an advanced maternal age in oocyte donation cycles. 2014. 27 (16): 1703-1709.
73. J. Evans, N. J. Hannan, T. A. Edgell, B. J. Vollenhoven, P. J. Lutjen, T. Osianlis, L. A. Salamonsen and L. J. F. Rombauts. Fresh versus frozen embryo transfer: Backing clinical decisions with scientific and clinical evidence. 2014. 20 (6): 808-821.
74. S. R. Tandulwadkar, P. A. Lodha and N. T. Mangeshikar. Obstetric complications in women with IVF conceived pregnancies and polycystic ovarian syndrome. 2014. 7 (1): 13-18.
75. C. E. M. Aiken and J. C. Brockelsby. Fetal and maternal consequences of pregnancies conceived using art. 2014. 25 (3-4): 281-294.
76. L. P. Reynolds, K. A. Vonnahme, C. O. Lemley, D. A. Redmer, A. T. Grazul-Bilska, P. P. Borowicz and J. S. Caton. Maternal stress and placental vascular function and remodeling. 2013. 11 (5): 564-593.
77. S. L. Dovey. Oocyte cryopreservation: Advances and drawbacks. 2012. 64 (6): 485-500.
78. Anonymous. In vitro fertilisation: Perinatal risks and early childhood outcomes. 2012. 15 (2): 62-68.
79. L. Ismail, M. Mittal and E. Kalu. IVF twins: Buy one get one free?. 2012. 38 (4): 252-257.
80. M. Metwally and W. L. Ledger. Long-term complications of assisted reproductive technologies. 2011. 14 (2): 77-87.
81. R. Merchant, G. Gandhi and G. N. Allahbadia. In vitro fertilization/intracytoplasmic sperm injection for male infertility. 2011. 27 (1): 121-132.
82. S. K. Kalra and K. T. Barnhart. In vitro fertilization and adverse childhood outcomes: What we know, where we are going, and how we will get there. A glimpse into what lies behind and beckons ahead. 2011. 95 (6) 1887-1889-.
83. K. G. Michalakis, T. B. Mesen, L. M. Brayboy, B. Yu, K. S. Richter, M. Levy, E. Widra and J. H. Segars. Subclinical elevations of thyroid-stimulating hormone and assisted reproductive technology outcomes. 2011. 95 (8): 2634-2637.
84. M. L. P. van der Hoorn, E. E. L. O. Lashley, D. W. Bianchi, F. H. J. Claas, C. M. C. Schonkeren and S. A. Scherjon. Clinical and immunologic aspects of egg donation pregnancies: A systematic review. 2010. 16 (6): 704-712.
85. E. Basatemur and A. Sutcliffe. Follow-up of Children Born after ART. 2008. 29 (SUPPL.2): 135-140.
86. A. Pinborg. The vanishing twin: Prevalence and consequences for outcome after assisted reproduction. 2008. 3 (3): 369-377.
87. J. S. Hesla and W. B. Schoolcraft. Treatment of idiopathic infertility with assisted reproductive technologies. 1997. 8 (4): 665-687.
88. Luke B. Pregnancy and birth outcomes in couples with infertility with and without assisted reproductive technology: with an emphasis on US population-based studies. American journal of obstetrics and gynecology. 2017. 217(3):270-81.
89. Kamath MS, Sunkara SK. Perinatal outcomes after oocyte donation and in-vitro fertilization. Current Opinion in Obstetrics and Gynecology. 2017. 29(3):126-30.

## Study design: Other (e.g. conferences, case report, case series, editorial, commentary, guidelines, survey, surveyécross-sectional, case-control, clinical trials)

1. N. R. Saunders, J. Hellmann and D. Farine. Cerebral Palsy and Assisted Conception. 2011. 33 (10): 1038-1043.
2. Mahecha-Reyes E.; Grillo-Ardila C.F. Maternal factors associated with low birth weight in term neonates: A case-controlled study. 2018. International Journal of Gynecology and Obstetrics. 143 Supplement 3: 682.
3. J. Figueras-Aloy, G. Sebastiani, A. Pertierra Cortada, E. Vidal Sorde and J. Balasch Cortina. Factors related with assisted reproductive technologies and neonatal outcomes. 2010. 23: 24-.
4. K. G. Nygren. Improvements of IVF safety in Sweden with SET as the norm. 2011. 26: i20.
5. J. Johnston. Judging octomom. 2009. 39 (3): 23-24.
6. E. A. Sullivan. Simply the best? Births following assisted reproductive technology in the New Millenium. 2010. 50: 32-33.
7. J. De Mouzon. Assisted reproductive tehcnology in Europe, 2006. Results generated from European registers by ESHRE. 2010. 25: i80.
8. J. C. Monnier. Follow-up of pregnancies after in-vitro fertilization. 1986. 14 (11): 993-997.
9. G. Haan, R. E. Bernardus, J. M. G. Hollanders, R. A. Leerentveld and F. M. N. Prak Naaktgeboren. Results of IVF from a prospective multicentre study. 1991. 6 (6): 805-810.
10. N. Todorovic, V. Ciric Ljubinkovic, B. Jovanovic, M. Nikolic, O. Stanojlovic, J. Durutovic, N. Jovanovic, J. Djorovic, S. Tasic, V. Stojanovic and A. Mladenovic Mihailovic. Perinatal outcome of art. 2010. 23: 272.
11. I. Molina Gonzalez, A. Clavero Gilabert, M. C. Gonzalvo Lopez, A. Rosales Martinez, L. Martinez Navarro, J. Mozas Moreno and J. A. Castilla Alcala. Assisted reproduction techniques for patients with infectious diseases. 2011. 26: i275.
12. G. Ricci, R. Boscolo, M. Martinelli and L. Fischer-Tamaro. Tubal factor infertility and perinatal risk after assisted reproductive technology. 2013. 122 (4): 908-909.
13. V. S. Talaulikar and S. Arulkumaran. Maternal, perinatal and long-term outcomes after assisted reproductive techniques (ART): Implications for clinical practice. 2013. 170 (1): 13-19.
14. L. Bialystok, N. Poole and L. Greaves. Preconception care: Call for national guidelines. 2013. 59 (10): 1037-e437.
15. R. S. Howe, R. A. Sayegh, K. L. Durinzi and R. W. Tureck. Perinatal outcome of singleton pregnancies conceived by in vitro fertilization: a controlled study. 1990. 10 (3): 261-6.
16. S. Friedler, S. Mashiach and N. Laufer. Births in Israel resulting from in-vitro fertilization/embryo transfer, 1982-1989: National Registry of the Israeli Association for Fertility Research. 1992. 7 (8): 1159-63.
17. J. G. Schenker and Y. Ezra. Complications of assisted reproductive techniques. 1994. 61 (3): 411-22.
18. P. Doyle. The U.K. Human Fertilisation and Embryology Authority. How it has contributed to the evaluation of assisted reproduction technology. 1999. 15 (1): 3-10.
19. C. Centers for Disease and Prevention. From the Centers of Disease Control and Prevention. Use of assisted reproductive technology--United States, 1996 and 1998. 2002. 51 (5): 97-101.
20. L. S. Bakketeig, H. J. Hoffman and E. E. Harley. The tendency to repeat gestational age and birth weight in successive births. 1979. 135 (8): 1086-103.
21. M. Roque. Towards a "freeze all" policy. 2015. 30: i62.
22. J. Vikstrom, M. Hammar, A. Josefsson, M. Bladh and G. Sydsjo. Birth characteristics in a clinical sample of women seeking infertility treatment: a case-control study. 2014. 4 (3): e004197.
23. R. Isaksson, M. Gissler and A. Tiitinen. Obstetric outcome among women with unexplained infertility after IVF: A matched case-control study. 2002. 17 (7): 1755-1761.
24. O. J. Carpinello, R. S. Raj, P. R. Casson and C. A. Jones. Outcomes and incremental costs following 1, 2, or3+ embryo transfers in in-vitro fertilization pregnancies: A vermont experience. 2013. 100 (3 SUPPL. 1): S288.
25. S. Harbottle, C. Hughes, R. Cutting, S. Roberts, D. Brison, E. Association Of Clinical and S. The British Fertility. Elective Single Embryo Transfer: an update to UK Best Practice Guidelines. 2015. 18 (3): 165-83.
26. Anonymous. Risks of assisted reproduction. 1998. 7 (33): 27-8.
27. C. De Geyter. Assisted reproductive medicine in Switzerland. 2012. 142: 3569.
28. A. R. Han,​ H. O. Kim,​ S. W. Cha,​ C. W. Park,​ J. Y. Kim,​ K. M. Yang,​ I. O. Song,​ M. K. Koong and I. S. Kang. Adverse pregnancy outcomes with assisted reproductive technology in non-obese women with polycystic ovary syndrome: a case-control study. 2011. 38 (2): 103-8.
29. L. F. Watson, J.-A. Rayner, J. King, D. Jolley and D. Forster. Intracervical procedures and the risk of subsequent very preterm birth: a case-control study. 2012. 91 (2): 204-10.
30. B. Kallen, O. Finnstrom, A. Lindam, E. Nilsson, K. G. Nygren and P. Otterblad Olausson. Trends in delivery and neonatal outcome after in vitro fertilization in Sweden: data for 25 years. 2010. 25 (4): 1026-34.
31. A. G. Adesiyun and E. Eseigbe. Triplet gestation: clinical outcome of 14 cases. 2007. 6 (1): 12-6.
32. B. Johnson and W. Chavkin. Policy efforts to prevent ART-related preterm birth. 2007. 11 (3): 219-25.
33. J. Halliday. Outcomes of IVF conceptions: are they different?. 2007. 21 (1): 67-81.
34. Aleksanyan A. COMPLICATIONS OF PREGNANCY, RESULTING FROM ASSISTED REPRODUCTIVE TECHNOLOGY. Georgian medical news. 2017. (268-269):63-6
35. J. Johnston, M. K. Gusmano and P. Patrizio. Preterm births, multiples, and fertility treatment: recommendations for changes to policy and clinical practices. 2014. 102 (1): 36-9.
36. J. K. H. Tan, E. L. Tan, D. Kanagalingam, S. L. Yu and L. K. Tan. Multiple pregnancy is the leading contributor to cesarean sections in in vitro fertilization pregnancies: An analysis using the Robson 10-group classification system. 2016. 42 (9): 1141-5.
37. F. M. Ubaldi, A. Capalbo, S. Colamaria, S. Ferrero, R. Maggiulli, G. Vajta, F. Sapienza, D. Cimadomo, M. Giuliani, E. Gravotta, A. Vaiarelli and L. Rienzi. Reduction of multiple pregnancies in the advanced maternal age population after implementation of an elective single embryo transfer policy coupled with enhanced embryo selection: pre- and post-intervention study. 2015. 30 (9): 2097-106.
38. Oron, T. Sokal-Arnon, W. Y. Son, D. Nayot, T. Tulandi and H. Holzer. Does the transfer day of single embryos affect the birth weight?. 2013. 100 (3 SUPPL. 1): S97F. Davari Tanha. Stimulation of the endometrium with high-grade blastocyst culture supernatant (SEHB) can improve pregnancy outcome for couples undergoing intracytoplasmic sperm injection (ICSI): A randomized clinical trial. 2012. 6: 38-39.
39. K. L. Bowen. Outcomes and costs associated with fertility drug therapy in an insured population with a state fertility mandate. 2009. 12 (3): A170.
40. Lavie A, Alshiek J, Avraham S, Almog B, Levin I. 776: Obstetric and gynecological outcomes of women w/wo ovarian hyperstimulation syndrome following IVF-A case-control study. American Journal of Obstetrics & Gynecology. 2018. 218(1):S463-4
41. G. Oron,​ D. Nayot,​ W.-Y. Son,​ H. Holzer,​ W. Buckett and T. Tulandi. Obstetric and perinatal outcome from single cleavage transfer and single blastocyst transfer: a matched case-control study. 2015. 31 (6): 469-72.
42. P. De Sutter. Single embryo transfer (set) not only leads to a reduction in twinning rates after IVF/ICSI, but also improves obstetrical and perinatal outcome of singletons. 2006. 68 (5-6): 319-27.
43. H. Logerot-Lebrun,​ J. De Mouzon,​ A. Hachelot and A. Spira. Pregnancies and births resulting from in vitro fertilization: French national registry,​ analysis of data 1986 to 1990. 1995. 64 (4): 746-756.
44. H. Nishio,​ T. Fujii,​ J. Sugiyama,​ N. Kuji,​ M. Tanaka,​ T. Hamatani,​ K. Miyakoshi,​ K. Minegishi,​ H. Tsuda,​ T. Iwata,​ K. Tanaka,​ T. Fukuchi,​ Y. Takehara,​ Y. Yoshimura and D. Aoki. Reproductive and obstetric outcomes after radical abdominal trachelectomy for early-stage cervical cancer in a series of 31 pregnancies. 2013. 28 (7): 1793-8.
45. J. Barrat and D. Leger. [The outcome in pregnancies resulting after the induction of ovulation 519 pregnancies (author's transl)]. 1979. 8 (4): 333-42.
46. J. X. Wang,​ R. J. Norman and P. Kristiansson. The effect of various infertility treatments on the risk of preterm birth. 2002. 17 (4): 945-9.
47. P. Poikkeus,​ L. Unkila-Kallio,​ S. Vilska,​ L. Repokari,​ R. L. Punamaki,​ A. Aitokallio-Tallberg,​ J. Sinkkonen,​ F. Almqvist,​ M. Tulppala and A. Tiitinen. Impact of infertility characteristics and treatment modalities on singleton pregnancies after assisted reproduction. 2006. 13 (1): 135-44.
48. V. Addor,​ B. Santos-Eggimann,​ C. L. Fawer,​ F. Paccaud and A. Calame. Impact of infertility treatments on the health of newborns. 1998. 69 (2): 210-5.
49. V. C. Wright,​ J. Chang,​ G. Jeng,​ M. Macaluso,​ C. Centers for Disease and Prevention. Assisted reproductive technology surveillance--United States,​ 2005. 2008. 57 (5): 1-23.
50. Y. Tada,​ K. Kitaya,​ N. Amano,​ M. Kobatake,​ T. Hayashi,​ S. Taguchi,​ M. Funabiki and Y. Nakamura. A pilot survey on obstetric complications in pregnant women with a history of repeated embryo implantation failure and those undergoing single local endometrial injury. 2015. 42 (2): 176-8.
51. Roalier, A., Bachelot, A., Rufat, P., & Logerot, H. (1993). Evaluation of FIVNAT. 992. Contraception, fertilite, sexualite (1992), 21(5), 354-357.
52. K. M. Perkins,​ S. L. Boulet,​ D. J. Jamieson,​ D. M. Kissin and G. National Assisted Reproductive Technology Surveillance System. Trends and outcomes of gestational surrogacy in the United States. 2016. 106 (2):.435-442.e2.
53. R. M. Kermani,​ B. Allahverdi,​ H. Gourabi,​ J. Koohpayezade,​ M. R. Nateghi and S. Dadashloo. Perinatal outcomes of newborn infants conceived by assisted reproductive techniques in Royan Institute. 2009. 3 (2): 62-65.
54. P. A. Lancaster. Obstetric outcome. 1985. 12 (4): 847-64.
55. J. Fedder,​ A. Loft,​ E. T. Parner,​ S. Rasmussen and A. Pinborg. Neonatal outcome and congenital malformations in children born after ICSI with testicular or epididymal sperm: a controlled national cohort study. 2013. 28 (1): 230-40.
56. I. Tsoumpou,​ A. M. Mohamed,​ C. Tower,​ S. A. Roberts and L. G. Nardo. Failed IVF cycles and the risk of subsequent preeclampsia or fetal growth restriction: A case-control exploratory study. 2011. 95 (3): 973-978.
57. Anonymous. Births in Great Britain resulting from assisted conception,​ 1978-87. MRC Working Party on Children Conceived by In Vitro Fertilisation. 1990. 300 (6734): 1229-33.
58. H. L. T. Wan,​ P. W. Hui,​ H. W. R. Li and E. H. Y. Ng. Obstetric outcomes in women with polycystic ovary syndrome and isolated polycystic ovaries undergoing in vitro fertilization: A retrospective cohort analysis. 2015. 28 (4): 475-478.
59. E. A. Sullivan,​ F. Zegers-Hochschild,​ R. Mansour,​ O. Ishihara,​ J. de Mouzon,​ K. G. Nygren and G. D. Adamson. International Committee for Monitoring Assisted Reproductive Technologies (ICMART) world report: assisted reproductive technology 2004. 2013. 28 (5): 1375-90.
60. A. Sazonova, K. Kallen, A. Thurin-Kjellberg, U. B. Wennerholm and C. Bergh. Neonatal and maternal outcomes comparing women undergoing two in vitro fertilization (IVF) singleton pregnancies and women undergoing one IVF twin pregnancy: Editorial comment. 2013. 68 (6): 423-425.
61. E. J. Forman, M. D. Werner and R. T. Scott. Extended culture and the risk of preterm delivery in singletons: confounding by indication?. 2013. 28 (7): 2021.
62. E. I. Kamphuis, F. J. Hermans and B. W. Mol. Tubal factor infertility and perinatal risk after assisted reproductive technology. 2013. 122 (4): 908.
63. K. G. Nygren. Thirty years now - But can we say IVF is safe? On the clinical significance and durability of current knowledge. 2010. 7 (4): 225-228.
64. M. P. Umstad and M. J. Gronow. Multiple pregnancy: A modern epidemic?. 2003. 178 (12): 613-615.
65. A. P. Booth, M. McKibbin and T. R. Dabbs. Swedish in-vitro fertilisation study. 2000. 355 (9206): 845-846.
66. S. W. D'Souza, B. Richards and B. A. Lieberman. Swedish in-vitro fertilisation study. 2000. 355 (9206): 846-847.
67. Davies MJ, Rumbold AR, Moore VM. Assisted reproductive technologies: a hierarchy of risks for conception, pregnancy outcomes and treatment decisions. Journal of developmental origins of health and disease. 2017. 8(4):443-7.
68. J. S. Refuerzo. Impact of multiple births on late and moderate prematurity. 2012. 17 (3): 143-5.
69. Amini P, Maroufizadeh S, Samani RO, Hamidi O, Sepidarkish M. Prevalence and determinants of preterm birth in Tehran, Iran: a comparison between logistic regression and decision tree methods. Osong public health and research perspectives. 2017. 8(3):195.
70. Brock CO, Gyamfi-Bannerman C. Assisted Reproductive Technology and Preterm Delivery in Twin Gestations. Reproductive Sciences. 2017. 24: 248A-248A.
71. Aflatoonian A, Mansoori-Torshizi M, Mojtahedi MF, Aflatoonian B, Khalili MA, Amir-Arjmand MH, Soleimani M, Aflatoonian N, Oskouian H, Tabibnejad N, Humaidan P. Fresh versus frozen embryo transfer after gonadotropin-releasing hormone agonist trigger in gonadotropin-releasing hormone antagonist cycles among high responder women: A randomized, multi-center study. International Journal of Reproductive Biomedicine. 2018. 16(1):9.
72. Cavoretto P, Candiani M, Giorgione V, Inversetti A, Abu‐Saba MM, Tiberio F, Sigismondi C, Farina A. Risk of spontaneous preterm birth in singleton pregnancies conceived after IVF/ICSI treatment: meta‐analysis of cohort studies. Ultrasound in Obstetrics & Gynecology. 2018 Jan 1;51(1):43-53.
73. Ogawa K, Urayama KY, Tanigaki S, Sago H, Sato S, Saito S, Morisaki N. Association between very advanced maternal age and adverse pregnancy outcomes: a cross sectional Japanese study. BMC pregnancy and childbirth. 2017. 17(1):349.
74. Halimi AA, Safari S, Parvareshi HM. Epidemiology and Related Risk Factors of Preterm Labor as an obstetrics emergency. Emergency (Tehran, Iran). 2017. 5(1): e3.
75. Zhang B, Wei D, Legro RS, Shi Y, Li J, Zhang L, Hong Y, Sun G, Zhang T, Li W, Chen ZJ. Obstetric complications after frozen versus fresh embryo transfer in women with polycystic ovary syndrome: results from a randomized trial. Fertility and sterility. 2018. 109(2):324-9.

## Overlapped cohort

1. Glavind MT, Forman A, Arendt LH, Nielsen K, Henriksen TB. Endometriosis and pregnancy complications: a Danish cohort study. Fertility and sterility. 2017. 107(1):160-6. (Danish) Wennerholm, 2013
2. N. K. Tepper,​ S. L. Farr,​ B. B. Cohen,​ A. Nannini,​ Z. Zhang,​ J. E. Anderson,​ D. J. Jamieson and M. Macaluso. Singleton preterm birth: risk factors and association with assisted reproductive technology. 2012. 16 (4): 807-13. (Massachusetts) Luke, 2018
3. G. L. Dunietz,​ C. Holzman,​ P. McKane,​ C. Li,​ S. L. Boulet,​ D. Todem,​ D. M. Kissin,​ G. Copeland,​ D. Bernson,​ W. M. Sappenfield and M. P. Diamond. Assisted reproductive technology and the risk of preterm birth among primiparas. 2015. 103 (4): 974-979.e1. (Massachusetts) Luke, 2018
4. S. Luke,​ W. M. Sappenfield,​ R. S. Kirby,​ P. McKane,​ D. Bernson,​ Y. Zhang,​ F. Chuong,​ B. Cohen,​ S. L. Boulet and D. M. Kissin. The Impact of ART on Live Birth Outcomes: Differing Experiences across Three States. 2016. 30 (3): 209-16. (Massachusetts) Luke, 2018
5. Luke B, Gopal D, Cabral H, Stern JE, Diop H. Pregnancy, birth, and infant outcomes by maternal fertility status: the Massachusetts Outcomes Study of Assisted Reproductive Technology. American journal of obstetrics and gynecology. 2017. 217(3):327-e1. (Massachusetts) Luke, 2018
6. L. A. Schieve,​ B. Cohen,​ A. Nannini,​ C. Ferre,​ M. A. Reynolds,​ Z. Zhang,​ G. Jeng,​ M. Macaluso,​ V. C. Wright and R. Massachusetts Consortium for Assisted Reproductive Technology Epidemiologic. A population-based study of maternal and perinatal outcomes associated with assisted reproductive technology in Massachusetts. 2007. 11 (6): 517-25. (Massachusetts) Luke, 2018
7. T. A. Merritt, M. Goldstein, R. Philips, R. Peverini, J. Iwakoshi, A. Rodriguez and B. Oshiro. Impact of ART on pregnancies in California: an analysis of maternity outcomes and insights into the added burden of neonatal intensive care. 2014. 34 (5): 345-350.(California) Luke, 2018
8. B. Luke,​ J. E. Stern,​ M. Kotelchuck,​ E. R. Declercq,​ B. Cohen and H. Diop. Birth Outcomes by Infertility Diagnosis Analyses of the Massachusetts Outcomes Study of Assisted Reproductive Technologies (MOSART). 2015. 60 (11-12): 480-90. (Massachusetts) Luke, 2018
9. B. Luke,​ J. E. Stern,​ M. Kotelchuck,​ E. R. Declercq,​ M. Anderka and H. Diop. Birth Outcomes by Infertility Treatment: Analyses of the Population-Based Cohort: Massachusetts Outcomes Study of Assisted Reproductive Technologies (MOSART). 2016. 61 (3-4): 114-27. (Massachusetts) Luke, 2018
10. B. Kallen,​ O. Finnstrom,​ K.-G. Nygren and P. O. Olausson. In vitro fertilization (IVF) in Sweden: infant outcome after different IVF fertilization methods. 2005. 84 (3): 611-7. (Sweden) Wennerholm, 2013
11. B. Luke,​ D. Gopal,​ H. Cabral,​ J. E. Stern and H. Diop. Pregnancy,​ birth,​ and infant outcomes by maternal fertility status: the Massachusetts Outcomes Study of Assisted Reproductive Technology. 2017. (Massachusetts) Luke, 2018
12. E. Declercq, B. Luke, C. Belanoff, H. Cabral, H. Diop, D. Gopal, L. Hoang, M. Kotelchuck, J. E. Stern and M. D. Hornstein. Perinatal outcomes associated with assisted reproductive technology: the Massachusetts Outcomes Study of Assisted Reproductive Technologies (MOSART). 2015. 103 (4): 888-95. Luke, 2018
13. Stern JE, Liu CL, Cabral HJ, Richards EG, Coddington CC, Hwang S, Dukhovny D, Diop H, Missmer SA. Birth outcomes of singleton vaginal deliveries to ART-treated, subfertile, and fertile primiparous women. Journal of assisted reproduction and genetics. 2018. 35(9):1585-93. (Massachusetts) Luke, 2018
14. Wang YA, Sullivan EA, Black D, Dean J, Bryant J, Chapman M. Preterm birth and low birth weight after assisted reproductive technology-related pregnancy in Australia between 1996 and 2000. Fertility and sterility. 2005. 83(6):1650-8. (Australia) Davies, 2012
15. Dunietz GL, Holzman C, Zhang Y, Li C, Todem D, Boulet SL, McKane P, Kissin DM, Copeland G, Bernson D, Diamond MP. Assisted reproduction and risk of preterm birth in singletons by infertility diagnoses and treatment modalities: a population-based study. Journal of assisted reproduction and genetics. 2017. 34(11):1529-35 (Massachusetts,Florida, and Michigan) Luke, 2018
16. Luke, B., Gopal, D., Cabral, H., Stern, J. E., & Diop, H. (2017). Pregnancy, birth, and infant outcomes by maternal fertility status: the Massachusetts Outcomes Study of Assisted Reproductive Technology. American journal of obstetrics and gynecology, 217(3), 327-e1 (Massachusetts) Luke, 2018
17. U. B. Wennerholm,​ L. Hamberger,​ L. Nilsson,​ M. Wennergren,​ M. Wikland and C. Bergh. Obstetric and perinatal outcome of children conceived from cryopreserved embryos. 1997. 12 (8): 1819-25. (Sweden) Wennerholm, 2013
18. Martin AS, Chang J, Zhang Y, Kawwass JF, Boulet SL, McKane P, Bernson D, Kissin DM, Jamieson DJ, Mneimneh AS, Sunderam S. Perinatal outcomes among singletons after assisted reproductive technology with single-embryo or double-embryo transfer versus no assisted reproductive technology. Fertility and sterility. 2017. 107(4):954-60. (Florida, Michigan, Massachusetts) Luke, 2018
19. J. L. Marino, V. M. Moore, K. J. Willson, A. Rumbold, M. J. Whitrow, L. C. Giles and M. J. Davies. Perinatal outcomes by mode of assisted conception and sub-fertility in an Australian data linkage cohort. 2014. 9 (1): e80398 (Australian). Davies, 2012
20. K. Wisborg, H. J. Ingerslev and T. B. Henriksen. In vitro fertilization and preterm delivery, low birth weight, and admission to the neonatal intensive care unit: a prospective follow-up study. 2010. 94 (6): 2102-6.(Denmark) Wennerholm, 2013
21. B. Kallen, O. Finnstrom, K. G. Nygren, P. Otterblad Olausson and U.-B. Wennerholm. In vitro fertilisation in Sweden: obstetric characteristics, maternal morbidity and mortality. 2005. 112 (11): 1529-35.(Sweden) Wennerholm, 2013
