# Supplementary material for: Neonatal outcomes in singleton pregnancies conceived by fresh or frozen embryo transfer compared to spontaneous conceptions: a systematic review and meta-analysis
Source: Arch Gynecol Obstet. 2020 May 22;302(1):31–45. doi: 10.1007/s00404-020-05593-4 (PMC7266861; doi:10.1007/s00404-020-05593-4)
Supplement: Supplementary file 3 — Supplementary file3 (DOCX 109 kb) [file 404_2020_5593_MOESM3_ESM.docx]

Supplementary Appendix 03: Sensitivity analysis

Fresh embryo transfer pooled results considering only matched studies.

| PRETERM - Sensitivity analysis with matched studies (1)  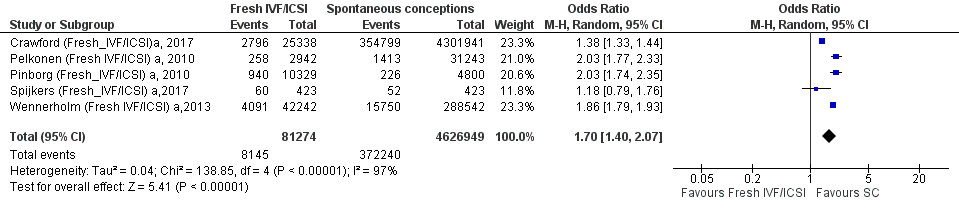  LBW - Sensitivity analysis with matched studies (1)  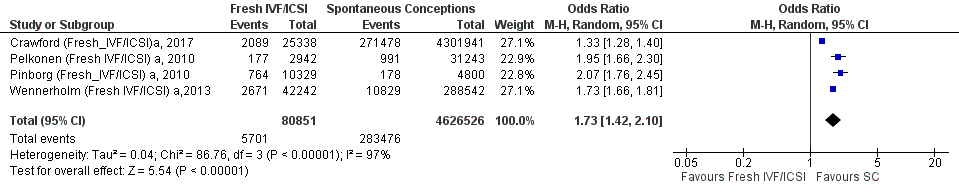  SGA - Sensitivity analysis with matched studies (1)  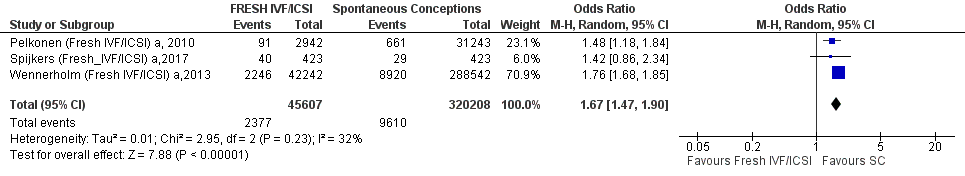  LGA - Sensitivity analysis with matched studies (1)  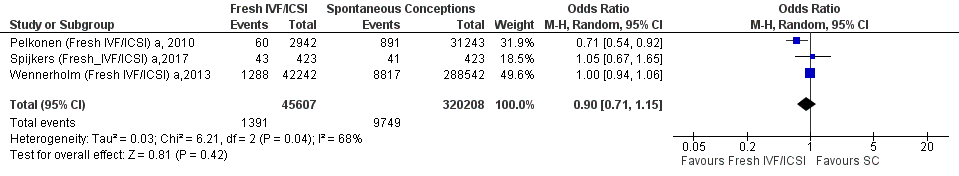  IVF only- PRETERM - Sensitivity analysis with matched studies (2)  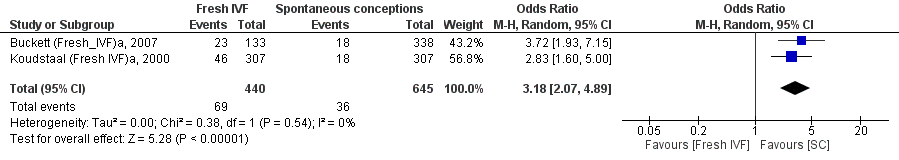 |
| --- |

Notes: (a) matched cohorts, (1) excluded Luke and Sazanova, (2) excluded Davies and Ernstad

Frozen embryo transfer pooled results considering only matched studies.

| PRETERM - Sensitivity analysis with matched studies (1)  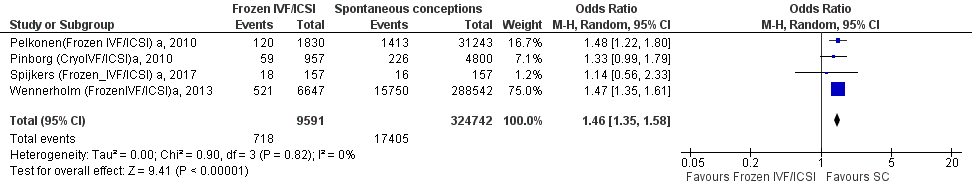  LBW - Sensitivity analysis with matched studies (1)  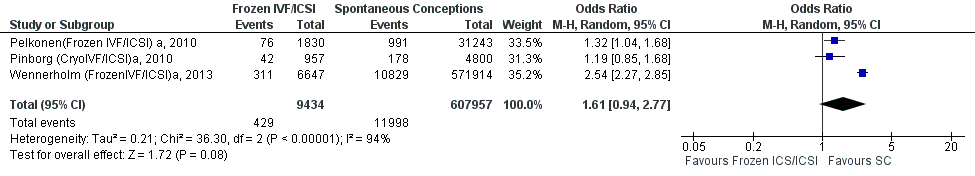  SGA - Sensitivity analysis with matched studies (1)  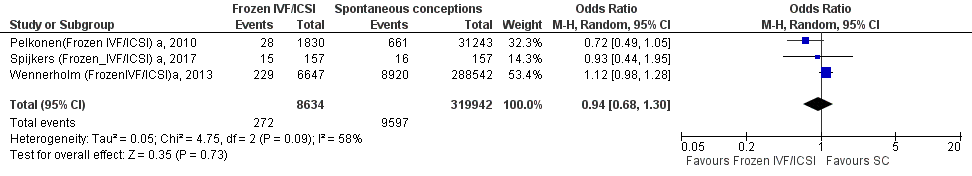  LGA - Sensitivity analysis with matched studies (1)  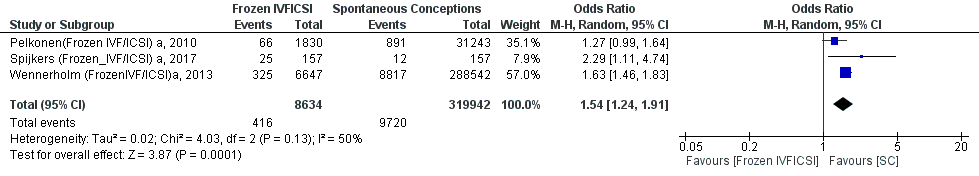 |
| --- |

Note: (a) matched cohorts, (1) excluded Luke and Sazanova
